# *XIST* drives X-chromosome inactivation and safeguards female extraembryonic cells in humans

**DOI:** 10.1038/s41467-026-78394-z

**Published:** 2026-10-22

**Authors:** Amitesh Panda, Léo Carrillo, Bradley Philip Balaton, Jeanne Brouillet, Solomon Nshemereirwe, Jarne Bonroy, Charbel Alfeghaly, Romina Facchinello, Sherif Khodeer, Nicolas Peredo, Ruben Boers, Gael Castel, Charlie London, Emmanuel Cazottes, Madeleine Moscatelli, Raissa Songwa Tchinda, Thi Xuan Ai Pham, San Kit To, Ryan Nicolaas Allsop, Yang Wang, Desislava Staneva, Peter J. Rugg-Gunn, Kathy K. Niakan, Joost Gribnau, Jean-François Ouimette, Claire Rougeulle, Vincent Pasque

**Affiliations:** 1 Department of Development and Regeneration, KU Leuven, Leuven, Belgium; 2 Leuven Institute for Single-cell Omics (LISCO), Leuven, Belgium; 3 Leuven Cancer Institute (LKI), Leuven, Belgium; 4 Epigenetics and Cell Fate, Université Paris Cité, CNRS, Paris, France; 5 Institut Curie, PSL University, Sorbonne Université, Université Paris Cité, CNRS UMR3244, Dynamics of Genetic Information, Paris, France; 6 VIB BioImaging Core Leuven, VIB Technologies, Center for Neuroscience Leuven, Leuven, Belgium; 7 VIB BioImaging Core Leuven, Department of Neurosciences, KU Leuven, Leuven, Belgium; 8 Department of Developmental Biology, Erasmus University Medical Center, Rotterdam, The Netherlands; 9 Epigenetics Programme, Babraham Institute, Cambridge, UK; 10 Loke Center for Trophoblast Research, Department of Physiology, Development and Neuroscience, University of Cambridge, Cambridge, UK; 11 Cambridge Stem Cell Institute, University of Cambridge, Cambridge, UK; 12 Present Address: Department of Genome Regulation, Max Planck Institute for Molecular Genetics, Berlin, Germany

**Keywords:** Epigenetic memory, Stem-cell differentiation, Cell lineage

## Abstract

X-chromosome inactivation (XCI) is essential for extraembryonic tissue growth and embryo development in mice. However, species-specific differences in its timing and regulation have left its role in human development unclear. Here, we show that the in vitro differentiation of naive human pluripotent stem cells to trophoblast stem cells and extraembryonic mesoderm cells is accompanied by XCI. The inactive X chromosome displays an atypical chromatin state, lacking DNA methylation and canonical heterochromatin marks. We demonstrate that extraembryonic differentiation and XCI are temporally linked. Using loss of function approaches, we found that *XIST* is required for XCI establishment in humans. Furthermore, we show that XCI is needed for the survival of human female extraembryonic cells. Together, these findings link XCI to the formation of human extraembryonic lineages, with important implications for human reproductive biology.

## Abstract (web summary)

X-chromosome inactivation (XCI) silences one of the two X chromosomes in females, but its role in human development is unclear. Here, the authors show that human extraembryonic differentiation triggers XIST-dependent XCI, important for cell survival.

## Introduction

X-chromosome inactivation (XCI) is the process that silences one of the two X chromosomes in female eutherian mammals^1^. In mice, XCI is triggered by the long non-coding RNA *Xist*, which recruits co-factors for transcriptional silencing, leading to a heterochromatic inactive X chromosome (Xi)^2–8^. XCI is established during early mouse embryonic development, first in an imprinted manner where it is always the paternal X chromosome that is inactivated in female preimplantation embryos and in extraembryonic tissues^5,6,9–12^. Impairing XCI establishment through *Xist* mutation results in early female lethality, which has been attributed to defects in extraembryonic tissues. The importance of *Xist* and XCI for extraembryonic development has thus been documented in mice^13–15^.

In contrast, the kinetics and importance of XCI for the development of human extraembryonic lineages remain poorly understood, and the role of *XIST* in these processes has never been addressed, despite potential consequences for embryo implantation, pregnancy success, and sex-biased diseases of developmental origin^16^. This is relevant as major differences exist between rodents and humans, both in terms of X chromosome status and developmental features^17^. In humans, *XIST* is expressed from the two X chromosomes during female preimplantation development without triggering XCI^18,19^. Petropoulos and colleagues suggested that instead of classical XCI, X-linked gene expression is partially reduced or “dampened” from both active X chromosomes in the preimplantation human embryo, a stage during which *XIST* accumulates on the X chromosomes in a dispersed configuration^19–21^. Despite initial debates over the observation of dampening^19,20,22^, various reports have now provided evidence of X-chromosome dampening in human primordial germ cells, preimplantation human embryos and in naive human pluripotent stem cells (hPSCs)^21,23–26^ and likely also in monkey embryos^25^.

In humans, XCI is established at peri-implantation stages with different dynamics depending on the lineage, occurring first in the trophectoderm^20,23,27^. Similar findings have recently been reported for Cynomolgus monkey embryos^25^. In addition, extraembryonic development differs markedly between mice and humans, both at the morphological and molecular levels, with humans having no extraembryonic ectoderm, in contrast to the mouse^28^. Moreover, the extraembryonic mesoderm is specified after implantation and before gastrulation in humans, while in mice, these cells are only specified after gastrulation^29–35^.

Addressing the key question of XCI mechanisms and requirements for human embryo development requires appropriate systems to model the establishment of XCI in humans^36^. Naive human embryonic stem cells (hESCs) have emerged as an attractive system^37,38^, as they represent the preimplantation human epiblast state, and carry two active X chromosomes (XaXa). In naive hESCs, *XIST* expression is typically observed mostly from one of the two X chromosomes, in contrast to the preimplantation embryo epiblast, where most female cells have two *XIST* clouds^18,19^. *XIST* has been shown to trigger X-chromosome dampening in naive hESCs^21,26,39,40^. *XIST*-coated X chromosomes are enriched in polycomb-mediated repressive histone modifications, histone H2A lysine 119 monoubiquitination (H2AK119ub), and histone H3 lysine 27 trimethylation (H3K27me3) in naive hESCs, as in embryos, despite their active states^26^. The role of *XIST* in the establishment of XCI in humans is currently assumed and remains to be formally tested.

Naive hESCs can be differentiated toward the postimplantation epiblast lineage, a process termed capacitation^41^. During this transition, cells acquire competence for somatic cell differentiation and reach a primed pluripotent stem cell state, analogous to the post-implantation epiblast^41^. Unlike their mouse counterparts, naive hESCs can also be differentiated into extraembryonic lineages such as postimplantation extraembryonic mesoderm cells (EXMCs) and trophoblast stem cells (TSCs)^42–46^, in addition to all three founding lineages of the embryo^47–50^. XCI occurs in the course of in vitro differentiation of naive hESCs toward TSCs, trophoblast organoids and during capacitation^51–53^. However, the functional and mechanistic interplay between XCI and extraembryonic fate induction has not been elucidated.

Here, we capitalized on in vitro cell fate induction to study the mechanisms and significance of XCI in early human development. We show that one X chromosome acquires multiple hallmarks of XCI in in vitro-derived extraembryonic cells, including gene silencing, exclusion of RNA polymerase II, a compact *XIST* RNA configuration and adopts an unusual chromatin state that differs from that of embryonic cells, with limited enrichment in heterochromatin hallmarks compared to the active X chromosome and to autosomes. We further reveal that the de novo establishment of human XCI is closely linked, in time, with the emergence of EXMCs and TSCs. We show, using an inducible loss of function approach, that XCI is dependent on *XIST* expression and that *XIST* impacts female extraembryonic cell survival. In addition to establishing that *XIST* is necessary for the establishment of XCI in humans, our work provides evidence of XCI contribution to female human extraembryonic cell development.

## Results

### XCI is established in human extraembryonic cells

**Fig. 1:**
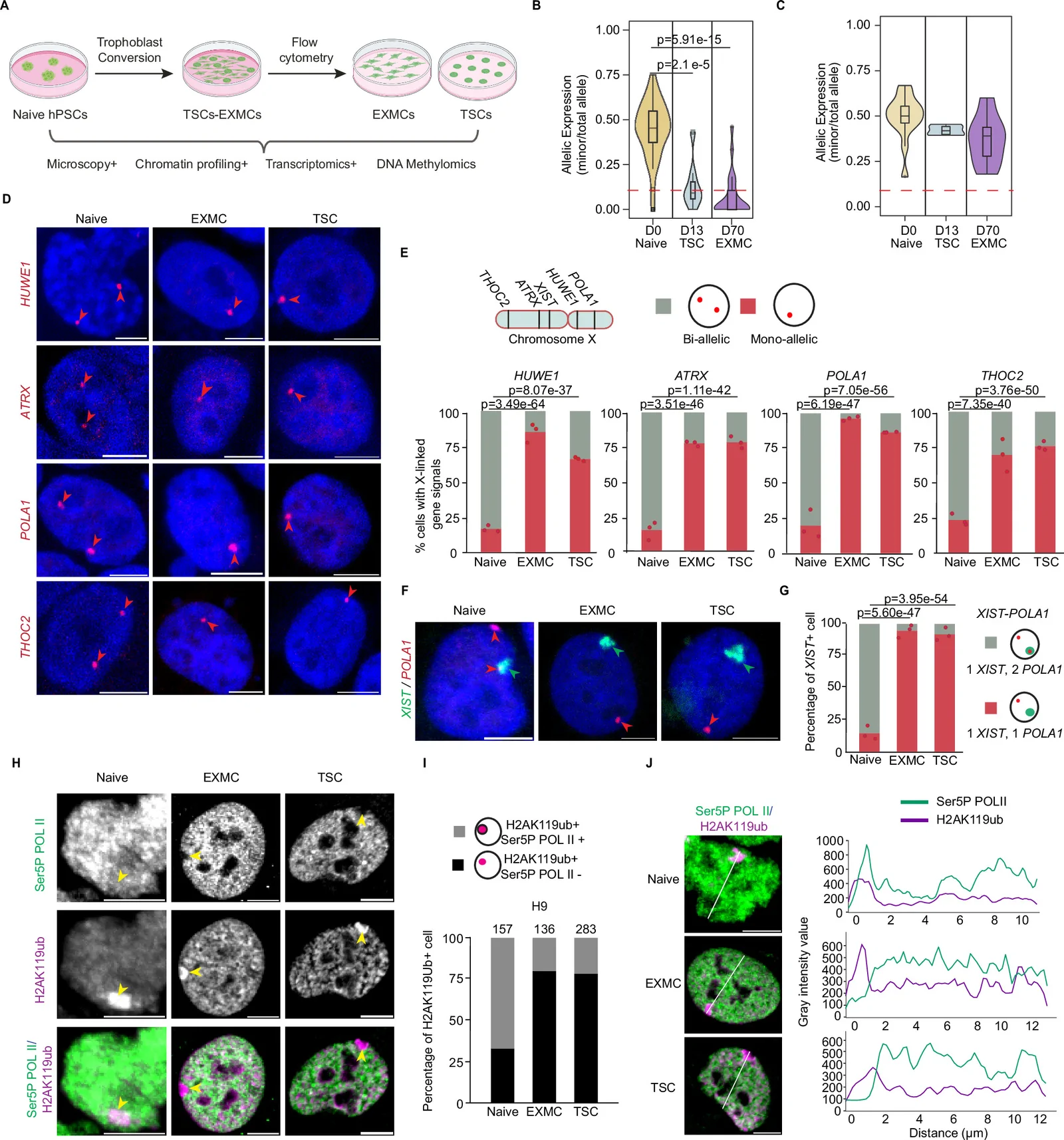
XCI is established in human extraembryonic cells. **A** Schematic of naive human pluripotent stem cell (hPSC) differentiation toward extraembryonic mesoderm cells (EXMCs) and trophoblast stem cells (TSCs), and approaches used to assay XCI. Created in BioRender. Pasque, V. (2026) https://BioRender.com/ceje9w6. **B** Violin plot of allelic expression from scRNA-seq data, calculated using the scLinaX pipeline^54^, showing XCI-subject genes in naive, day 13 TSCs, and day 70 EXMCs (reanalysis of published data)^44^. Two-sided *t*-test. Box plots show the median, 25th/75th percentiles, and whiskers to 1.5× interquartile range (IQR); points beyond whiskers are outliers. *N* = 42 genes. Dotted line indicates the silencing threshold (minor allele expression <10%). **C** As in (**B**), but only for escapee genes. **D** Representative RNA FISH images of *HUWE1*, *ATRX*, *POLA1*, and *THOC2* (Red) in H9 naive hESCs, EXMCs and TSCs. Scale bar, 5 µm. **E** Quantification of monoallelic (red) versus biallelic (gray) X-linked gene expression in H9 naive, EXMCs, and TSCs. Mean of 3 independent experiments (*n* = 3); Binomial logistic regression statistics were used with naive as the reference group, two-sided *p*-values shown. **F** Representative RNA FISH images of *XIST* (Green) and *POLA1* (Magenta) in H9 naive, EXMC and TSC. Scale bar, 5 µm. **G** Quantification of POLA1 puncta and XIST clouds in XIST-positive cells from (**F**). Mean of 3 independent experiments (*n* = 3); two-sided logistic regression was used, with two-sided *p*-values shown. **H** Representative immunofluorescence (IF) of Ser5-phosphorylated RNA polymerase II (Ser5P POL II, Green) and ubiquitinated histone H2A at lysine 119 (H2AK119ub, Magenta) in naive hESCs, EXMCs and TSCs. Scale bar, 5 µm. Arrowheads indicate the H2AK119ub-enriched X chromosome. **I** Quantification of H2AK119ub foci with or without Ser5P POL II overlap; nuclei count indicated above each bar. **J** Left: representative IF of Ser5P POL II (green) and H2AK119ub (magenta). Right: line plots of gray intensity for Ser5P POL II across the H2AK119ub cloud in single nuclei. Scale bar, 5 µm. Source data provided for (**B**, **C**, E, **G**, and **I**).

To investigate X-chromosome states in naive hPSCs and extraembryonic cell types, we differentiated female H9 naive hESCs into EXMCs and TSCs using trophoblast stem cell medium (Fig. 1A). After 30 days, we sorted EXMCs and TSCs using extraembryonic mesoderm (EXM) and trophoblast markers BST2 and ENPEP, respectively (Fig. S1A)^44,47^. Naive hESCs, EXMCs and TSCs consistently displayed the expected dome-shaped, mesenchymal, and cuboidal morphologies, respectively (Fig. S1B). We confirmed their identities using immunofluorescence (IF) for lineage markers (Fig. S1C) and bulk RNA sequencing (RNA-seq) (Fig. S1D), consistent with a previous study^44^.

To test if XCI is induced during extraembryonic fate induction, we performed allelic analysis of scRNA-seq data of naive hPSCs, day 13 TSC and day 70 EXMCs^44^ using scLinaX (single-cell level inactivated X-chromosome mapping)^54^. Allele-specific analysis is well-established in mice, where it takes advantage of the numerous and phased SNPs available in polymorphic strains^15,55–57^. Such analysis is more challenging in humans: SNPs are less abundant. The use of scLinaX, which operates directly on single-cell data to infer XCI status from available SNPs without requiring phased genomes, allowed us to overcome these limitations. We observed a significant decrease in the allelic ratio for genes expected to be subject to XCI in TSCs and EXMCs, but not in naive hPSCs (Fig. 1B). By contrast, genes subject to escape from XCI remained largely unchanged in their allelic ratios across cell types (Fig. 1C), collectively suggesting that XCI is established during the induction of naive hPSCs toward the extraembryonic lineages.

We also examined XCI using bulk RNA-seq, calculating the median allelic ratio per X-linked gene by dividing the least expressed allele by the most expressed one, yielding a biallelic expression ratio of one. In naive hPSCs, the average allelic ratio was slightly below one (Fig. S1E), as previously reported^21,26^. The median allelic ratio was lower in TSCs^51^, and in EXMCs it was intermediate, above that of TSCs, yet significantly lower than in naive hPSCs. In TSCs, genes known to undergo XCI in somatic cells showed a lower allelic ratio than escapee genes. Furthermore, overall mean X-chromosome expression was reduced in EXMCs and TSCs relative to naive hPSCs, while chromosome 7, of similar size and gene density, remained constant (Fig. S1F). However, bulk RNA-seq is inherently limited for studying XCI: unlike scRNA-seq, it cannot distinguish cells that have inactivated different alleles, is informative only under conditions of complete XCI skewing, and may underestimate XCI due to incomplete spread across the X chromosome. We therefore refrain from drawing definitive conclusions about XCI establishment from these bulk RNA-seq data.

To probe expression of X-linked genes at the single-cell level in extraembryonic cells, we used RNA fluorescent in situ hybridisation (RNA FISH) of four X-linked genes *HUWE1*, *ATRX*, *POLA1*, and *THOC2*, and observed a clear switch from biallelic expression in the naive pluripotent stem cell state to monoallelic expression in both extraembryonic cell types (Fig. 1D, E). These results showed silencing of one of two X chromosomes in TSCs and EXMCs for all four X-linked genes examined. Simultaneous RNA FISH analysis of *XIST* and *POLA1* confirmed *POLA1* expression from the active X chromosome and *XIST* expression on the inactive X chromosome (Xi) (Fig. 1F, G), with similar results in three additional hPSC lines, including one hESC and two human induced pluripotent stem cell (hiPSC) lines (ICSIG-1 hiPSCs, SIG-1 hiPSCs, and WIBR2-MGT hESCs), grown in two different naive PSC culture media 5iLA^58^ and 5iLA-PXGL (Fig. S1G, H). Moreover, quantifying *POLA1* signal intensity in naive hPSCs, we found significantly lower expression from the *XIST*-coated than the non-coated X chromosome, further supporting the occurrence of X-chromosome dampening in naive hPSCs, in line with previous studies (Fig. S1I, J)^21,24,26^.

In mice, the Xi can also be monitored at the single-cell level via exclusion of the elongating form of RNA Polymerase II, phosphorylated on Serine 5 (Ser5P POL II)^3^. We thus combined IF for Ser5P POL II with H2AK119ub or H3K27me3 to mark the *XIST*-coated X chromosome^24,26^. We detected a single focal enrichment of H2AK119ub and H3K27me3 in around 30–75% of cells, irrespective of cell state (Figs. 1H and S1K–Q)^24,26^. In naive hESCs, the Ser5P POL II signal was detected within the zone of H2AK119ub and H3K27me3 enrichment, consistent with an active X chromosome (Figs. 1I, J and S1L–Q). In contrast, in extraembryonic cells, Ser5P POL II was excluded from the H2AK119ub/H3K27me3 domain, suggesting the formation of a repressive compartment (Figs. 1H, I and S1L–Q). These results were confirmed by quantifying the intensity value of Ser5P POL II in the vicinity of H2AK119ub both in naive and extraembryonic cells (Figs. 1J and S1Q–R).

Taken together, these results show that the transition of naive hESCs to extraembryonic lineages in vitro is accompanied by XCI establishment, marked by X-linked gene silencing and the formation of a transcriptionally repressed compartment.

### Unusual chromatin remodeling accompanies XCI

Consistent with this, combined *XIST* RNA FISH with IF for H3K27me3 and H2AK119ub showed that the *XIST*-coated X chromosome is consistently marked by these repressive histone modifications in both naive and extraembryonic cells (Fig. S2A, B).

**Fig. 2:**
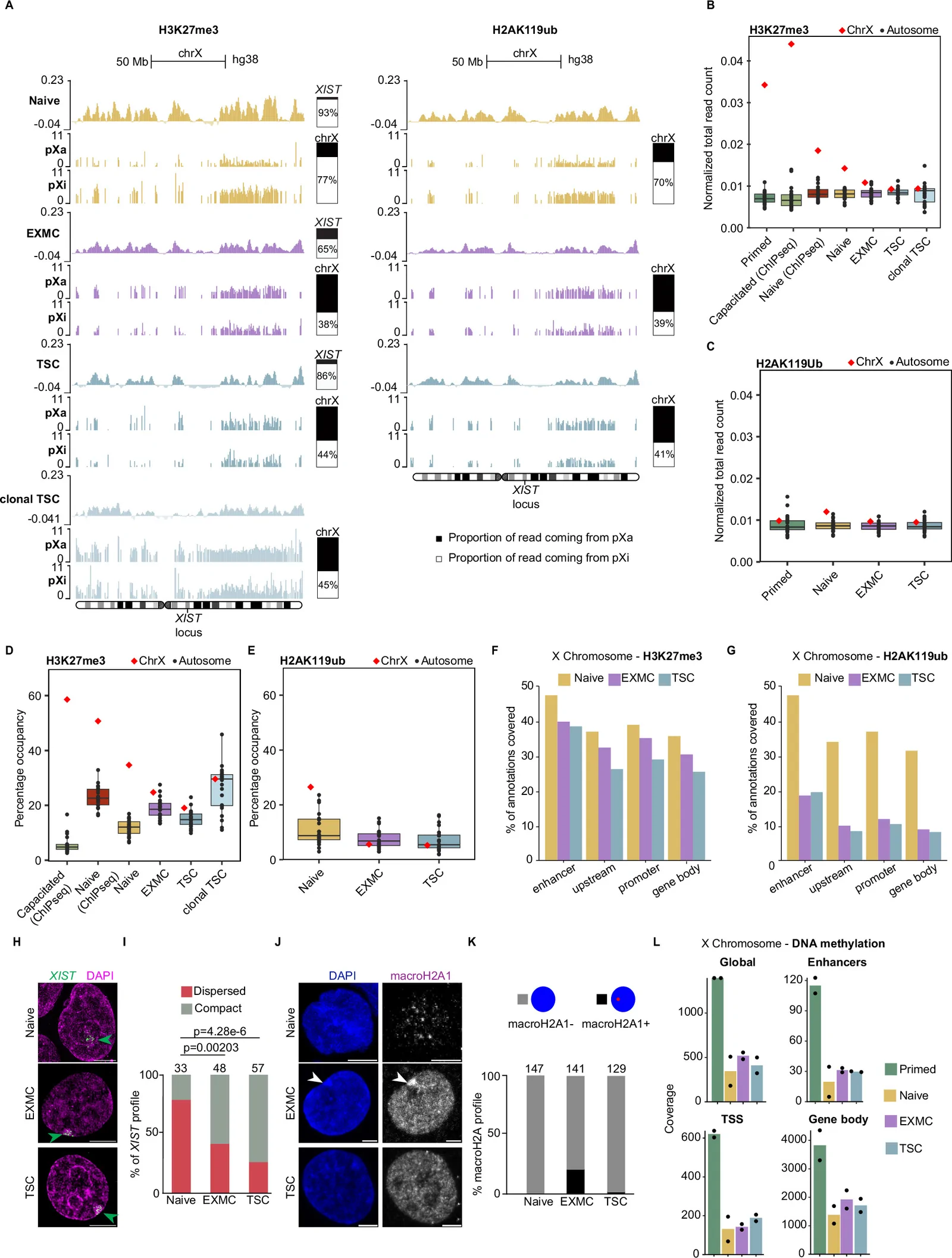
Unusual chromatin remodeling accompanies XCI. **A** CUT&RUN profiles (log2 enrichment over IgG) for H3K27me3 and H2AK119ub on the X chromosome in H9 naive human embryonic stem cells (hESCs), extraembryonic mesoderm cells (EXMCs) and trophoblast stem cells (TSCs). Allelic partitioning of reads is shown below each profile: primed-state active X (pXa) versus inactive X (pXi). Right: allelic XIST expression (top) and read quantification. CUT&RUN performed in duplicate (*n* = 2). **B** Total CUT&RUN or ChIP-seq H3K27me3 reads per chromosome, normalized by chromosome length across Primed hESCs^26^, Capacitated hESCs^26,53^, naive hESCs^26,53^, H9 naive hESCs^26^, EXMCs, TSCs, and clonal TSCs. For (**B**–**E**), boxplots display the median (center line), interquartile range (box), and values within 1.5 × IQR (whiskers); outliers omitted. **C** Total CUT&RUN H2AK119ub reads per chromosome, normalized by the chromosome length, in primed hESCs^26^, H9 naive hESCs^26^, EXMCs, and TSCs. **D** Percentage of H3K27me3 peak occupancy per chromosome in capacitated hESCs and naive hESCs (ChIPseq data), naive hESCs, EXMCs, TSCs, and clonal TSCs. **E** Percentage of H2AK119ub peak occupancy per chromosome in H9 naive hESCs, EXMC and TSCs. **F**, **G** Proportion (%) of genomic annotation covered by CUT&RUN H3K27me3 (**F**) and H2AK119ub (**G**) peaks in H9 naive hESCs, EXMCs, and TSCs. **H** Representative 3D-SIM images of *XIST* (Green) FISH and DAPI (Magenta) in H9 naive hESCs, EXMCs and TSCs. Scale bar, 5 µm. **I** Quantification of compact versus dispersed XIST clouds from (**H**); nuclei counted above each bar. Chi-square test; *p*-values shown; classification in “Methods”. **J** Immunofluorescence of macroH2A1 in H9 naive hESCs, EXMCs and TSCs. Scale bar, 5 µm. One WT hESCs line (*n* = 1). **K** Quantification of macroH2A1 clouds (frequency % of total cells) in H9 naive hESCs, EXMCs and TSCs. Nuclei counted above each bar. **L** CpG islands methylation score on the X chromosome of H9 naive hESCs, EXMCs, TSCs, and primed hESCs; total X chromosome (top left), enhancers (top right), transcription start sites (TSS) (bottom left), and gene body (bottom right). Number of replicates = 2 (*n* = 2). Source data are provided as a Source Data file.

To explore the chromosome-wide distribution of repressive hPTMs that are typically enriched on the Xi, including H3K27me3, H2AK119ub, and H3K9me3, we performed CUT&RUN in EXMCs and TSCs and compared to our previously published data of naive hESCs^26^. H3K27me3 and H2AK119ub enrichment profiles on the X chromosome were correlated across the three cell types, more strongly between EXMCs and TSCs, but not with H3K9me3 (Fig. S2C), and displayed similar distribution patterns throughout (Fig. 2A). Enrichment of these hPTMs at key developmental regulators correlated with gene expression as expected, confirming the validity of these datasets (Fig. S2D).

Despite the shared pattern, normalized enrichment and read distribution for H3K27me3 and H2AK119ub on the X chromosome were lower in EXMCs and TSCs compared to naive hESCs (Fig. 2A–C). To probe this further, we performed allelic analysis of the CUT&RUN data, using *XIST* RAP-seq data from clonal primed cells (with 100% skewed XCI^26,59^) together with H9 whole genome sequencing data to generate a partly phased X-chromosome haplotype (12,813 X-linked SNPs), and assign the Xi in the primed state (pXi). Both X chromosomes were equally accessible by CUT&RUN, as evidenced by equal representation of IgG control data (Fig. S2E). In naive hESCs, H3K27me3 and H2AK119ub marks were skewed towards the pXi (77 and 70%, respectively, of the X-linked reads), consistent with XIST being expressed solely from this chromosome^26^. In TSCs, however, despite a similar skewing of *XIST* expression (Fig. 2A), these marks were equally distributed on the two X chromosomes. Similar results were obtained on a clonal TSC sample, generated by single-cell sorting and expansion (Figs. S2F, G and S3A).

To further explore this, we compared the total number of reads for each condition, including previously published ChIP-seq datasets from naive and capacitated hESCs^26,53^. In naive hESCs, H3K27me3, and H2AK119ub were more enriched on the X chromosome compared to autosomes (Fig. 2B, C), as previously reported^26^. Such enrichment of H3K27me3 was even more pronounced in primed and capacitated hESCs, likely reflecting lower levels of this mark on autosomes in primed hPSCs (Fig. 2B)^60^. In contrast, in EXMCs and TSCs, H3K27me3 levels were similar on the X chromosome and autosomes (Fig. 2B, C). Similar observations were made when independently generated H3K27me3 CUT&RUN or CUT&Tag data from H9-derived TSCs from another laboratory (H9 TSC II), third trimester placenta-derived TSCs (CT27)^42^, and clonal TSCs were analyzed (Figs. 2D, E and S3B–D). Assessing more subtle changes, we found that while the coverage levels of these marks across the genomic features (enhancers, gene upstream regions, promoters, and gene bodies) were lower in EXMCs and TSCs compared to naive hESCs, their relative distribution was similar in all cell types (Fig. 2F, G). Focusing specifically on the subject to XCI genes, in naive hESCs, EXMCs, and TSCs, we found almost no H3K27me3 and H2AK119ub enrichment on the gene bodies of *ATRX*, *POLA1*, and *THOC2*, in naive hESCs, EXMCs, or TSCs, unlike in capacitated cells, whereas flanking regions tend to display higher enrichment in naive hESCs compared to EXMCs and TSCs (Fig. S3E, F).

Collectively, these results suggest that the inactive, *XIST*-coated X chromosome is not particularly enriched in H3K27me3 (and H2AK119ub) compared to the other X chromosome and to autosomes in TSCs and EXMCs, in contrast to capacitated and primed pluripotent stem cells.

To reconcile this apparent contradiction between IF and CUT&RUN, we first asked whether the visible IF enrichment of repressive marks reflects increased chromatin condensation. Quantifying DAPI intensity inside versus outside the *XIST*-coated X chromosome showed significantly higher condensation in extraembryonic cells compared to naive hESCs (Fig. S4A–C). In addition, H3K27me3 IF intensity was also elevated genome-wide across the nucleus in extraembryonic cells relative to naive hESCs (Fig. S4D, E). Together, these findings suggest that the accumulation of PRC-mediated repressive marks on the Xi observed by IF is due to X chromosome compaction rather than relative enrichment compared to other chromosomes.

This is consistent with the known differences in *XIST* distribution between naive and post-XCI states^20^. In human pre-XCI cells (preimplantation embryos and naive hESCs), *XIST* RNA adopts a dispersed configuration, while embryonic post-XCI cells such as primed pluripotent stem cells and fibroblasts show a compact *XIST* RNA cloud^20,21^. Using RNA FISH with conventional confocal microscopy and super-resolution three-dimensional structured illumination microscopy (3D-SIM), we measured the volume and diameter of the *XIST* RNA FISH signal in naive hESCs, EXMCs and TSCs. A single *XIST* cloud was detected in a vast majority of naive hESCs, as previously reported^21,24,26,61^, as well as in EXMCs and TSCs (Fig. S4F–I). We found that the transition of naive cells to extraembryonic cell fates was associated with the formation of more compact *XIST* clouds, as in post-XCI cells of embryonic origin (Figs. 2H, I and S4J, K and Supplementary Movies 1–3). 3D-SIM imaging further showed fewer *XIST* foci per nucleus in EXMCs and TSCs (around 30) than in naive hESCs (around 50) (Fig. S4L).

To further characterize the Xi chromatin state, we investigated macroH2A, a histone variant recruited in the late stages of XCI during differentiation of mouse embryonic stem cells^62–65^, and known to mark the Xi in primed hESCs^66,67^ by IF. Only a minority of EXMCs and TSCs displayed macroH2A enrichment on the Xi (20 and 1% , respectively) (Fig. 2J, K). We also assessed CpG island methylation, a mark commonly associated with Xi promoter regions in primed hESCs and somatic cells^66–69^, using methylated DNA sequencing (MeD-seq)^70^. We found that the DNA methylation levels on the X chromosome of EXMCs and TSCs were much lower than in primed hESCs and comparable to those of naive hESCs (Fig. 2L). These results suggest that the X chromosome is hypomethylated in EXMCs and TSCs compared to the Xi of embryonic lineage cells.

Collectively, these results showed that, in human extraembryonic cells, the Xi displays an atypical chromatin state, with the absence of most heterochromatin hallmarks of XCI.

### XCI is temporally linked with extraembryonic identity

**Fig. 3:**
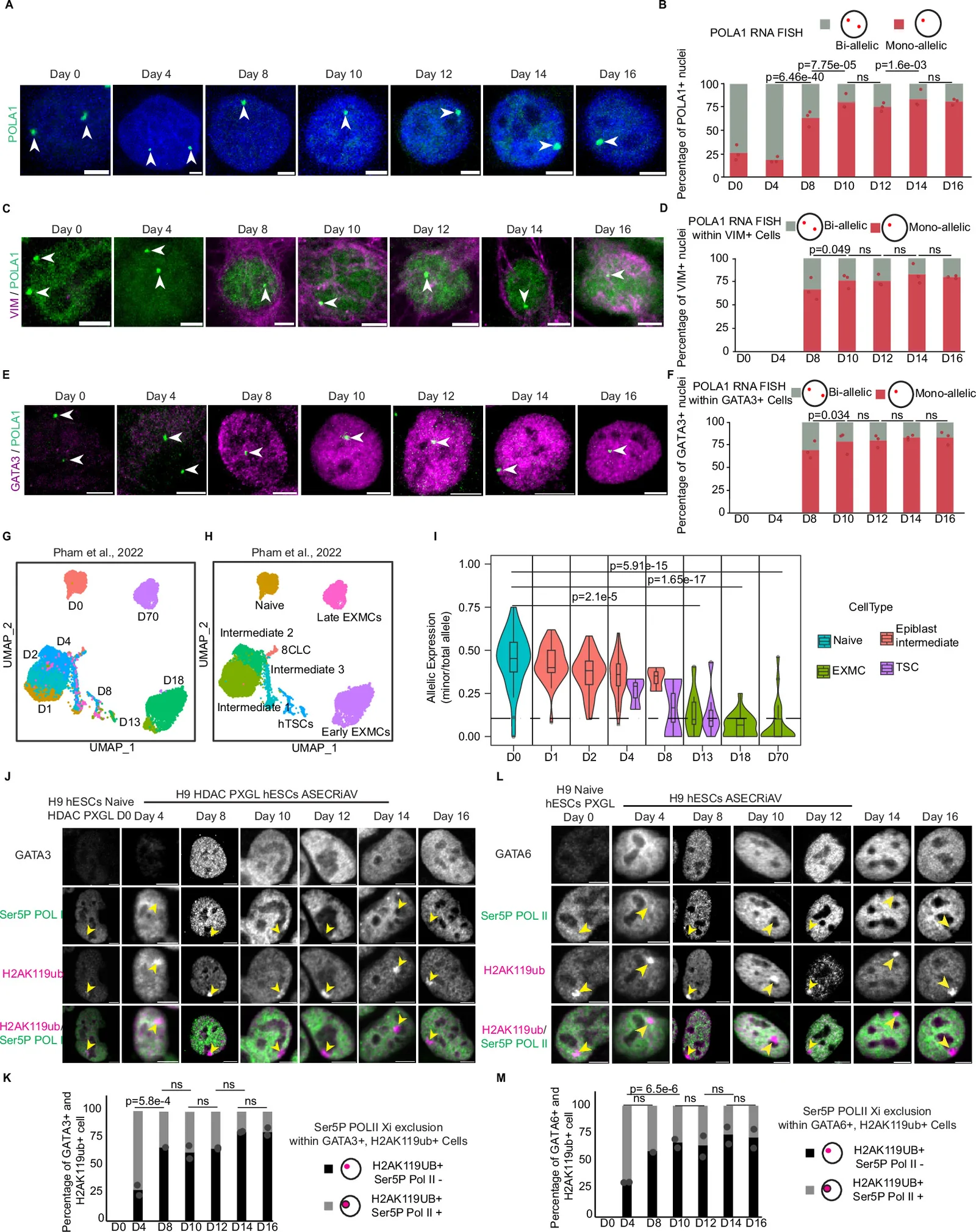
XCI is temporally linked with extraembryonic identity. **A** Representative FISH images of POLA1 puncta dynamics during differentiation of H9 naive human embryonic stem cells (hESCs) to extraembryonic mesoderm cells (EXMCs) and trophoblast stem cells (TSCs) across seven time points. Scale bar, 5 µm. **B** Quantification of cells with POLA1 puncta during differentiation from (**A**). Mean of 3 independent time-course experiments, with 3 FISH replicates. Two-sided binomial logistic regression; *p* < 0.05 significant (applies to **B**, **D**, **F**, **K**, **M**). **C** Representative IF-FISH of *POLA1* (green) and EXMC marker VIM (magenta) across seven time points. Scale bar, 5 µm. **D** Quantification within VIM-positive nuclei from (**C**), as in (**B**). **E** Representative IF-FISH of *POLA1* (Green) and trophoblast marker GATA3 (Magenta) across seven time points. Scale bar, 5 µm. **F** Quantification within GATA3-positive nuclei from (**E**), as in (**B**). **G** UMAP of time course scRNA-seq reanalysis of^44^ data during differentiation of ICSIG-1 naive hiPSCs to EXMCs and TSCs, including day 70 EXMCs, colored by days. **H** As in (**G**), colored by cell type. 8CLC 8 cell-like cells, hTSCs human trophoblast stem cells, and EXMC extraembryonic mesoderm cells. **I** Allelic expression from time-course scRNA-seq (scLinaX pipeline) for XCI-subject genes, shown for cell types with at least 2 informative SNPs per day (reanalysis of published data)^44^. Two-sided *t*-test. Box plots show median, 25th/75th percentiles, and whiskers to 1.5× interquartile range; points beyond whiskers are outliers. Dotted line indicates the silencing threshold (minor allele expression <0.1%). *N* = 42 genes. **J** Representative immunofluorescence of Ser5P POL II (green), H2AK119ub (magenta), and GATA3 (gray) across 7 time points. Scale bar, 5 µm. **K** Quantification (%) of nuclei with or without Ser5P POL II exclusion within the GATA3-H2AK119ub double-positive nuclei. Two-sided logistic regression test. **L** Representative IF of Ser5P POL II (green), H2AK119ub (magenta), and EXM lineage marker GATA6 (gray) across six time points. Scale bar, 5 µm. **M** Quantification (%) of nuclei with or without Ser5P POL II exclusion within the GATA6-H2AK119ub double-positive nuclei. Two-sided logistic regression test. For (**B**, **D**, **F**, **K**, and **M**), nuclei counts are in the source data file. Source data are provided as a Source Data file.

To link in time extraembryonic induction and the establishment of XCI, we first analyzed the kinetics of XCI during the transition from naive hESCs to EXMCs and TSC. Samples were collected on days 0, 4, 8, 10, 12, 14, and 16 of differentiation (Fig. 3A). RNA FISH for the X-linked gene *POLA1*, revealed a pronounced transition from biallelic to monoallelic expression between days 4 and 8 (data represent the mean from 3 independent time-course differentiation experiments followed by IF-FISH) (Fig. 3A, B). A similar trend was obtained with another X-linked gene, *HUWE1*, analyzed in H9 cells reset and maintained in different naive culture conditions, 5iLA^58^ and 5iLA-PXGL (Fig. S5A, B). We next integrated, by combining IF and RNA FISH, allelic analysis of *POLA1* expression with expression of trophoblast and embryo EXM markers, GATA3 and VIM, respectively^44^. GATA3+ and VIM+ cells were first detected at day 8 (Fig. 3C–F), as previously reported^44^. *POLA1* was monoallelically expressed in the majority of EXMCs and TSCs from day 8 onwards (Fig. 3B).

We also took advantage of our previously published single-cell RNA sequencing (scRNA-seq) time course data of naive hESCs to EXMCs and TSCs induction^44^ to narrow down the relationship between X-linked gene expression and cell identity. We calculated the X-to-autosome ratio as a readout for XCI. This ratio remained constant both in naive and epiblast intermediate cells up to day 4 of differentiation, then dropped significantly from day 8 onwards in cells identified as EXMCs and TSCs (Figs. 3G, H and S5C–E).

To further define the kinetics of XCI, we performed allelic analysis of scRNA-seq data comparing pre-XCI to post-XCI cell states using scLinaX^54^. We observed a significant decrease of the allelic ratio for genes expected to be subject to XCI^71^ from day 4 of differentiation (Fig. S5F). By contrast, genes expected to escape XCI^71^ remained biallelically expressed throughout the time course (Fig. S5F, G). When we further examined allelic X-linked gene expression in individual cell types, there was a significant decrease in EXMCs and TSCs by day 13 onwards (Fig. 3I). These results confirm the establishment of XCI during the transition of naive hPSCs to EXMC and TSC.

Time-course analysis of Ser5P POL II staining combined with that of GATA3 and GATA6 (as markers of TSCs and EXMCs, respectively) confirmed these results: the proportion of cells with Ser5P POL II exclusion from the H2AK119ub-marked chromosome increased most prominently between days 4 and 8 (Fig. S5H, I). We further combined the above staining with immunostaining of GATA3 and GATA6 to detect the dynamics of TSCs and EXMCs, respectively, during the differentiation (Fig. 3J–M). Immunostaining analysis revealed that GATA3 and GATA6 positive cells started appearing between days 4 and 10, coinciding with the occurrence of Ser5P POL II exclusion, which suggests the formation of a repressive compartment (Fig. 3J–M). Collectively, these results suggest that X-chromosome silencing is closely linked with acquisition of extraembryonic cell identity, occurring concurrently with or shortly after differentiation.

### Loss of *XIST* impacts human extraembryonic cell survival

**Fig. 4:**
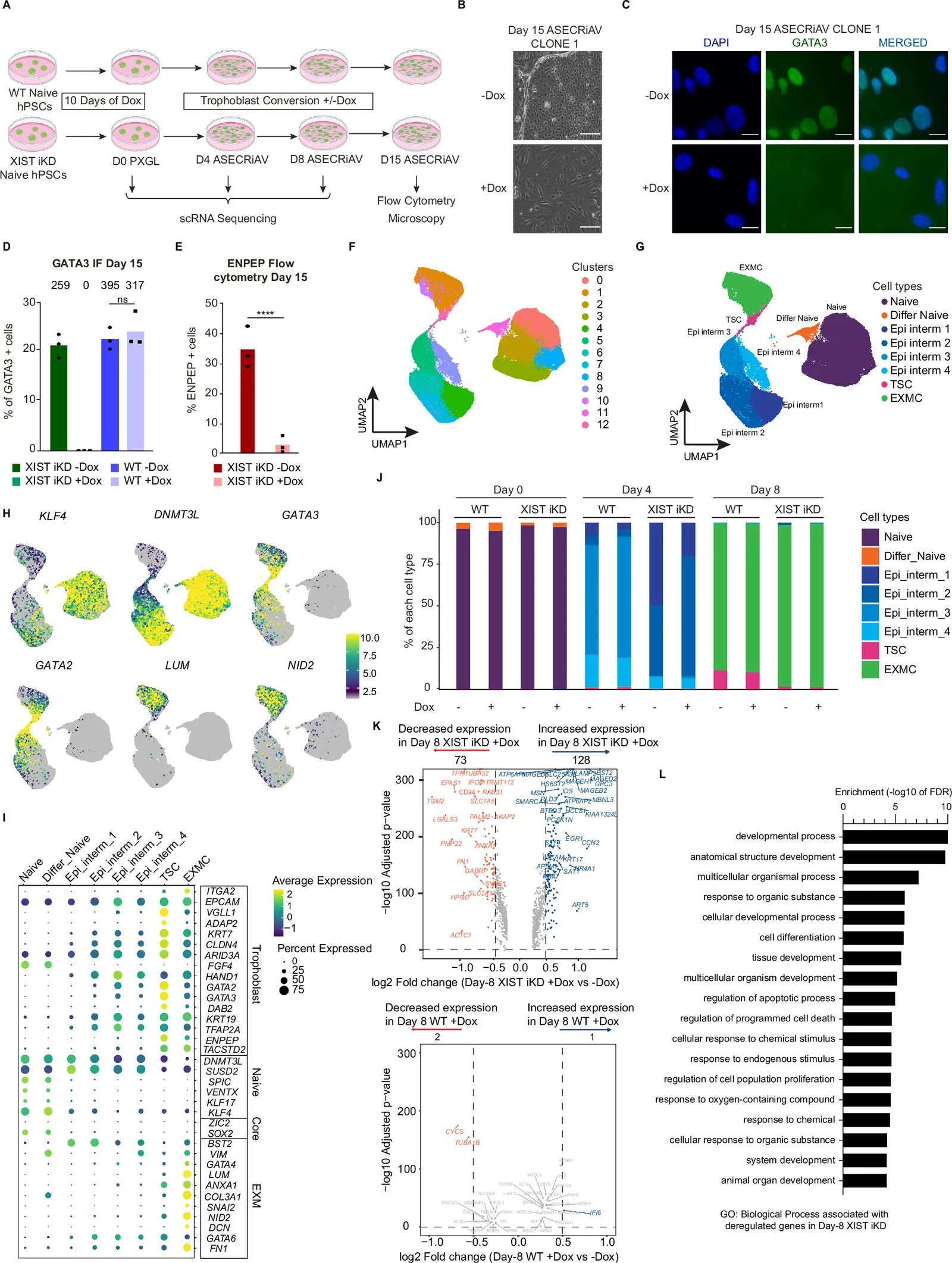
Loss of *XIST* impacts human extraembryonic cell survival. **A** Schematic of differentiation of wild type (WT) and *XIST* inducible knock down (iKD) H9 naive hESCs in trophoblast medium. Created in BioRender. Pasque, V. (2026) https://BioRender.com/skn518p. **B** Bright-field images of H9 *XIST* iKD clone-1 hESCs morphology at day 15 of trophoblast differentiation, with or without Doxycycline (Dox). Scale bar, 200 µm. (*n* = 3 clones; one shown, others in Fig. S6F). **C** Immunofluorescence of GATA3 (green) in cells from (**B**). DAPI in blue. Scale bar, 20 µm. (*n* = 3 clones, one shown here, others in Fig. S6G). **D** Proportion of GATA3-positive nuclei in WT and *XIST* iKD cells at day 15 of trophoblast differentiation, with or without Dox; nuclei counted above each bar. Each dot = one of 3 clones (*n* = 3). Two-sided *t*-test. *p*-values shown. **E** Flow cytometry quantification of ENPEP-positive cells at day 15 of trophoblast differentiation of *XIST* iKD hESCs with or without Dox. Two-sided Chi² test, *p* < 0.0001. Each dot = one of the three clones (*n* = 3). **F** UMAP of scRNA-seq time course during H9 WT and *XIST* iKD hESCs differentiation, with or without Dox under trophoblast conditions; 13 clusters shown by colors. Data from one clone per genotype (*n* = 1). **G** As in (**F**), colored by cell types. Naive epiblast, Differ Naive Differentiated naive epiblast, Epi interim (1, 2, 3, 4) Epiblast intermediate type (1, 2, 3, 4), TSC Trophoblast stem cell, and EXMC Extraembryonic mesoderm cell. **H** UMAPs from F showing the expression of *KLF4*, *DNMT3L*, *GATA3*, *GATA2*, *LUM*, and *NID2*. **I** Dot plot of marker gene expression for core pluripotency, naive pluripotency, trophoblast, and embryo extraembryonic mesoderm. **J** The proportion of each cell type during differentiation of naive hESCs into TSCs and EXMCs. Colored by cell types. **K** Differential gene expression (scRNA-seq) between day 8 *XIST* iKD (Top) and WT cells (Bottom) versus without Dox. Two-sided Wilcoxon rank-sum test, Bonferroni-corrected. Dashed lines indicate −log10 adjusted *p*-Value < 0.05 and log2 fold change < −0.5 or > 0.5. **L** Gene ontology enrichment of differentially expressed genes from day 8 *XIST* iKD cells, with and without Dox. Source data are provided as a Source Data file.

The temporal correlation between the establishment of XCI and the emergence of extraembryonic cells prompted us to investigate the functional interconnection between these two processes. To do so, we used an inducible XIST knockdown (*XIST* iKD) strategy, in which doxycycline (Dox) treatment triggers dCas9-KRAB-mediated *XIST* repression (Figs. 4A and S6A)^26^. A ten-day Dox (Dox+) treatment of naive hESCs achieved nearly complete repression of *XIST* in three independent clones (Fig. S6B–D), without affecting naive pluripotency gene expression (Fig. S6D) or proliferation relative to wild-type (WT) cells (Fig. S6E)^21,26^. By contrast, *XIST* repression had a pronounced effect during the differentiation of naive hESCs toward EXMCs and TSCs. *XIST*-depleted and WT cells were indistinguishable during the initial steps of differentiation. However, most of the *XIST*-depleted cells were lost by day 15, leaving only feeder cells, identifiable by the presence of chromocenters (Fig. 4B, C). Similar results were obtained in multiple *XIST* iKD clones (Fig. S6F). In the absence of *XIST*, no GATA3+ colonies could be observed at day 15, whereas GATA3+ cells were detectable in WT cells treated or not with Dox (Figs. 4D and S6G). The striking loss of trophoblast cells upon *XIST* repression was confirmed using flow cytometry analysis for the trophoblast marker ENPEP at day 15 (Fig. 4E). To establish if the observed phenotype was linked to the absence of *XIST* expression during the differentiation or was a consequence of *XIST* loss in naive hESCs, we included a condition where *XIST* was depleted in naive cells, followed by Dox removal from the media from day 0 of differentiation. This allowed for *XIST* re-expression, which partially rescued the phenotype, with around 18% of cells expressing ENPEP at day 15, compared to 35% in untreated conditions (Fig. S6H, I).

To gain deeper insights into the origin of the phenotype, we conducted time course scRNA-seq analysis in *XIST* depletion and control conditions by collecting cells at day 0, 4, and 8 of extraembryonic fate induction, before massive cell loss occurs (Fig. 4A). Uniform Manifold Approximation and Projection (UMAP) analysis of all conditions identified 13 clusters (Fig. 4F). At day 0, the majority of cells expressed markers of naive pluripotency and thus corresponded to naive hESCs, irrespective of the conditions, with a small fraction of differentiated cells, consistent with a previous study (Figs. 4F–J and S6J–K)^44^. By day 4, most cells had progressed into an epiblast intermediate state (Fig. 4F, G), as described previously^44^. Epiblast intermediates display decreased expression of several naive pluripotency genes, including *KLF4, SPIC*, and *VENTX* while maintaining expression of formative pluripotency genes (Figs. 4H, I and S6L). These epiblast intermediate populations formed four different clusters, which we termed epiblast intermediates 1, 2, 3, and 4, with a slight variation between the conditions (Fig. 4J), which may reflect a role for *XIST* in the transition between distinct pluripotent epiblast states. On day 8, EXMCs and TSCs became the only cell types (Figs. 4G–J and S6J–L) with no differences in their proportions between conditions (Fig. 4J). Altogether, these results suggested that naive hESCs lacking *XIST* expression can exit pluripotency and enter extraembryonic cell identities. However, cells did not survive upon passage.

To infer the gene network impacted by *XIST* loss, we identified differentially expressed genes (DEGs) between *XIST*-positive and *XIST*-negative cells at day 8, prior to *XIST*-depleted cell death. We found 73 genes with decreased expression and 128 genes with increased expression upon *XIST* depletion (Fig. 4K and Supplementary Data 1), compared with only three DEGs between Dox-treated and untreated WT cells, indicating that Dox treatment per se does not perturb gene expression (Fig. 4K and Supplementary Data 1). Gene ontology (GO) analysis revealed that deregulated genes in *XIST-*depleted cells are primarily implicated in development, cell differentiation, cell proliferation, and the regulation of apoptotic processes (Fig. 4L and Supplementary Data 1). These pathways are radically different from those corresponding to DEGs identified at day 0 and day 4, the latter instead relating to metabolic processes (Fig. S6M–O and Supplementary Data 2) and are in agreement with the phenotype observed.

Consistent with this, apoptosis-related gene expression was globally deregulated upon *XIST* loss, with more than 30 such genes showing altered expression in *XIST* KD conditions (Fig. S7A–D). Flow cytometry analysis of ENPEP and Annexin V showed that very few ENPEP+ cells were recovered in *XIST* iKD +Dox conditions and the few remaining displayed higher Annexin V staining, a marker of apoptosis, compared to control (Fig. S7E, F). Collectively, these results suggest that extraembryonic cells enter apoptosis in the absence of *XIST* expression.

### *XIST* is necessary for the establishment of human XCI

**Fig. 5:**
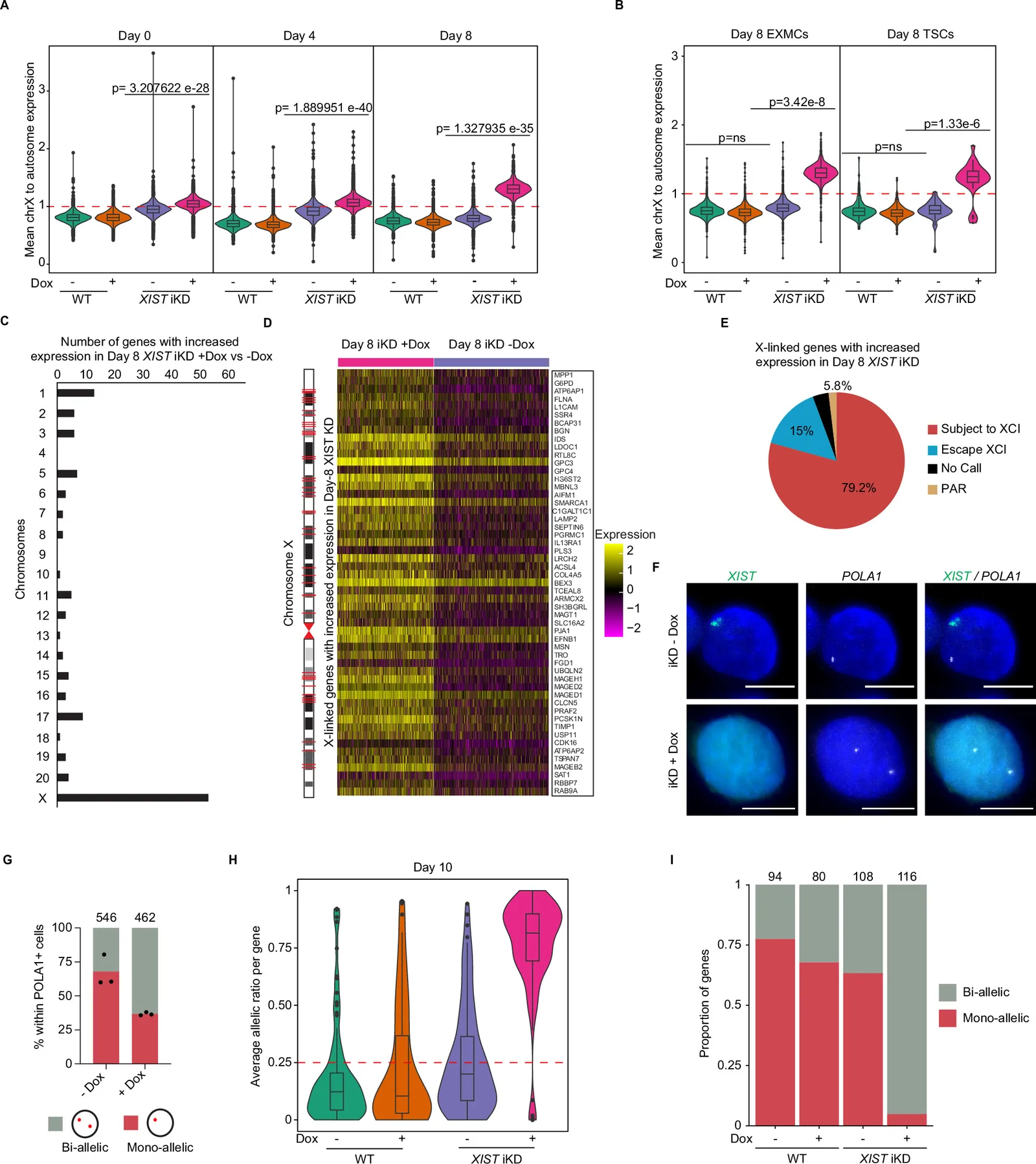
*XIST* is necessary for the establishment of human XCI. **A** Mean X-chromosome-to-autosome expression ratio at successive time points during differentiation of wild-type (WT) and *XIST* inducible knockdown (iKD) H9 hESCs, with and without Dox. Colored by conditions. Per cell, mean expression of X-linked genes was divided by mean expression of autosomes. Two-sided Kolmogorov–Smirnov tests on the dataset with 100 cells per condition with Bonferroni-corrected *p*-values are shown. scRNA-seq data from one clone per genotype (*n *= 1). Box plots show median (center line), 25th and 75th percentiles (lower and upper bounds of the box), and whiskers to 1.5 × the interquartile range; points beyond the whiskers are outliers. **B** Mean X-chromosome-to-autosome expression ratio in H9 TSCs and EXMCs at day 8 in WT and *XIST* iKD cells, with or without Dox. Two-sided *t*-test on a randomly downsampled dataset (24 cells per condition). Bonferroni-corrected *p*-values shown. scRNA-seq data from one clone per genotype (*n* = 1). Box plots, as in (**A**). **C** Number of differentially expressed genes (DEGs) per chromosome, day 8 *XIST* iKD without *XIST* (+Dox) versus with *XIST* (−Dox). **D** Right: expression of all X-linked DEGs in day 8 *XIST* iKD H9 cells with versus without *XIST*. Left: chromosomal map of DEG locations on the X chromosome. **E** Proportion of X-linked DEGs in day 8 *XIST-depleted* H9 hESCs by known XCI status category in somatic cells^72^. **F** Representative RNA FISH of *XIST* and *POLA1* in day 8 *XIST* iKD with Dox (*XIST*−) or without Dox (*XIST*+). Scale bar, 10 µm. **G** Proportion of biallelic and monoallelic *POLA1* signal on day 8 *XIST* iKD with Dox (*XIST*−) or without Dox (*XIST*+). Nuclei counted above each bar. Replicated across 3 independent clones. **H** Violin plot showing average allelic ratio per gene in day 10 WT and *XIST* iKD with or without Dox. Bulk RNA-seq performed on one WT and XIST iKD clone (*n* = 1). **I** Proportion of X-linked genes showing biallelic and monoallelic expression in day 10 WT and *XIST* iKD cells with or without Dox. Genes analyzed indicated above each bar. Source data are provided as a Source Data file.

While the requirement of *Xist* for the establishment of XCI has been well documented in mice^2,13^, the requirement of *XIST* for the establishment of XCI during human development has not been formally demonstrated due to the previous unavailability of cellular models for human XCI establishment during differentiation. We next probed more specifically whether the establishment of XCI required *XIST*. We aggregated scRNA-seq data per condition to generate pseudo-bulk datasets and calculated the mean X chromosome-to-autosome (X:A) expression ratio. This ratio was strongly elevated upon *XIST* depletion compared to control conditions, in particular at day 8 of differentiation, where it was most pronounced. A mild increase was also observed at days 0 and 4, which might be due to the role of *XIST* in X-chromosome dampening^26^ (Fig. 5A). Subcategorization of day 8 cells as EXMCs and TSCs showed a marked increase in X:A ratio upon *XIST* depletion in both cell types (Fig. 5B).

To test whether the elevated X:A ratio in the absence of *XIST* resulted from downregulated X-linked genes, we mapped to the genome, among the DEGs identified at day 8 of differentiation, the genes displaying increased expression in *XIST* repressed conditions (hereby referred to as increased expression genes (IEGs)). This revealed a strong enrichment of IEGs on the X chromosome (41.5%) relative to other chromosomes (Figs. 5C and S8A and Supplementary Data 3). This contrasts with the chromosomal distribution of IEGs identified at day 0 and day 4 of differentiation, of which only 5.6 and 11.8% were X-linked, respectively (Fig. S8B, C and Supplementary Data 3). The day 8 X-linked IEGs were distributed along the entire X chromosome (Fig. 5D and Supplementary Data 3). Moreover, most of these genes (79.2%) were defined as subjected to XCI in a comprehensive meta-analysis performed in somatic cells^72^ (Fig. 5E). Another 15% of IEGs were reported as escapees, which can be explained by a differential escaping pattern in embryonic versus extraembryonic lineages and/or by the influence of *XIST* on the expression levels of escapees, in line with a recent study in mice^73^. Collectively, these results show that human *XIST* is required for the establishment of chromosome-wide X-linked gene silencing during extraembryonic cell fate induction.

To further determine the effect of *XIST* depletion on XCI, we assessed the expression of the X-linked gene, *POLA1* at single-nucleus resolution by RNA FISH. The majority of day 8 *XIST-*depleted cells displayed biallelic *POLA1* expression, while this gene was silenced on the *XIST*-coated chromosome in control conditions (Fig. 5F, G). These results confirm *XIST* is required for X-linked gene silencing during EXMC and TSC induction. To probe, at the chromosome scale, the impairment in XCI establishment in the absence of *XIST*, we performed bulk RNA-seq and allelic analyses. Gene expression analyses confirmed the induction of EXMC and TSC markers in all four samples at day 10 of differentiation, the last time point before cells died (Fig. S8D). In this experiment, XCI was fully skewed towards one of the two alleles, enabling allele-resolution X-linked gene expression analyses by bulk RNA-seq (Fig. S9). In all samples in which *XIST* is expressed (WT −Dox, WT +Dox and *XIST* iKD −Dox), the mean allelic ratio was low, indicating monoallelic X-linked gene expression, hence XCI (Fig. 5H). This contrasted with *XIST* depleted samples, which displayed a higher allelic ratio of 0.8, reflecting biallelic gene expression, hence the lack of XCI. We then categorized genes as mono- or biallelically expressed using an allelic ratio threshold of 0.25. This confirmed that, in the presence of *XIST*, most X-linked genes were subjected to XCI at day 10, as inferred from their monoallelic expression pattern, and that this process is severely impaired in the absence of *XIST*, with most genes remaining biallelically expressed (Fig. 5I). These results demonstrate that *XIST* is required for the establishment of XCI in humans. They also suggest that the phenotype we observed resulted from impaired XCI.

## Discussion

Mice have long been the model of choice for studying XCI, with the assumption that findings would generalize across mammals. XCI studies in non-rodent species have, however, challenged this premise and highlighted the need to revisit the species-specific analysis of X-chromosome dosage regulation. Addressing these in humans is of clear interest, but has been hampered by limited biological resources and opportunities for functional studies in early developmental contexts.

The ability to derive and maintain naive hPSCs, which model preimplantation development in vitro, has paved the way for XCI studies in humans^74^. Our study establishes naive hPSCs as a powerful system to dissect the mechanistic and functional basis of de novo XCI establishment in humans. Here we exploited their property, the ability to give rise, in vitro, to post-implantation extraembryonic cells, TSCs and EXMCs, to link XCI and extraembryonic fate in humans. Our work opens broader avenues for understanding the role of XCI in lineage induction and in human embryo development.

Our results reveal that induction of extraembryonic fates from naive hPSCs models primate-specific biology, including the transition from X-chromosome dampening to XCI observed in early human embryogenesis^18,23,27^. Using approaches that provide gene-specific and chromosome-wide assessment of silencing, we confirmed that XCI is established in in vitro generated TSCs and EXMCs, consistent with previous findings^51,52^. Notably, the Xi in extraembryonic cells was not enriched in chromatin repressive hallmarks relative to the autosomes and to the Xa by CUT&RUN analysis, a result confirmed across independent TSC lines. The DNA hypomethylation we observed is consistent with X-linked promoter hypomethylation reported in human female placentas^67^. That the Xi was labeled with anti-H2AK119ub and H3K27me3 antibodies in EXMCs and TSCs in IF experiments might be explained by the higher density of histones on this chromosome^75^ and/or by the Xi 3D conformation^76,77^. We detected increased chromatin compaction of the Xi relative to autosomes in EXMCs and TSCs, as well as increased autosomal levels of H3K27me3 and H2AK119ub, which may explain the lack of detectable relative enrichment of these hPTMs by CUT&RUN analysis. The Xi was also reported to display an unusual chromatin status in mouse trophoblast giant cells in vivo^78^, raising the question of whether XCI plasticity in extraembryonic tissues reflects an evolutionary adaptation or simply the short lifespan of these tissues, in which repressive locks such as DNA methylation are dispensable.

The most striking conclusion of our study is that XCI supports female hESC-derived extraembryonic cell survival, revealing a functional interplay between X chromosome dosage and extraembryonic cell fitness in humans. We provide evidence that *XIST* is required for de novo establishment of XCI in human in vitro models of early development. It is interesting to note that *XIST*-deficient hESCs can exit naïve pluripotency. In mice, this step is blocked by the presence of two active X chromosomes, which interfere with MAPK, GSK3, and AKT signaling pathways^79^. This difference may relate to XCI kinetics: XCI is established concomitantly to pluripotency exit in mice^80^, while in the system we used, XCI establishment appears to be initiated between day 4 and 8 of differentiation, when cells have reached an intermediate epiblast stage. Because *XIST*-deficient cells fail to achieve X-chromosome dampening^21,26^, our results also suggest that dampening is dispensable for naïve pluripotency exit in humans, at least in vitro.

scRNA-seq analyses showed that differentiation toward extraembryonic fate can proceed even when XCI is blocked; by day 8, transcriptomic signatures and cell composition were indistinguishable between WT and *XIST*-deficient conditions. The TSC and EXMC states were, however, not maintained without XCI, and cells died within a few days. The lag between XCI establishment (between day 4 and 8 of differentiation) and lethality of XCI-deficient cells (between day 10 and 15) could reflect several non-mutually exclusive factors: a delay between rising mRNA levels and its functional consequences on cellular homeostasis, a dosage requirement for X-linked products only critical for differentiation events; or later inactivation of several X-linked genes responsible for the phenotype. This phenotype parallels the requirement of XCI in mice extraembryonic ectoderm progenitors development^13,14^, even if these cells have no equivalent in humans.

In conclusion, our work reveals the requirement of *XIST* for the establishment of XCI and its role for female extraembryonic cell survival in humans, at least in vitro. It suggests that subtle XCI misregulation during female human development could contribute to peri-implantation embryo loss through failure in establishing placental progenitor programs, a hypothesis that remains to be investigated in embryos.

### Limitations of the study

We report the consequences of impaired XCI during in vitro differentiation using specific differentiation media. The effect of *XIST* loss in other differentiation conditions, including recently reported fast EXMC induction protocols^81,82^, remains to be examined, also taking into consideration the fact that the efficiency of differentiation may vary between cell lines. The extent to which the phenotype would be reproduced in human embryo experiments remains to be determined, even if currently technically and ethically constrained. While naive hPSCs closely mimic the preimplantation naive epiblast, they exhibit monoallelic *XIST* clouds, whereas the epiblast in preimplantation embryos displays biallelic *XIST* clouds. This difference, together with broader heterogeneity in chromatin landscapes arising from variations in culture conditions across naive hPSC lines, represents an inherent limitation when modeling early human X-chromosome regulation using current naïve hPSC systems^20,21,24,26^, and may impact the interpretation of the finding. The consequences for XCI kinetics warrant further investigation.

In our in vitro system, TSCs and EXMCs exhibited varying degrees of XCI skewing. Skewing of XCI can arise through primary mechanisms, where one X chromosome is preferentially inactivated during XCI initiation, or through secondary mechanisms, which results from selection bias acting after random XCI has been established. It remains unclear whether the observed skewing in TSCs and EXMCs reflects primary or secondary skewing, or a combination of both. Future allele-specific single-cell approaches could help distinguish these mechanisms.

A major limitation in resolving allele-specific XCI states in this study stems from the absence of phased parental genome sequences for the naive hPSC lines used. By contrast, mice remain a powerful model system for allele-resolution analyses, owing to the availability of strains with mixed backgrounds, numerous SNPs, and phased parental genomes. SNPs are available for H9 naive hPSCs, enabling allelic analysis, but these are not phased. This limitation was partly but not fully solved in our study using RAP-seq data and scLinax. Compounding this, bulk measurements cannot resolve cell-to-cell heterogeneity arising from random XCI and partial skewing, as a mixed population carrying opposite active X chromosomes may appear biallelic at the population level. Additionally, it would be informative to determine the fraction of biallelic expression in the X chromosome in individual cells at different time points.

Finally, several limitations point to important directions for future work. The XIST iKD system enabled temporal control of XIST depletion and allowed us to dissect the role of XIST specifically during differentiation. Constitutive XIST knockout models could complement these findings by capturing developmental consequences from the onset of XCI. We currently cannot reliably analyze X-chromosome upregulation in humans, the process by which cells compensate for gene dosage imbalance between the single active X chromosome and diploid autosomes^56,57,83^, due to the absence of robust allele-specific expression methods in humans. Additionally, the absence of matched male cells represents a limitation for dissecting sex-specific dosage compensation mechanisms. Systematic establishment and characterization of such models will be an important priority for future studies.

## Methods

### Resource availability

#### Lead contact

Further information and requests for resources and reagents should be directed to and will be fulfilled by the lead contacts, Claire Rougeulle (claire.rougeulle@curie.fr) and Vincent Pasque (vincent.pasque@kuleuven.be).

### Materials availability

All stable reagents generated in this study are available from the lead contact without restriction except for human embryo-derived cell lines and hiPSC lines and their derivatives, for which permission must be requested from WiCell, Sigma, or Dr. Rudolf Jaenisch and a material transfer agreement and other documents required by local legislation and institutional policies must be completed.

### Experimental model and subject details

#### Ethics

Work with human embryonic and induced pluripotent stem cells to model early human development was approved by the UZ/KU Leuven ethics committee (S52426, S64962, S66185, S66184, and S66375) and also by the Flemish Government (SBB 219 2020/0435). Work with mouse embryos for mouse embryonic fibroblast was approved by the UZ/KU Leuven ethics committee (P170/2019).

Experiments were carried out using the female H9 hESCs obtained from the WiCell Research Institute. Research on hESCs has been approved by Agence de la Biomédecine and informed consent was obtained from all subjects.

### Cell lines

#### Human primed pluripotent stem cell culture

Human primed pluripotent stem cells were cultured as previously described in ref. ^44^. H9 hESCs (WiCell#WA09), Sigma hiPSCs (Sigma#iPSC EPITHELIAL-1-IPSC0028, SIG-1) inducible Cas9 Sigma hiPSCs (Sigma#iPSC EPITHELIAL-1-IPSC0028, ICSIG-1) and WIBR2 29M-GP26-TN9 hESCs^84^ were grown with or without feeder conditions, in normoxia conditions (5% CO_2_), and under humidified conditions at 37 °C. In feeder-free conditions, cells were cultured in pre-coated geltrex (Thermo #A1413302) tissue culture treated plates in complete E8Flex medium (Stemcell technologies). Cells were dissociated into smaller clumps every 5–6 days by incubating 5 min at room temperature in Versene. In feeder-dependent conditions, primed hESCs were grown on 0.1% gelatin coated plates with mitomycin-treated mouse embryonic fibroblast (MMC-MEF) feeders in knockout serum replacement (KSR) primed medium containing 77.5% of DMEM/F12 (Gibco, 31330-038), 15% FBS (Gibco, 10270106), 5% KSR (Gibco, 10828028), non-essential amino acid (Gibco, 11140050), 2 mM L-glutamine (Gibco, 25030081), Penicillin-streptomycin (Gibco, 15140-122), 0.1 mM β-mercapto-EtOH (Gibco, 31350-010) and adding 10 ng/ml FGF2 (Peprotech) freshly every day. Cells were passaged every 6–7 days using a 20 min incubation in collagenase (ThermoFisher 17104019). Media was changed every day.

#### *XIST* iKD clonal lines

A sgRNA targeting the *XIST* promoter was designed using the web-based tool CRISPOR and cloned into the PB_rtTA_BsmBI vector (Addgene, #126028)^85^. The sgRNA sequence can be found in the resource table. Using the Amaxa 4D-NucleofectorTM system (Lonza), one million H9 primed hESCs were transfected with 1.5 μg of PB_tre_dCas9_KRAB (Addgene, #126030), 0.75 μg of PB_rtTA_BsmBI and 1.5 μg of piggyBac transposase^86^. Cells were then treated with G418 (350 μg/mL) and hygromycin (350 μg/mL) until separate colonies were obtained. The number of random insertions in the genome was verified by qPCR and clones with the lowest insertion number (*n* = 2) were used for further experiments. Three independent primed H9 hESCs clones (named clone 1, clone 2, clone 3) and one WT clone (that has not been transfected) were then used.

### Human primed to naive resettings

#### PXGL HDAC resetting

WT and *XIST* iKD naive H9 hESCs were generated by chemical resetting of the H9 primed hESCs using a protocol previously described with small modifications^39,87^, and cultured on MMC-MEF. PXGL naive hESCs were cultured in PXGL medium consisting of a 1:1 mixture of DMEM/F12 (Thermo #31330038) and Neurobasal media (Thermo #21103-049) supplemented with 0.5% N2 supplement (#17502048), 1% B27 supplement (#17504044), 2 mM L-glutamine (#25030081), 100 μM β-mercaptoethanol (#31350-010) and 1× penicillin–streptomycin (#15140-122) (all from Gibco, Thermo Fisher Scientific) as well as 1 μM PD0325901 (Axon Medchem, 1408; CAS: 391210-10-9), 2 μM XAV939 (Cell guidance systems, SM38-10; CAS: 284028-89-3), 2 μM Gö6983 (Tocris, 2285; CAS: 133053-19-7), 10 ng/mL human LIF (Peprotech, 300-05). Naive hESCs were routinely split as single cells every three days at a ratio of 1:3 using Accutase (Stemcell Technologies, 07920) and plated in fresh media supplemented with 10 μM of Y-27632 (Stemcell Technologies). For *XIST* depletion, *XIST* iKD hESCs were treated with 1 μg/mL Dox for 10 days then either treated or untreated for subsequent culture with the same concentration of Dox.

#### KLF4 mRNA resetting

The resetting of human primed hESCs or hiPSCs was performed as previously described in ref. ^44^. In summary, starting from day 1 or day 2 after seeding primed hESCs in E8 onto Geltrex, cells were lipofected daily with KLF4 mRNA (Miltenyi, 130-101-115) for 9 days^88^. 2 μl KLF4 mRNA were diluted in 250 μL Opti-MEM (Gibco, 31985-047) and 6 μl of lipofectamine RNAimax (Invitrogen, 13778075) in another 250 μL of Opti-MEM for a single well of a 6-well plate. Then, the diluted mRNA was added to the diluted lipofectamine and incubated for 20 min at room temperature. After the medium was exchanged for 1.5 mL of fresh E8 flex, the mixture was added dropwise. After 10 days of lipofection, cells were passaged with Versene onto MMC-MEF feeders in E8 flex and transfected again. Starting from the following day, the medium was switched to t2iLGö (see below) supplemented with 10 µM Y-27632 (Tocris, 1254), and cells were transferred to hypoxia, while the lipofection was repeated every day for another 5 days. The naive t2iLGö medium contains a 50:50 mixture of DMEM/F12 (Gibco, 31330-038) and Neurobasal medium (Gibco, 21103-049), supplemented with 2 mM L-glutamine (Gibco), 0.1 mM β-mercaptoethanol (Gibco, 31350-010), 0.5% N2 supplement (Gibco, 17502-048), 1% B27 supplement (Gibco, 17504-044), 1% Penicillin-streptomycin (Gibco, 15140-122), 10 ng/ml human LIF (PeproTech, 300-05), 250 μM L-ascorbic acid (Sigma-Aldrich, A4544-100G), 10 μg/ml recombinant human insulin (Sigma, I9278-5ML), 1 μM PD0325901 (Axon Medchem, 1408), 1 μM CHIR99021 (Axon Medchem, 1386), 2.5 μM Gö6983 (Tocris, 2285)^88^. After a total of 15 days of transfection, cells were passaged with Accutase (Sigma-Aldrich, A6964-100ML) on the following day onto fresh feeders and continued to be cultivated in PXGL.

#### 5iLA resetting

To convert primed hESCs to naive hESCs, trypsinized primed hESCs were seeded onto gelatin-coated MMC-MEF feeders tissue culture treated 6-well plates and cultured with human KSR primed medium along with 10 μM of Y-27632 (Tocris, 1254) in a humidified normoxia incubator (5% CO_2_) for two days. On the third day and after giving a wash with Phosphate-buffered Saline (PBS (Gibco, 10010-015)) the medium was changed to 5iLA medium composed of 1:1 DMEM/F12 (Gibco, 31330-038) and Neurobasal (Gibco, 21103-049), 1% N2-supplement (Gibco, 17502-048), 2% B27 supplement (Gibco, 17504-044), 20 ng/ml recombinant human LIF (PeproTech, 300-05), 2 mM L-glutamine (Gibco, 25030-081), 1% non-essential amino acid, 0.1 mM β-mercaptoethanol (Gibco, 31350-010), 1× Penicillin-streptomycin (Gibco, 15140-122), 50 μg/ml BSA (Sigma-Aldrich, A3059) and supplemented with 5 inhibitors: PD0325901 (Stemgent, 1 μM), IM-12 (Enzo, 1 μM), SB590885 (R&D systems, 0.5 μM), WH-4-023 (A Chemtek, 1 μM), Y-27632 (Tocris, 10 μM), and Activin A (Peprotech, 20 ng/ml) and grown in a humidified incubator in hypoxia condition (5% CO_2_ and 5% O_2_) at 37 °C. After an initial wave of cell death around day 10–13, dome-shaped naive colonies started appearing. These cells were passaged into single cells every 4–5 days using 5 min incubation in Accutase (Sigma-Aldrich, A6964-100ML) at 37 °C.

For some experiments, H9 and WIBR2-MGT naive hESCs that were derived and cultured in 5iLA conditions were switched at passage 12 into PXGL naive medium for stable maintenance and expansion.

### Human naive pluripotent stem cell culture

Naive hESCs (H9 hESCs, WIBR2-MGT (converted from WIBR2 29M-GP26-TN9 hESCs) and Sigma hiPSCs) were cultured on MMC-MEF feeders in 5% O_2_ and 5% CO_2_ incubators under humidified conditions at 37 °C as previously described in ref. ^44^. Unless or otherwise stated, naive hESCs were cultured in the PXGL medium. For the experiments in Figure S1G, H and S5A, B, celss were cultured in 5iLA medium. Naive hESCs were passaged every 4–5 days at a ratio of 1:2 or 1:3 by single-cell dissociation with Accutase (Sigma-Aldrich, A6964-100ML), followed by filtering through a 40 μm cell strainer (Corning, 352340).

### Mouse feeders

MEFs were isolated from E14.5 pregnant WT C57/Black 6 mice. Male embryos were selected based on sex genotyping PCR and mitotically inactivated with Mitomycin C (Bioconnect). MEFs were cultured and harvested in a humidified incubator at 37 °C and in 5% CO_2_ by using filter-sterilized MEF medium consisting of 90% DMEM (Thermo #41966-029) supplemented with 10% FBS (Thermo #10270106), 1% Glutamax (Thermo #35050061), 1× Penicillin-streptomycin (Thermo #15140-122), 1× non-essential amino acid (Thermo #11140050), and 0.1 mM β-mercaptoethanol) and on 0.1% gelatin-coated tissue culture-treated plates.

### Naive hPSCs to EXMC and TSC fate induction

Human naive to EXMC and TSC differentiation were carried out using the following previously described protocols for TSCs^42,44–48^. Naive hESCs were seeded in such a way that expecting at least 70–80% confluency by day 2 on MMC-MEFs after dissociating in single cells using TrypLE (Thermo Fisher, 12605-010) for 15 min at 37 °C in their respective naive culture conditions supplemented with 10 μM Y-27632 (Tocris, 1254). The next day, after a wash with 1× PBS (Gibco, 10010-015), the media was switched from naive to ASECRiAV medium^42^, consisting of DMEM/F12 (Gibco, 31330038) supplemented with 0.3% BSA (Sigma, A3059), 0.2% FBS (Gibco, 10270-106), 1% Penicillin-streptomycin (Gibco, 15140-122), 1% insulin-transferrin-selenium-ethanolamine-X 100 supplement (Gibco, 51500056), 1.5 μg/ml L-ascorbic acid (Sigma, A8960), 0.5 μM A83-01 (Peprotech, 9094360), 1 μM SB431542 (Axon Medchem, 1661), 50 ng/ml hEGF (Miltenyi Biotec, 130-097-750), 2 μM CHIR99021 (Axon Medchem, 1386), 0.8 mM Valproic acid (Sigma, V0033000), 0.1 mM β-mercapto-EtOH (Gibco, 31350-010) and 5 μM Y-27632 (Tocris, 1254) or ACE medium^47^ consisting of 1:1 DMEM/F12 and Neurobasal, 0.5% N2-supplement, 1% B27-Supplement, 2 mM L-Glutamine, 0.1 mM β-mercaptoethanol, 1× penicillin-streptomycin, 1 μM of A83-01 (Peprotech, 9094360), 2 μM CHIR99021 (Axon Medchem, 1386), 50 ng/ml hEGF (Miltenyi Biotec, 130-097-750) and 5 μM Y-27632 (Tocris, 1254). The medium was changed every two days supplemented with 5 μM Y-27632. From passage one onwards, both EXMCs and TSCs were cultured and maintained on 5 μg/ml Collagen IV or iMatrix 511 silk (Laminin-E8) coated tissue culture-treated plates in hypoxia conditions (5% O_2_ and 5% CO_2_) and passaged every 5 days at 1:3 or 1:6 splitting ratios. Collagen IV-coated cell culture plates were coated overnight at 37 °C and iMatrix 511 silk coated plates were coated for 1 h. The proportion of EXMCs and TSCs varies from experiment to experiments and can be separated and cultured independently using flow cytometry for trophoblast and extraembryonic mesoderm marks.

### *XIST* iKD naive hPSCs to EXMC and TSC fate induction

Naive *XIST* CRISPRi hESCs were cultured in PXGL medium supplemented with 1 µg/ml doxycycline for 10 days to ensure *XIST* transcriptional repression. The differentiation into EXMCs and TSCs was carried out as previously described, with minor modifications: naive hESCs were dissociated in single cells using Accutase (Stemcell, 07920) for 5 min at 37 °C. 15,625 cells/cm^2^ were plated on MMC-MEFs in PXGL supplemented with 10 μM Y-27632 (Tocris, 1254). The next day, after a wash with 1× PBS (Gibco, 10010-015), the media was switched from PXGL/PXGL + Dox to ASECRiAV/ASECRiAV + Dox medium^42^. The medium was changed every day and cells were passaged every 5 days at 1:3 dilution, plated on MMC-MEFs.

### Method details

#### Immunofluorescence

IF staining was performed as described previously^44,89^ with little modification. Cells were grown on 0.1% gelatinized 18-mm round coverslips with or without feeders. The next day, cells were fixed in 4% paraformaldehyde (PFA) diluted in 1× PBS for 10 min at room temperature in the dark, permeabilized with 0.5% Triton ×-100 in PBS for 10 min and washed twice with 0.2% Tween20 in PBS (PBST) for 5 min each before proceeding to the staining. After this step, cells were either stored at 4 °C or directly subjected to staining. However, we noted that the H3K27me3 does not last long at 4 °C after fixation. Primary and secondary antibodies were diluted in a blocking buffer containing mainly PBST with 5% normal donkey serum and 0.2% fish skin gelatin. A list of antibodies can be found in the resource table. Cells on coverslips were incubated at 4 °C with the specific primary antibodies in blocking solutions (1:100 dilution for all antibodies except 1:50 dilution for NANOG, 1:40 dilution for FOXA2), and after that washed three times with PBST each 5 min. After that samples were incubated with the appropriate corresponding fluorophore conjugated secondary antibodies in blocking buffer (1:500 dilution) for 1 h in the dark, washed 2 times with PBST 5 min each, washed with 0.002% DAPI (Sigma-Aldrich, D9542) solution in PBST and a last wash again with PBST for 5 min. The coverslips were mounted in Prolong Gold reagent with DAPI after a final wash in PBST. Mounted coverslips were kept at room temperature in the dark overnight before imaging.

### RNA FISH and RNA FISH combined with IF

RNA FISH was performed as described previously^89^ with little modification. Cells were grown on 18-mm round coverslips with their respective adhesive matrix or feeders. The next day, cells were transferred to fresh 12-well plates after a series of washes with 1× PBS containing CaCl2 and MgCl2. Cells were then fixed in 4% PFA-PBS for 10 min at room temperature in the dark. After washing the coverslips with PBS to remove the remaining PFA, cells were permeabilized with cold 0.5% Triton ×100 in PBS for 10 min and washed twice with cold 0.2% Tween20 in PBS (PBST) for 5 min each. After this step, cells were either stored at −20 °C in 70% ethanol or directly subjected to RNA FISH or IF-FISH staining. When combining IF with RNA FISH, IF was performed as described above with the addition of RNAaseOUT (Invitrogen) and tRNA (Invitrogen) to the blocking buffer in all steps. After IF, cells were re-fixed in 4% PFA, followed by 2× SSC. Cells were kept at least for 1–2 h at 4 °C in 70% ethanol before starting FISH. RNA FISH for *XIST* was performed with DNA-modified RNA probes labeled with ChromaTide Alexa Fluro 488-5-DUTP (Thermo C11397), ChromaTide Alexa Fluro 594-5-DUTP (Thermo C11400) or Green 496 dUTP (Enzo ENZ-42831L) and AMINOALLYL DUTP CY(R)5 (Sigma 19475) using the Bioprime kit (Thermo 18095012) from a plasmid containing *XIST exon-1*^90^, and X-linked genes were obtained from BAC containing *HUWE1* (Bacpac resource center, RP11-975N19), *POLA1* (Bacpac resource center, RP11-11104L9), *ATRX* (Bacpac resource center, RP11-42M11), and *THOC2* (Bacpac resource center, RP11-121P4). When performing any FISH experiments, all the solutions contained 2 mM of the ribonucleoside-vanadyl complex. Before the hybridisation steps, coverslips were serially diluted with 70–100% ethanol. RNA probes were pre-hybridized at 37 °C at least for 1 h before use. RNA probes and samples were hybridized overnight at 37 °C. Afterwards samples were washed with 3 rounds of SSC solutions (1:1 formamide-2× SSC, 2× SSC and 1× SSC) at 42 °C by incubating 5–15 min each. Finally, the coverslips were mounted on the glass slides using Prolong Gold Antifade Mountant (Thermo P36930). Mounted coverslips were kept at room temperature in the dark overnight before imaging.

### Microscopy

Imaging of WT naive hESCs, EXMCs, and TSCs was done with a Zeiss Axioimager A1 upright microscope with an AxioCam MRc5 camera, Nikon TiA1R confocal microscope and processed in ImageJ. FISH and IF images were taken using a Nikon TiA1R confocal microscope. Bright field images were taken using a Nikon Eclipse Ti2 microscope and analyzed using ImageJ software.

Imaging of XIST iKD clone experiments was done on DMI-6000 inverted microscope with a motorized stage (Leica), equipped with a CCD Camera HQ2 (Roper Scientifics) and an HCX PL APO 64–100× oil objective (numerical aperture, 1.4, Leica) using the Metamorph software (version 7.04, Roper Scientifics). Brightfield images were taken using a Leica DM IL LED microscope equipped with a C4742-98-24ERG digital camera (Hamamatsu).

#### RNA FISH imaging

40 optical z-sections were acquired at 0.3 µm at different wavelengths depending on the modified-dUTPs: DAPI (360 nm, 470 nm), FITC (470 nm, 525 nm), Cy3 (550 nm, 570 nm), and Cy5 (647 nm, 668 nm). Stacks were processed using ImageJ and are represented as a 2D stacked maximum projection.

#### Super-resolution microscopy

3D-structured illumination microscopy (3D-SIM) was performed on an inverted Zeiss Elyra 7 (with lattice SIM module for structured illumination) microscope equipped with two PCO.edge 4.2 CLHS sCMOS cameras in combination with a 63× oil objective (NA 1.4). Alexa Fluor 555 was excited with a 561 nm green diode with 500 mW laser power and emission was collected with a filter allowing 570–620 nm emission detection. DAPI was excited with a 405 nm diode with 50 mW laser power and emission was collected with a filter allowing 420–480 nm emission detection. Raw images were computationally reconstructed using ZEN black (software version, Carl Zeiss Microscopy GmbH) and subsequently imported into ImageJ/Fiji or IMARIS software.

#### Super-resolution microscopy image analysis

3D-SIM image stacks were converted from.czi to.ims format using Imaris Converter 10.2.0 (Oxford Instruments) and analyzed in Imaris 10.2.0 using the Cells tool for segmentation. Nuclei were segmented based on the DAPI channel, while *XIST* foci were detected from the *XIST* channel using the vesicle detection pipeline within the Cells tool.

Nuclear segmentation was performed using an intensity threshold of 20 and a minimum object diameter of 10 μm. *XIST* foci were identified using the spot detection feature, with a minimum estimated diameter of 0.15 μm and a quality threshold above 26.7. All segmentation parameters, including intensity thresholds and size filters, were kept consistent across all cell types (naive, EXMCs, and TSCs) to ensure comparability. Segmentation results and associated statistics were exported as.csv files for downstream analysis.

Three-dimensional renderings and video animations of the confocal stacks were generated in Imaris to visualize the spatial distribution of *XIST* foci under different experimental conditions.

#### Super-resolution microscopy data analysis

To quantify the spatial distribution and clustering of *XIST* foci, a custom Python script was used to compute nearest neighbor distances (NNDs). XYZ coordinates of detected *XIST* foci were extracted from the exported.csv files (Vesicles_Type_A_Position.csv). Foci were grouped by image and cell ID, and a k-dimensional tree (KD-tree) was constructed for each group to efficiently compute Euclidean distances.

For each focus, the average distance to its five nearest neighbors (excluding itself) (NND_5) was calculated. Additionally, the number of *XIST* foci per nucleus was extracted from Cell_Number_Of_Vesicles_VesicleType = Vesicles_Type_A.csv using the same grouping strategy.

All datasets were merged into a single.xls file using image name and cell ID as keys. The merged data were further processed and visualized using custom R scripts to perform statistical analyses and generate plots.

### RNA extraction and RT-qPCR

RNA extraction was performed referring to instructions and reagents from RNeasy® Mini Kit (QIAGEN, 74106). Briefly, cell pellets were disrupted in RLT buffer supplemented with β-mercaptoethanol and stored at −80 °C until use. After adding 1 volume of 70% ethanol, total RNAs were purified on RNeasy spin columns according to the Mini Kit extraction protocol and finally eluted in RNAse-free water. Quantification of the extracted RNAs was done using the Nanodrop 2000. The DNase step was performed on 200 ng of RNA for 30 min at 37 °C using TURBO™ DNase (Thermo Fisher Scientific). Then RNAs were reverse transcribed using the Superscript IV kit (Thermo Fisher Scientific) following the manufacturer’s recommendation. cDNAs were diluted 5 times in water, and gene expression level was assessed by real-time quantitative PCR using the Power SYBR Green Master Mix (Thermo Fisher Scientific) and ViiA-7 Real-Time PCR system (Applied Biosystems). Primers can be found in the resource table. Transcript RNA levels were normalized against *GAPDH* reference gene following the 2−ΔCt method.

### Flow cytometry

hESCs were dissociated using Accutase (Sigma-Aldrich, A6964-100ML) into single cells by incubating for 5 min at 37 °C. Before proceeding with the antibody staining, cells were washed twice with FACS buffer containing 1% BSA in PBS (Gibco, 10010-015). Fluorophore-conjugated antibodies were diluted at a ratio of 1:50, which is 1 μL of antibody in 50 μL of FACS buffer for around 50,000 to 100,000 cells, and incubated at 4 °C in the dark for at least for 30 min. Cells were washed again with FACS buffer, passed through a 40 μM cell strainer (Corning, 352352340), analyzed/sorted using a BD influx or Beckman cytoflex SRT. Single-stained controls were used for compensation and setting up the precise and stringent gate in the flow cytometer. Analysis was done using the FlowJo software.

### Bulk RNA-seq

#### Cell preparation

Cells were harvested, pelleted and stored either in Trizol Reagent (Thermo, 15596026) or TRIreagents (Sigma, T9424). RNA extraction was done using Phenol-chloroform, isopropanol, and precipitated using 70% ethanol. RNA was treated with Dnase I (Roche) to remove DNA contamination. RNA clean up was done either with 70% ethanol precipitation or with the RNeasy columns protocol (Qiagen, 74104).

#### Library preparation

Libraries were generated using the Illumina Stranded Total RNA Prep Ligation with Ribo-Zero Plus kit (Illumina #20040525) according to the manufacturer’s recommendation and sequenced either on a NovaSeq 6000 instrument (ICGex—NGS platform, Paris, France) or on a NextSeq 2000 instrument to generate 2 × 100 paired-end reads.

#### Bulk RNA-seq analysis

Raw sequence reads were quality-checked using the FastQC software and cutAdapt^91^ was used to trim adapters. Reads were aligned to the human GRCh38 reference genome using STAR aligner^92^ and deduplicated using picard. Heterozygous SNPs were found by similarly aligning H9 whole genome sequencing data from GSM1227088^93^ and processing it with bcftools mpileup^94^. Expression in our bulk RNA-seq at each SNP was then determined using SNPsplit^95^ and quantified using bedtools genomecov^96^. Further analysis was performed in R. X-to-autosome ratios were calculated using the mean gene expression from each allele. Only SNPs with 30 or more read depth were included, as has been used previously^97^. Allelic ratios were calculated by determining which allele was more expressed, and dividing the least expressed allele by the most expressed allele. We limited our analyses to genes known to be subject to XCI in adults^72^, as genes known to escape from XCI were biallelic in all cell types. When multiple SNPs were available for a gene, the median allelic ratio was used to avoid the outliers, which are mentioned on the figures and figure legends. The detailed code of these analyses has been updated on GitHub.

### CUT&RUN

CUT&RUN was performed as previously described in ref. ^98^. Briefly, 0.3 million cells per replicate were bound to 20 µL concanavalin A-coated beads (Bangs Laboratories) in binding buffer (20 mM HEPES, 10 mM KCl, 1 mM CaCl_2_, and 1 mM MnCl_2_). The beads were washed and resuspended in Dig-wash buffer (20 mM HEPES, 150 mM NaCl, 0.5 mM spermidine, 0.05% digitonin). The primary antibodies (1:50) were added to the bead slurry and rotated at RT for 1 h. The antibodies list can be found in the resource table. The beads were washed by Dig-wash buffer and pA-Mnase fusion protein (1:400, produced by the Institut Curie Recombinant Protein Platform, 0.785 mg/mL) was added and rotated at RT for 15 min. After two washes, the beads were resuspended in 150 µL Dig-wash buffer, and the MNase was activated with 2 mM CaCl_2_ and incubated for 30 min at 0 °C. MNase activity was terminated with 150 µL 2 × STOP (200 mM NaCl, 20 mM EDTA, 4 mM EGTA, 50 µg/ml RNase A and 40 µg/ml glycogen). Cleaved DNA fragments were released by incubating for 20 min at 37 °C, followed by centrifugation for 5 min at 16,000 × *g* at 4 °C and collection of the supernatant from the beads on a magnetic rack. The DNA was purified by phenol:chloroform and libraries were prepared using the TruSeq ChIP Library Preparation Kit from Illumina following the manufacturer’s protocol, and sequenced on a NovaSeq 6000 instrument (ICGex—NGS platform, Paris, France) to generate 2 × 100 paired-end reads.

### CUT&Tag

#### CUT&Tag library preparation

CT27 hTSCs were grown on iMatrix-511 (Reprocell #NP892-011) in the medium described in ref. ^42^. Extraction of hTSC nuclei was performed as follows: cells were dissociated with Accumax (ThermoFisher Scientific #00-4666-56), washed once with PBS and resuspended in 500 µl nuclear extraction buffer consists of 20 mM HEPES-KOH pH 7.9, 10 mM KCl, 0.5 mM spermidine, 0.1% Triton ×-100, 20% glycerol and 1× protease inhibitor cocktail, PIC (Roche #11697498001). Samples were centrifuged at 600 × *g* for 3 min at 4 °C in a swing rotor centrifuge, and the supernatant was discarded. The extracted nuclei were fixed with 1 ml 0.2% formaldehyde and 1× PIC in PBS for 5 min at room temperature. The fixation was stopped by adding 30 µl 2.5 M glycine, and samples were incubated for 5 min on ice. Nuclei were then centrifuged at 600 × *g* for 3 min at 4 °C in a swing rotor centrifuge to remove the fixation solution. Finally, nuclei were resuspended in 100 µl Wash buffer (20 mM HEPES-NaOH pH 7.5, 150 mM NaCl, 0.5 mM spermidine and 1× PIC) supplemented with 0.1% BSA and 10% DMSO, and frozen slowly at −80 °C. We performed CUT&Tag as described^99^ with the following antibodies (anti-H3K27me3, CST, cat# 9733). Nuclei prepared from dissociated CT27 TSCs were immobilized to BioMag Plus Concanavalin-A-conjugated magnetic beads (ConA beads, Polysciences). Primary antibody was incubated with immobilized nuclei at 4 °C overnight. Followed, secondary antibody (Guinea Pig anti-Rabbit IgG, Sigma, cat# SAB3700889) was applied and incubated at room temperature for 30 min. Home-made pA-Tn5 was assembled with adapters and used for tagmentation of the regions bound by the target of interest. NEBnext PCR master mix (NEB, M0541) was used for tagmented fragment amplification and library preparation. DNA libraries were prepared using the NEBNext Ultra DNA library Prep Kit (NEB, E7645S).

#### Chromatin profiling analysis

Additionally, to our CUT&RUN data, we analyzed CUT&RUN, CUT&Tag from our study and ChIP-seq^53^. Reads were trimmed using trim_galore (0.6.5) (https://github.com/FelixKrueger/TrimGalore/) with a minimum length of 50 bp and were then mapped to the human genome (hg38) using bowtie2 (2.4.4)^100^ with the following parameters: –local –very-sensitive –no-unal –no-mixed –no-discordant –phred33 -L 10 -X 700. Duplicated reads were removed using MarkDuplicates from picard (2.23.5) (http://broadinstitute.github.io/picard/), and aligned reads were filtered for a minimum mapping quality of 10 using samtools (1.13)^101^. In all naive hESCs and capacitated datasets, reads originating from mouse embryonic fibroblasts (MEFs) were discarded using XenofilteR package from R (4.1.1)^99^. To create the correlation heatmap, mean read depth per 10 kb windows of the X chromosome were obtained using mosdepth (0.2.3)^102^ and then processed on R (4.3.2) to be normalized by library size and used to compute Spearman’s rank correlation coefficient and for the 500 most variable regions for each histone mark. To create all the other figures, aligned reads corresponding to replicates were merged and downsampled to the smallest library’s read count (26,5 M) using samtools (1.13)^101^. BigWig track files displaying the log2 of histone mark enrichment ratio over IgG were generated using bigwigCompare from deeptools (3.5.0)^103^ with the –operation log2 parameter, on histone mark and IgG BigWig files obtained with bamCoverage from deeptools (3.5.0)^103^ using the following parameters: –normalizeUsing BPM –binSize 20 –smoothLength 40. We used samtools idxstats (1.13)^101^ to obtain the total number of reads mapping to each chromosome. We calculated occupancy percentages by determining binding sites by peak calling using macs2 (2.2.7.1)^104^ with the following parameters: -f BAMPE -g 2913022398 –keep-dup “all” –broad –broad-cutoff 0.1 –nolambda, then merging peaks less than 10 kb apart using bedtools (2.30.0)^96^ and finally computing in R (4.3.2) the cumulative width of all peaks over the chromosome size. To analyze the annotations covered by the histone marks, we used bulk RNA-seq data of naive hESCs^26^, EXMCs and TSCs (this study) and the cDNA and ncRNA (GRCh38) sequences downloaded from ENSEMBL (www.ensembl.org) to select the most expressed isoform for each X-linked gene in each cell type using kallisto (0.46.2)^105^, and constructed an annotation file for each cell type from these results. Gene bodies were defined as the sequences between the Transcription Start Site (TSS) and Transcription End Site (TES) given in kallisto outputs, promoters as the 1 kb region preceding the TSS, the “upstream” regions as the 4 kb regions preceding promoters, predicted enhancers were downloaded from the FANTOM5 consortium collection hg38_v9^106^, and the remaining was defined as intergenic. An annotation was considered covered by a histone mark if 75% of the annotation sequence overlapped with a peak.

### MeD-seq

MeD-seq assays were essentially performed as previously described in ref. ^70^. Briefly, DNA from naive hESCs, EXMCs, and TSCs were extracted using the column DNeasy kit (Qiagen, 69504) following the manufacturer’s recommendations. Then, 10 μl genomic DNA (input 90 ng) from naive hESCs, EXMCs and TSCs were digested with LpnPI (New England Biolabs) generating 32 bp fragments around the fully methylated recognition site containing a CpG. These short DNA fragments were further processed using the ThruPlex DNA–seq 96D kit (Rubicon Genomics Ann Arbor). Stem-loop adapters were blunt-end ligated to repair input DNA and amplified to include dual indexed barcodes using a high-fidelity polymerase to generate an indexed Illumina NGS library. The amplified end product was purified on a Pippin HT system with 3% agarose gel cassettes (Sage Science). Multiplexed samples were sequenced on Illumina NextSeq2000 systems for paired-end read of 50 bp according to the manufacturer’s instructions. Dual indexed samples were de-multiplexed using bcl2fastq software (Illumina).

#### MeD-seq analysis

Reads were trimmed using trim_galore (0.6.5) (https://github.com/FelixKrueger/TrimGalore/) and were then mapped to the human genome (hg38) and the mouse genome (mm10) using bowtie2 (2.4.4)^100^ with the following parameters: –local –very-sensitive –no-unal –phred33 -L 10. BAM files were filtered to discard reads with mapping quality lower than 10 using samtools (1.13)^101^ and reads originating from mouse embryonic fibroblasts using XenofilteR package from R (4.1.1)^99^. BigWig files were generated using bamCoverage from deeptools (3.5.0)^103^ with the following parameters: normalizeUsing = “None”, binSize = 20, smoothLength = 40. CpG-island annotations were downloaded from ENSEMBL. Coverage scores were then computed in R (4.3.2) for each CpG located on the X chromosome, as log2 (x + 1), x being the number of reads per bin. Coverage scores were finally summed across the entire X chromosome or within specific genomic features. Gene bodies were defined as the sequences between the TSS and TES based on Ensembl annotation release 90 for GRCh38.p10 (www.ensembl.org), TSS regions as ± 1 kb around the TSS, and predicted enhancers were downloaded from the FANTOM5 consortium collection (hg38_v9)^106^.

### scRNA-seq

Cells were dissociated with Accutase (Stemcell Technologies, 07920) for 6 min (for naive hESCs day 0) or with TrypLE for 15 min (for day 4 and day 8 of differentiation) at 37 °C. Dissociation was stopped by adding DMEM and cells were pelleted and resuspended in PXGL or ASECRiAV. Feeder depletion was performed by placing cell suspensions on a gelatin pre-coated plate for 35–45 min. Cell counts were calculated using LunaFL (Logos Biosystems) and Propidium iodide stain (Logos, F23001). At least 100,000 cells were used for further steps. Samples were fixed and permeabilized using Chromium NEXT GEM Single Cell Fixed RNA Sample (10× Genomics, #1000414). Briefly, at each time point, cell pellets were resuspended in PFA and permeabilization reagents for 10 h at 4 °C. Suspensions were centrifuged and resuspended in quenching buffer, enhancer reagent, and glycerol and kept at −80 °C before further manipulation. Probes were hybridized, to add barcodes specific to each sample. Samples from each day were pooled together to obtain 20,000 cells per sample, unbound probes were then washed off. Gel beads in emulsion (GEM) were then generated to contain a unique barcoded gel bead, one cell and reaction enzyme and reagents in each droplet (Chromium, PN 1000475). Ligation, extension, clean up and library construction were then conducted according to the manufacturer’s recommendation. Pooled libraries were then sequenced on an Illumina Nextseq 2000 sequencing platform using a P3 100 cycles kit to a minimum sequencing depth of 10000 reads per cell using reads lengths of 28 bp read 1, 10 bp double indexes and 90 bp read 2.

#### Single-cell RNA-seq analysis

Single cell RNA-seq analysis was done at multiple time points in WT or XIST iKD cells as shown in Fig. 4A and as explained in ref. ^44^ with modifications. Raw sequence reads were quality-checked using the FastQC software. The CellRanger version 7.0.1 was used to process, align and summarize unique molecular identifier counts against the 10× Genomics pre-built human GRCh38 reference genome dataset (2020-A, July 7, 2020). Downstream analyses were performed in R using Seurat (v4.0.1)^107^. Cell with good sequencing reads were retained by adjusting the number of counts per cell (nCount_RNA) and the number of mapped genes per cell (nFeature_RNA) to only keep cells that were mostly mapped to the human GRCh38 (hg38) genome (nCount _RNA > 2200, nFeature_RNA > 1500). Cells with more than 25% of mitochondrial counts were filtered out. After quality control, we obtained data for 64479 cells with 28091 cells on day 0, 26650 cells on day 4 and 9738 cells on day 8. The count matrix was normalized with Seurat global-scaling normalization method “LogNormalize” that normalizes the feature expression measurements for each cell by the total expression, multiplies this by a 10,000-scale factor, and log-transforms the result. Cells were annotated based on gene expression. Differential expression testing was performed with the FindMarkers function in Seurat based on the non-parametric Wilcoxon rank sum test applying the logFC threshold of averaged log_2_ FC > 0.25. A graph-based cell clustering approach was used to cluster cells with the FindClusters function in Seurat.

Allelic gene expression was calculated using the scLinaX package for R^54^. Heterozygous SNPs were first identified using bcftools, and SNPs were filtered to only include those with >20 total reads, and >5 reads from each SNP across all cell types and time points. Vartrix was used to obtain SNP depth per allele for each cell and ANNOVAR^108^ was used to annotate and filter SNPs with less than 0.01 minor allele frequency in the gnomAD v4.1 database^109^. The allelic data was then processed further in R using the scLinaX pipeline. We used the default XCI status annotations from^71^. scLinaX first identifies the active X chromosome copy in each cell, then pools cells sharing the same active allele into pseudo‑bulk data sets, thereby improving detection of allele‑specific expression and escape from XCI.

### Quantification and statistical analysis

Statistical tests and data processing were performed in R (v4.0.3). All statistical methods and test values used can be found in the relevant result section, figures, and figure legends. In bulk RNA-seq, CUT&RUN, and Med-seq experiments, two replicates were used. In scRNA-seq experiments, one replicate was included per time point, and the number of cells per condition is indicated in source data file. During the processing step of the scRNA-seq cells were excluded with nCounts RNA < 2200, nFeature RNA < 1500, and >25% of mitochondrial reads. Differential gene expression analysis was performed using the Seurat function FindMarkers using cutoffs of adjusted *p*-value < 0.05 and log2 fold change > 0.5.

All the experiments were repeated at least two or three times, and with cells with different genetic backgrounds, with exceptions. Sequencing experiments were done once, with or without replicates. Aside from the scRNA-seq experiment, all other *XIST* iKD experiments, including IF, FISH, differentiation, flow cytometry, and brightfield imaging, were performed using three independent XIST iKD clones, with the exception of Annexin V flow cytometry data, where 2 clones were used. For the *XIST* iKD, three independent clones were used for all experiments related to Figs. 4B–E and 5F, G. Experiments were repeated three times for **1C–H and 3A–F**, once for **1B, 2A–H, 4F–L, and 5F-5G**, and two times for all other figures. Experiments were not blinded. No data were excluded with the exception of cell filtering described in the scRNA-seq analysis as well as in this section above. Specifically, a logistic regression test was used for Figs. 1E, G, 3B, D, F, K, M, and S5I; a chi-squared test for Figs. 2I, 4E, S6C, S6H, S6I and S6C and G; a *t*-test for Figs. 1B, S1R, 3I, S4C, D, 5 B, and S6D; and a Kolmogorov–Smirnov for 5A; and a Wilcoxon rank-sum test for Figs. S1I, J, S4J, K, and S4D, E. **For the binomial logistics regression test**, the three independent experiments were included as a categorical covariate in the model. For all statistical tests, *p* < 0.05 was considered significant, unless specifically mentioned.

In RNA FISH experiments, ~25% naive hPSC and ~5–10% EXMC and TSC nuclei had no detectable XIST FISH signal. Analyses focussed on nuclei with detectable FISH signals.

Post-scLinaX statistical comparisons using only genes informative in both cell types being compared are shown in the Supplementary Data 4.

### Reporting summary

Further information on research design is available in the Nature Portfolio Reporting Summary linked to this article.

## Supplementary information

Supplementary Information

Reporting Summary

Description of Additional Supplementary Files

Supplementary Data 1

Supplementary Data 2

Supplementary Data 3

Supplementary Data 4

Supplementary Movie 1

Supplementary Movie 2

Supplementary Movie 3

Peer Review file

## Source data

Source data

## Data Availability

Raw and processed sequencing data (scRNA-seq, bulk RNA-seq, CUT&RUN, and MeD-seq) have been submitted to the NCBI GEO under accession number GSE261711. This paper analyzes eXISTing, publicly available data. The accession numbers for the datasets are listed in the key resources table. Source data are provided with this paper.

## Code availability

All analysis code is available at: https://github.com/pasquelab/Human-XIST-XCI_AP. Any additional information required to reanalyze the data reported in this paper is available from the lead contact upon request.
