# Supplementary material for: XIST drives X-chromosome inactivation and safeguards female extraembryonic cells in humans
Source: Nat Commun. 2026 Oct 22;17:10738. doi: 10.1038/s41467-026-78394-z (PMC13691081; doi:10.1038/s41467-026-78394-z)
Supplement: Supplementary file 1 [file 41467_2026_78394_MOESM11_ESM.pdf]

nature portfolio

Peer Review File

XIST Drives X-Chromosome Inactivation and Safeguards Female Extraembryonic Cells in Humans

Corresponding Author: Professor Vincent Pasque

This file contains all reviewer reports in order by version, followed by all author rebuttals in order by version.

Version 0:

Reviewer comments:

Reviewer #2

(Remarks to the Author)
I am re-reviewing the manuscript by Panda & Carillo on the role of XIST in human extraembryonic differentiation and establishment of XCI. I am pleased to see the many changes made in the revision and appreciate the thorough replies from the authors to the many points raised by myself and the two other reviewers. I am still enthusiastic about this work that provides new evidence for the central role of XIST in human XCI and the importance of XCI for normal embryonic development. There is the caveat that the work is done in a cell model which may not fully generalise to in vivo human development, yet this is largely the most accessible way of studying these phenomena in humans as of now.

With the major methodological criticism I had in my earlier review addressed I have only a few remaining remarks.

I appreciate the authors following the reviewers' advice and replacing the bulk RNA-seq analyses in Figure 1 with scRNA-seq based assessments of XCI using scLinaX. I do wonder, though, whether scLinaX works quite as intended in determining the correlation between the X-linked SNPs when a large fraction of the input data is from cells with no XCI, but testing for this is outside the scope of this manuscript. And given that the results largely support the other lines of evidence presented in the manuscript, I am thinking the potential impact is perhaps less substantial.

However, a complementary metric the authors could consider showing to support the findings of the establishment of XCI along the differentiation path is the fraction of biallelic expression in the X chromosome in individual cells at the three time points examined. While biallelic expression in 10x data at individual cells is likely very limited, I would expect the full lack of XCI (in D0) to be reflected as clear biallelically expressed sites in some of the highly expressed X-linked genes.

Additionally, the authors could show in the supplemental data a similar figure to Fig1B but with established escapees to show how the XCI dynamics affects escape. Now these different gene classes in the X chromosome and autosomal genes are only shown for the bulk RNA-seq data.

Ideally the authors could bridge the single cell findings with the RNA FISH results for the three genes by showing examples of how these same genes behave in the single cell data, yet this is perhaps not possible, with perhaps the exception of XIST, given the sparsity of the allelic information in human single cells.

Minor comments:

I am not sure if the authors have provided the full results for the analyses presented in Fig1B. Supplementary Table 6 provides the full scLinaX output for the results presented in Fig3I but it is not clear if the data in Fig1B is just a summary of these results or an independent analysis. Please clarify.

Please indicate in the figure legend what the red line in Fig1B refers to.

I appreciate the further details provided on the statistical analyses for Fig1D. These details on the statistical approach could also be elaborated in the figure legend as it is not specified that individual cells are used as observations when testing for the association. Further, please clarify how the three replicates were accounted for in the analyses.

Generally, I propose the authors go through the figure legends and/or methods to ensure all necessary information required to replicate the statistical analyses are provided. As another example, I find it unclear which data is shown in Fig5A, e.g., are the data points individual cells, and how the comparison between X-chromosomal and autosomal expression is conducted.

With regards to the code, I propose the authors make sure the updates to Fig1 are also reflected in the code provided. In the ideal case, the repository and available results would be organized to allow one to replicate the key figures of the manuscript.

Reviewer #3

(Remarks to the Author)
The authors have made substantial efforts to address my previous comments, and the revised manuscript has improved considerably. Overall, I am now inclined to support publication of the manuscript.

However, I would like to raise one general concern regarding the authors' responses. In several instances, the authors have addressed the issues raised in my previous review primarily by acknowledging them as limitations in the revised manuscript. While transparent discussion of a study's limitations is appropriate and appreciated, simply acknowledging a limitation should not, by itself, be considered a sufficient response when the underlying concern relates to the strength of the evidence or the validity of the interpretation.

Where additional experiments or analyses are not feasible, the corresponding conclusions should be appropriately tempered and remain strictly within the scope of the available data. In particular, where sufficient in vivo evidence is lacking, this should be explicitly acknowledged, and the conclusions and broader claims should be moderated accordingly. I remain concerned that the evidentiary support for several of the authors' interpretations remains insufficient or ambiguous.
The analysis of TSCs derived from primary human cytotrophoblasts is a valuable addition and strengthens the manuscript. However, these cells remain ex vivo cultured derivatives of placental cytotrophoblasts obtained at a substantially later developmental stage and therefore do not constitute direct in vivo evidence for the Xi chromatin configuration during early post-implantation development.

I appreciate the practical, ethical, and technical difficulties associated with obtaining the relevant human embryonic material. I also agree that current stem cell-based systems represent the best available experimental models and that additional in vivo validation is presently not feasible. Nevertheless, these limitations do not resolve the underlying evidentiary issue, nor do they justify conclusions that extend beyond the experimental system examined. The manuscript should therefore clearly distinguish between findings derived from stem cell-based or ex vivo TSC models and conclusions regarding the chromatin state of extraembryonic cells in vivo during early post-implantation development. Any statements implying that this chromatin configuration has been demonstrated in vivo at the relevant developmental stage should be appropriately revised or moderated to reflect the available evidence.

Provided that these points are appropriately addressed, I would have no further major concerns regarding publication.

Open Access This Peer Review File is licensed under a Creative Commons Attribution 4.0 International License, which permits use, sharing, adaptation, distribution and reproduction in any medium or format, as long as you give appropriate credit to the original author(s) and the source, provide a link to the Creative Commons license, and indicate if changes were made.
In cases where reviewers are anonymous, credit should be given to 'Anonymous Referee' and the source.
The images or other third party material in this Peer Review File are included in the article's Creative Commons license, unless indicated otherwise in a credit line to the material. If material is not included in the article's Creative Commons license and your intended use is not permitted by statutory regulation or exceeds the permitted use, you will need to obtain permission directly from the copyright holder.
To view a copy of this license, visit https://creativecommons.org/licenses/by/4.0/

General Response to the Reviewers (Revision 2)

We thank the reviewers for their careful re-evaluation of our manuscript and for their constructive and thoughtful comments. We are particularly grateful for their recognition of the substantial improvements made during the revision process and for their continued enthusiasm for the study.

In this revision, we have carefully considered all remaining concerns and have made further changes to improve the clarity and reproducibility of the manuscript. In particular, we have clarified the statistical approaches and the nature of the observations underlying several of the analyses, including the treatment of independent experimental replicates and the calculation and statistical comparison of X-chromosome-to-autosome expression ratios. We have also further clarified the relationship between the scLinaX analyses presented in the main and supplementary figures and updated the accompanying code repository to reflect the revised analyses and facilitate reproducibility of key figures.

Importantly, we have also carefully revisited the interpretation and scope of our conclusions. We agree that findings obtained using human stem cell-based models and ex vivo TSCs should not be presented as direct evidence of the corresponding processes in vivo during early human development. We have therefore moderated relevant statements throughout the manuscript and clearly distinguished experimental observations made in our stem cell-based and ex vivo models from broader implications for human embryonic development. While these models provide experimentally accessible systems for investigating otherwise difficult-to-study aspects of early human development, we acknowledge that direct in vivo validation remains an important limitation of the current study.

Overall, we believe that these revisions have further strengthened the manuscript by improving the analyses, the reproducibility of the work, and the alignment between our conclusions and the evidence provided by our experimental systems. We sincerely thank the reviewers for their constructive feedback, which has helped us improve the manuscript substantially.

Point-by-point response to each Reviewer's Comments:

Reviewer's questions are indicated in black. Our responses are indicated in blue.

Response to Reviewer #2

I am re-reviewing the manuscript by Panda & Carillo on the role of XIST in human extraembryonic differentiation and establishment of XCI. I am pleased to see the many changes made in the revision and appreciate the thorough replies from the authors to the many points raised by myself and the two other reviewers. I am still enthusiastic about this work that provides new evidence for the central role of XIST in human XCI and the importance of XCI for normal embryonic development. There is the caveat that the work is done in a cell model which may not fully generalise to in vivo human development, yet this is largely the most accessible way of studying these phenomena in humans as of now.

Response: We thank Reviewer #2 for their appreciation of our work and for the positive comments, recognising the scientific rigour and experimental practices, and enthusiasm towards the novelty of the work. We agree that our study is based on in vitro human stem cell-derived models and therefore may not fully recapitulate the complexity of in vivo human development. We have accordingly restricted our conclusions and claims to the experimental systems examined in this study and have refrained from making broad conclusions regarding XCI or extraembryonic development in human embryos. Nevertheless, these

emerging human stem cell-based models provide an important and experimentally accessible system for investigating the molecular mechanisms underlying early human development and epigenetic regulation, including processes that are currently challenging to study directly in human embryos. The revised manuscript contains a clear limitations section.

With the major methodological criticism I had in my earlier review addressed I have only a few remaining remarks.

Response: We are pleased the previous major methodological criticism is deemed addressed. We address the remaining remarks below.

I appreciate the authors following the reviewers' advice and replacing the bulk RNA-seq analyses in Figure 1 with scRNA-seq based assessments of XCI using scLinaX. I do wonder, though, whether scLinaX works quite as intended in determining the correlation between the X-linked SNPs when a large fraction of the input data is from cells with no XCI, but testing for this is outside the scope of this manuscript. And given that the results largely support the other lines of evidence presented in the manuscript, I am thinking the potential impact is perhaps less substantial.

Response: We thank the reviewer for raising this point regarding the application of scLinaX in a dataset containing cells with different XCI states. We agree that this is an important methodological consideration and appreciate the reviewer's perspective on its potential impact. As noted by the reviewer, the scLinaX-based assessment is consistent with the independent lines of evidence presented throughout the manuscript, such as bulk RNA-seq, FISH, 3D-SIM, IF-FISH, and IF. We therefore interpret the scLinaX results in the context of these complementary analyses rather than as a standalone measure of XCI status. We agree that a systematic evaluation of the performance of scLinaX under these specific conditions is beyond the scope of the present study.

However, a complementary metric the authors could consider showing to support the findings of the establishment of XCI along the differentiation path is the fraction of biallelic expression in the X chromosome in individual cells at the three time points examined. While biallelic expression in 10x data at individual cells is likely very limited, I would expect the full lack of XCI (in D0) to be reflected as clear biallelically expressed sites in some of the highly expressed X-linked genes.

Response: We thank the reviewer for this constructive suggestion. We agree that single-cell quantification of biallelic X-linked expression would provide a useful complementary analysis. We were not able to complete a robust allele-specific analysis during the revision period. We have therefore added this point as a limitation and revised the text to avoid interpreting the timing of XCI establishment based solely on the current data. Our conclusion is supported by current data, but we agree that a dedicated allele-specific analysis, ideally using higher-coverage or complementary data, will be important to resolve biallelic expression dynamics more directly.

Additionally, the authors could show in the supplemental data a similar figure to Fig1B but with established escapees to show how the XCI dynamics affects escape. Now these different gene classes in the X chromosome and autosomal genes are only shown for the bulk RNA-seq data.

Response: We thank the reviewer for this suggestion. We have now added this panel as new Figure 1C and revised the manuscript accordingly.

Ideally the authors could bridge the single cell findings with the RNA FISH results for the three genes by showing examples of how these same genes behave in the single cell data,

yet this is perhaps not possible, with perhaps the exception of XIST, given the sparsity of the allelic information in human single cells.

Response: We appreciate the reviewer's suggestion. However, when we attempted to perform this analysis, we were unable to identify informative SNPs in the four X-linked genes used for FISH across all samples. Following our standard filtering criteria, the majority of these genes did not contain informative SNPs (data provided in the source data file). When we relaxed the filtering criteria, informative SNPs could be identified for some genes in individual samples; however, these SNPs were not consistently informative across all samples. Therefore, we were unable to perform a robust allelic analysis based on these loci.

Minor Points

I am not sure if the authors have provided the full results for the analyses presented in Fig1B. Supplementary Table 6 provides the full scLinaX output for the results presented in Fig3I but it is not clear if the data in Fig1B is just a summary of these results or an independent analysis. Please clarify.

Response: We thank the reviewer for pointing this out. Figure 1B does not represent an independent analysis; rather, it presents a summary of the scLinaX analysis shown in Figure 3I, with only three selected time points highlighted. The source data file and the supplementary data files contain the complete scLinaX output, including all data points underlying both Figure 1B and Figure 3I.

Please indicate in the figure legend what the red line in Fig1B refers to.

Response: We have now updated this in the figure legend. The dotted line indicates the silencing threshold (minor allele expression <0.1%).

I appreciate the further details provided on the statistical analyses for Fig1D. These details on the statistical approach could also be elaborated in the figure legend as it is not specified that individual cells are used as observations when testing for the association. Further, please clarify how the three replicates were accounted for in the analyses.

Response: We agree this is important, and we thank the reviewer for pointing this out. We have clarified the statistical analysis in the revised figure legend. For each independent experiment, individual cells were classified as showing monoallelic or biallelic expression, and these data were analyzed as grouped binomial counts in a logistic regression model. This is mentioned in the method section as well as in the figure legends. Cell type was included as the predictor of interest, with naïve cells as the reference group, and the three independent experiments were included as a categorical covariate to account for experiment-to-experiment variation. The technical triplicates within each experiment were pooled for the analysis. Thus, the statistical model accounts for the three independent experiments while testing for differences in the proportion of monoallelic versus biallelic cells between cell types. We have updated the figure legends accordingly. As we have mentioned earlier, all statistical tests were done as per the suggestion of a biostatistician from KU Leuven, Belgium.

Generally, I propose the authors go through the figure legends and/or methods to ensure all necessary information required to replicate the statistical analyses are provided. As another example, I find it unclear which data is shown in Fig5A, e.g., are the data points individual cells, and how the comparison between X-chromosomal and autosomal expression is conducted.

Response: We thank the reviewer for pointing out this lack of clarity. We have clarified the nature of the data points and the calculation of the X-chromosome-to-autosome expression

ratio in the revised Figure 5A legend. Each data point represents an individual scRNA-seq cell. For each cell, we calculated the mean expression across genes located on chromosome X and divided this by the mean expression across genes located on autosomes (chromosomes 1–22), resulting in a per-cell X:A expression ratio. Thus, the analysis compares the relative mean expression of X-chromosomal versus autosomal genes within each cell, rather than comparing individual X-linked and autosomal genes. Comparisons between experimental conditions were subsequently performed using a two-sided t-test on randomly downsampled cells (100 cells per condition), with Bonferroni correction for multiple comparisons. The majority of this information is in the figure legends, however due to 350 word limits some of the information may have been shifted to the statistics section available in the Methods.

With regards to the code, I propose the authors make sure the updates to Fig1 are also reflected in the code provided. In the ideal case, the repository and available results would be organized to allow one to replicate the key figures of the manuscript.

Response: We thank Reviewer #2 for this suggestion. We have updated the code repository to reflect the revised Figure 1 and its associated analyses. Specifically, the folder previously corresponding to Figure 1B has been renamed to Figure S1 (The bulk RNA-seq data analysis directory), and the scLinaX analysis used to generate the revised Figure 1B has been included in the dedicated scLinaX analysis directory. We have also clearly indicated in the relevant code and folder names that the Figure 1B panel was generated using this analysis. These updates are intended to improve the organization of the repository and facilitate replication of the key analyses and figures presented in the manuscript.

Response to Reviewer #3

The authors have made substantial efforts to address my previous comments, and the revised manuscript has improved considerably. Overall, I am now inclined to support publication of the manuscript.

Response: We sincerely thank the reviewer for recognizing and appreciating our efforts to address the previous comments and for their continued support and enthusiasm for our work and supporting publication.

However, I would like to raise one general concern regarding the authors' responses. In several instances, the authors have addressed the issues raised in my previous review primarily by acknowledging them as limitations in the revised manuscript. While transparent discussion of a study's limitations is appropriate and appreciated, simply acknowledging a limitation should not, by itself, be considered a sufficient response when the underlying concern relates to the strength of the evidence or the validity of the interpretation.

Response: We agree with Reviewer #3. We have carefully reviewed our manuscript and considered places where the strength of the evidence or the validity of the evidence may have been deemed too strong and toned down the statements accordingly. We thank the reviewer for raising this important general point. We agree that acknowledging a limitation alone is not sufficient when the available evidence does not fully support the strength of a particular interpretation. We have therefore carefully revisited the manuscript and further moderated statements that could be interpreted as extending our findings beyond the experimental systems examined.

Where additional experiments or analyses are not feasible, the corresponding conclusions should be appropriately tempered and remain strictly within the scope of the available data. In particular, where sufficient in vivo evidence is lacking, this should be explicitly acknowledged, and the conclusions and broader claims should be moderated accordingly. I

remain concerned that the evidentiary support for several of the authors' interpretations remains insufficient or ambiguous.

The analysis of TSCs derived from primary human cytotrophoblasts is a valuable addition and strengthens the manuscript. However, these cells remain ex vivo cultured derivatives of placental cytotrophoblasts obtained at a substantially later developmental stage and therefore do not constitute direct in vivo evidence for the Xi chromatin configuration during early post-implantation development.

Response: We agree that our stem cell-based embryo models and TSCs derived from primary human cytotrophoblasts provide important experimental evidence relevant to XCI and chromatin regulation in human extraembryonic lineages but do not constitute direct in vivo evidence for the chromatin state of extraembryonic cells during early post-implantation development. We have accordingly revised the relevant text throughout the manuscript to clearly distinguish observations made in stem cell-based and ex vivo models from conclusions regarding human development in vivo.

I appreciate the practical, ethical, and technical difficulties associated with obtaining the relevant human embryonic material. I also agree that current stem cell-based systems represent the best available experimental models and that additional in vivo validation is presently not feasible. Nevertheless, these limitations do not resolve the underlying evidentiary issue, nor do they justify conclusions that extend beyond the experimental system examined. The manuscript should therefore clearly distinguish between findings derived from stem cell-based or ex vivo TSC models and conclusions regarding the chromatin state of extraembryonic cells in vivo during early post-implantation development. Any statements implying that this chromatin configuration has been demonstrated in vivo at the relevant developmental stage should be appropriately revised or moderated to reflect the available evidence.

Provided that these points are appropriately addressed, I would have no further major concerns regarding publication.

Response: We have also clarified that the primary cytotrophoblast-derived TSC experiments provide an independent and complementary line of evidence supporting the conservation of the observed chromatin features in extraembryonic trophoblast cells but do not by themselves establish that the same chromatin configuration exists in vivo at the corresponding developmental stage.

Where direct in vivo validation is not currently feasible, we have restricted our conclusions to what is supported by the available experimental evidence and explicitly acknowledged the absence of direct in vivo evidence. We believe these revisions provide a more appropriate balance between the significance of our findings and the limitations inherent to the available human developmental models.
