# Supplementary material for: XIST drives X-chromosome inactivation and safeguards female extraembryonic cells in humans
Source: Nat Commun. 2026 Oct 22;17:10738. doi: 10.1038/s41467-026-78394-z (PMC13691081; doi:10.1038/s41467-026-78394-z)
Supplement: Supplementary file 2 [file 41467_2026_78394_MOESM1_ESM.pdf]

Supplementary Information

XIST Drives X-Chromosome Inactivation and Safeguards Female Extraembryonic Cells in Humans

Amitesh Panda[1,2,3,&,#], Léo Carrillo[4,#], Bradley Philip Balaton[1,2,3], Jeanne Brouillet[4], Solomon Nshemereirwe[1,2,3], Jarne Bonroy[1,2,3], Charbel Alfeghaly[4,5], Romina Facchinello[4,5], Sherif Khodeer[1,2,3], Nicolas Peredo[6,7], Ruben Boers[8], Gael Castel[4], Charlie London[4], Emmanuel Cazottes[4], Madeleine Moscatelli[4], Raissa Songwa Tchinda[1,2,3], Thi Xuan Ai Pham[1,2,3], San Kit To[1,2,3], Ryan Nicolaas Allsop[1,2,3], Yang Wang[9], Desislava Staneva[10], Peter J. Rugg-Gunn[9,10,11], Kathy K. Niakan[9,10,11], Joost Gribnau[8], Jean-François Ouimette[4,5,*], Claire Rougeulle[4,5,*] and Vincent Pasque[1,2,3*].

[1]KU Leuven, Department of Development and Regeneration, B-3000 Leuven, Belgium

[2]Leuven Institute for Single-cell Omics (LISCO), B-3000 Leuven, Belgium

[3]Leuven Cancer Institute (LKI), B-3000 Leuven, Belgium

[4]Université Paris Cité, CNRS, Epigenetics and Cell Fate, F-75013 Paris, France

[5]Institut Curie, PSL University, Sorbonne Université, Université Paris Cité, CNRS UMR3244, Dynamics of Genetic Information, 75005 Paris, France

[6]VIB BioImaging Core Leuven, VIB Technologies, Center for Neuroscience Leuven, Leuven, Belgium

[7]VIB BioImaging Core Leuven, Department of Neurosciences, KU Leuven, Leuven, Belgium

[8]Department of Developmental Biology, Erasmus University Medical Center, Rotterdam, The Netherlands

[9]Epigenetics Programme, Babraham Institute, Cambridge, UK

[10]Loke Center for Trophoblast Research, Department of Physiology, Development and Neuroscience, University of Cambridge, Cambridge, UK

[11]Cambridge Stem Cell Institute, University of Cambridge, Cambridge, UK

[&] Current address: Department of Genome Regulation, Max Planck Institute for Molecular Genetics, Berlin, Germany

[#] These authors contributed equally

* Corresponding and co-last authors:
J.F.O. (jean-francois.ouimette@curie.fr)
C.R. (claire.rougeulle@curie.fr)
V.P. (vincent.pasque@kuleuven.be)

Supplementary figures 1-9

Extended data figures 1-4

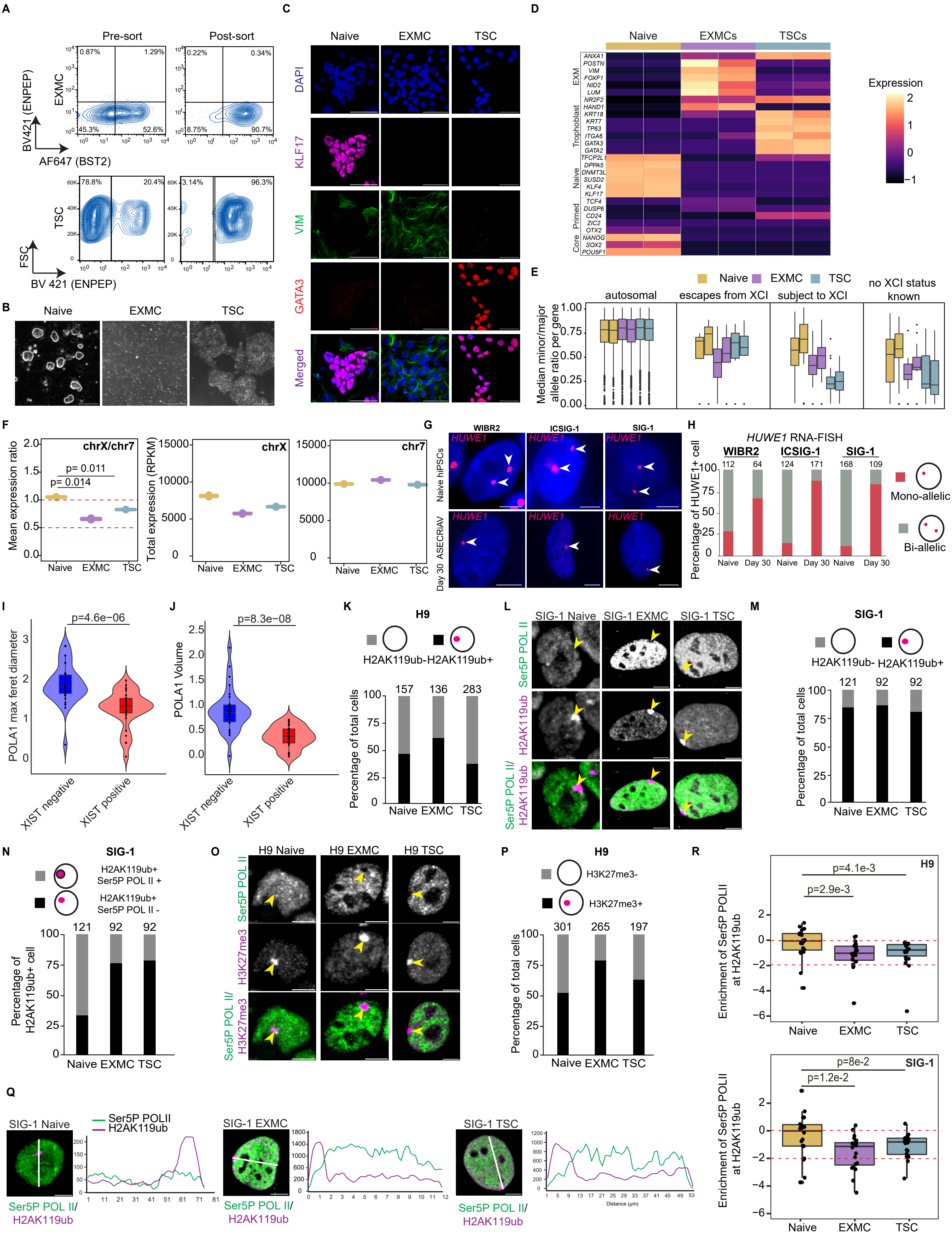
A
Pre-sort
Post-sort
EXMC
BV421 (ENPEP)
AF647 (BST2)
0.87%
1.29%
45.3%
52.6%
0.22%
0.34%
8.75%
90.7%
TSC
FSC
BV 421 (ENPEP)
78.8%
20.4%
3.14%
96.3%
B
Naive
EXMC
TSC
C
Naive
EXMC
TSC
DAPI
KLF17
VIM
GATA3
Merged
D
Naive
EXMCs
TSCs
Expression
EXM
ANXA1
POSTN
VIM
FOXF1
NID2
LUM
NR2F2
HAND1
KRT18
Trophoblast
KRT7
TP63
ITGA6
GATA3
GATA2
TFCP2L1
Naive
DPPA5
DNMT3L
SUSD2
KLF4
KLF17
TCF4
Primed
DUSP6
CD24
ZIC2
OTX2
Core
NANOG
SOX2
POU5F1
E
Naive
EXMC
TSC
autosomal
escapes from XCI
subject to XCI
no XCI status known
Median minor/major allele ratio per gene
F
Mean expression ratio
chrX/chr7
p= 0.011
p= 0.014
Naive
EXMC
TSC
Total expression (RPKM)
chrX
chr7
G
WIBR2
ICSIG-1
SIG-1
HUWE1
Naive hiPSCs
Day 30 ASECRiAV
H
HUWE1 RNA-FISH
WIBR2
ICSIG-1
SIG-1
Percentage of HUWE1+ cell
112
64
124
171
168
109
Naive
Day 30
Mono-allelic
Bi-allelic
I
POLA1 max feret diameter
p=4.6e-06
XIST negative
XIST positive
J
POLA1 Volume
p=8.3e-08
XIST negative
XIST positive
K
H9
H2AK119ub-
H2AK119ub+
Percentage of total cells
157
136
283
Naive
EXMC
TSC
L
SIG-1 Naive
SIG-1 EXMC
SIG-1 TSC
Ser5P POL II
H2AK119ub
Ser5P POL II/ H2AK119ub
M
SIG-1
H2AK119ub-
H2AK119ub+
Percentage of total cells
121
92
92
Naive
EXMC
TSC
N
SIG-1
H2AK119ub+ Ser5P POL II +
H2AK119ub+ Ser5P POL II -
Percentage of H2AK119ub+ cell
121
92
92
Naive
EXMC
TSC
O
H9 Naive
H9 EXMC
H9 TSC
Ser5P POL II
H3K27me3
Ser5P POL II/ H3K27me3
P
H9
H3K27me3-
H3K27me3+
Percentage of total cells
301
265
197
Naive
EXMC
TSC
R
H9
p=4.1e-3
p=2.9e-3
Enrichment of Ser5P POLII at H2AK119ub
Naive
EXMC
TSC
SIG-1
p=8e-2
p=1.2e-2
Enrichment of Ser5P POLII at H2AK119ub
Naive
EXMC
TSC
Q
SIG-1 Naive
Ser5P POLII
H2AK119ub
Ser5P POL II/ H2AK119ub
SIG-1 EXMC
Ser5P POL II/ H2AK119ub
SIG-1 TSC
Distance (μm)
Ser5P POL II/ H2AK119ub

SUPPLEMENTARY FIGURES

Figure S1: XCI is established in human extraembryonic cells, related to Figure 1.

A. Flow cytometry contour plots of pre-/post-sort BST2-positive/negative EXMCs (top) and ENPEP-positive/negative TSCs (bottom).

B. Bright-field images of H9 naive hESCs, EXMC, and TSC. Scale bar, 1000 μm.

C. Immunofluorescence of KLF17 (Magenta), GATA3 (Red), and VIM (Green) in H9 naive hESCs, EXMCs and TSCs. DAPI in in blue. Scale bar, 50 μm.

D. Heatmap of marker gene expression (EXM, trophoblast, naive, primed, and core pluripotency genes in H9 naive hESCs, EXMCs and TSCs). Per-gene Z-score is shown.

E. Median SNP ratio of least to most expressed allele on the X chromosome by published XCI status (5851 autosomal genes; 14 escaping XCI; 53 subject to XCI; 41 unknown).

F. Mean X-to-chromosome 7 expression ratio (left), total X chromosome (Middle) and total chromosome 7 (Right) expression in RPKM.

G. RNA FISH images *HUWE1* (Magenta) in three different genetic backgrounds of naive and day 30 trophoblast medium. Scale bar, 50 μm.

H. Proportion of biallelic and monoallelic *HUWE1* spots from G; number of counted nuclei above each bar.

I. Violin plots of POLA1 focus maximum feret diameter (in μm), *XIST*-positive versus *XIST*-negative alleles, and day 0 H9 naive hESCs (31 nuclei, 2 batches). Wilcoxon rank-sum test;p-value < 0.05 significant.

J. As in I, but for maximum volume (in μm^3).

K, M. Immunofluorescence H2AK119ub foci in H9 (K) and SIG-1 (M) naive hPSCs, EXMC, and TSC detected by IF. Nuclei counted above each bar.

L. Immunofluorescence of Ser5P POL II (Green) and H2AK119ub (Magenta) in SIG-1 naive hiPSCs, EXMCs and TSCs. Scale bar, 5 μm.

N. H2AK119ub foci with/without Ser5P Pol II overlap, SIG-1; nuclei counted above each bar.

O. IF of Ser5P Pol II (green) and H3K27me3 (magenta) in H9 cells. Scale bar, 5 um.

P. Quantification of H3K27me3 foci. Nuclei counted above each bar.

Q. Left: IF of Ser5P Pol II (green), H2AK119ub (magenta), SIG-1. Scale bar, 5 um. Right: Ser5P Pol II intensity across the H2AK119ub cloud.

R. Ser5P Pol II intensity at the H2AK119ub foci vs outside (panels 1L, S1L); 20 nuclei/type. t-test p-values shown.

For panels H, K, M, N, and P, the number of nuclei counted is indicated in the source data file. Source data are provided as a Source Data file. E, I and R, box plots show the median, 25th/75th percentiles, and whiskers to 1.5× interquartile range (IQR); points beyond whiskers are outliers. N = 32 genes.

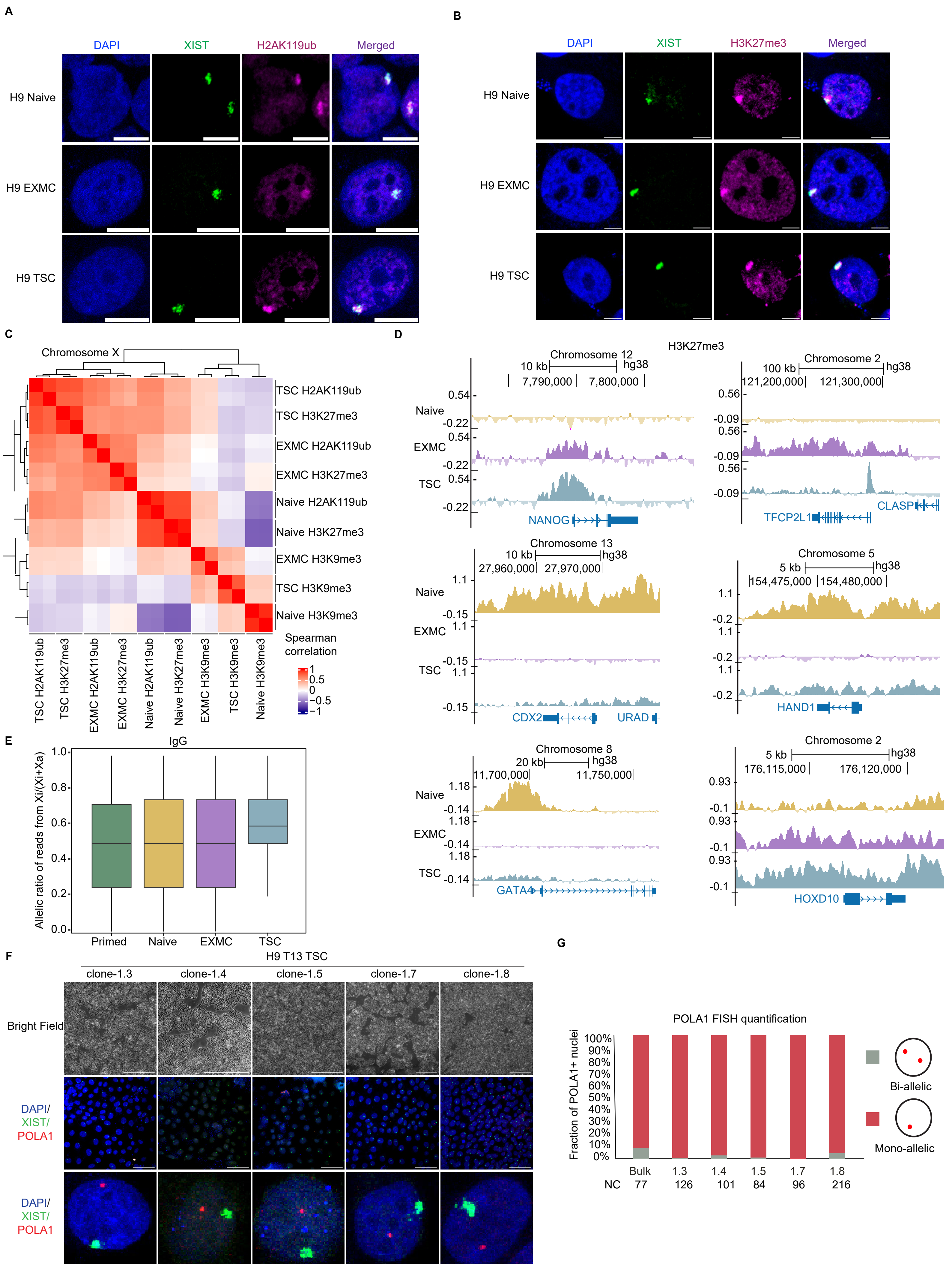

A
DAPI
XIST
H2AK119ub
Merged
H9 Naive
H9 EXMC
H9 TSC
B
DAPI
XIST
H3K27me3
Merged
H9 Naive
H9 EXMC
H9 TSC
C
Chromosome X
TSC H2AK119ub
TSC H3K27me3
EXMC H2AK119ub
EXMC H3K27me3
Naive H2AK119ub
Naive H3K27me3
EXMC H3K9me3
TSC H3K9me3
Naive H3K9me3
Spearman correlation
1
0.5
0
−0.5
−1
D
H3K27me3
Chromosome 12
10 kb
hg38
7,790,000
7,800,000
Naive
EXMC
TSC
0.54
-0.22
NANOG
Chromosome 2
100 kb
hg38
121,200,000
121,300,000
0.56
-0.09
TFCP2L1
CLASP1
Chromosome 13
10 kb
hg38
27,960,000
27,970,000
1.1
-0.15
CDX2
URAD
Chromosome 5
5 kb
hg38
154,475,000
154,480,000
1.1
-0.2
HAND1
Chromosome 8
20 kb
hg38
11,700,000
11,750,000
1.18
-0.14
GATA4
Chromosome 2
5 kb
hg38
176,115,000
176,120,000
0.93
-0.1
HOXD10
E
IgG
Allelic ratio of reads from Xi/(Xi+Xa)
1.0
0.8
0.6
0.4
0.2
0.0
Primed
Naive
EXMC
TSC
F
H9 T13 TSC
clone-1.3
clone-1.4
clone-1.5
clone-1.7
clone-1.8
Bright Field
DAPI/
XIST/
POLA1
G
POLA1 FISH quantification
Fraction of POLA1+ nuclei
100%
90%
80%
70%
60%
50%
40%
30%
20%
10%
0%
Bulk
1.3
1.4
1.5
1.7
1.8
NC
77
126
101
84
96
216
Bi-allelic
Mono-allelic

Figure S2: XIST skewing is uncoupled from repressive mark enrichment, related to Figure 2.

A. Representative IF-FISH of *XIST* (Green) and H2AK119ub (Magenta) images showing co-localization of the repressive mark with *XIST* in H9 naive, EXMCs and TSCs. Scale bar, 5 μm.

B. Representative IF-FISH of *XIST* (Green) and H3K27me3 (Magenta) images showing co-localization of the repressive mark with *XIST* in H9 naive, EXMCs and TSCs. Scale bar, 5 μm.

C. Heatmap showing Spearman correlation coefficients of the CUT&RUN signals on the X chromosome between all samples in H9 naive hESCs, EXMCs and TSCs.

D. H3K27me3 CUT&RUN tracks (log2 enrichment over IgG) around NANOG, TFCP2L1, CDX2, HAND1, GATA4, and HOXD10 in naive hESCs, EXMCs and TSCs.

E. Box plot showing the allelic ratio of CUT&RUN reads from Xi/Xtotal for IgG in Naive, EXMCs, and TSCs.

F. Representative brightfield images of various H9 TSC clonal lines (passage 13), each derived from a single cell (top panel). Scale bar: 1000 μm. Corresponding FISH images of the same clonal lines showing *XIST* (green) and *POLA1* (red) expression (middle panel). Scale bar: 50 μm. Cropped images of individual nuclei from the top panel, highlighting nuclear morphology (bottom panel). Scale bar: 5 μm.

G. Bar plots showing the quantification of the proportion of cells with monoallelic (Red) and biallelic (Gray) POLA1 expression in the different clonal H9 TSCs of the previous panel. The number of nuclei counted is indicated in supplemental table S1. Source data are provided as a Source Data file.

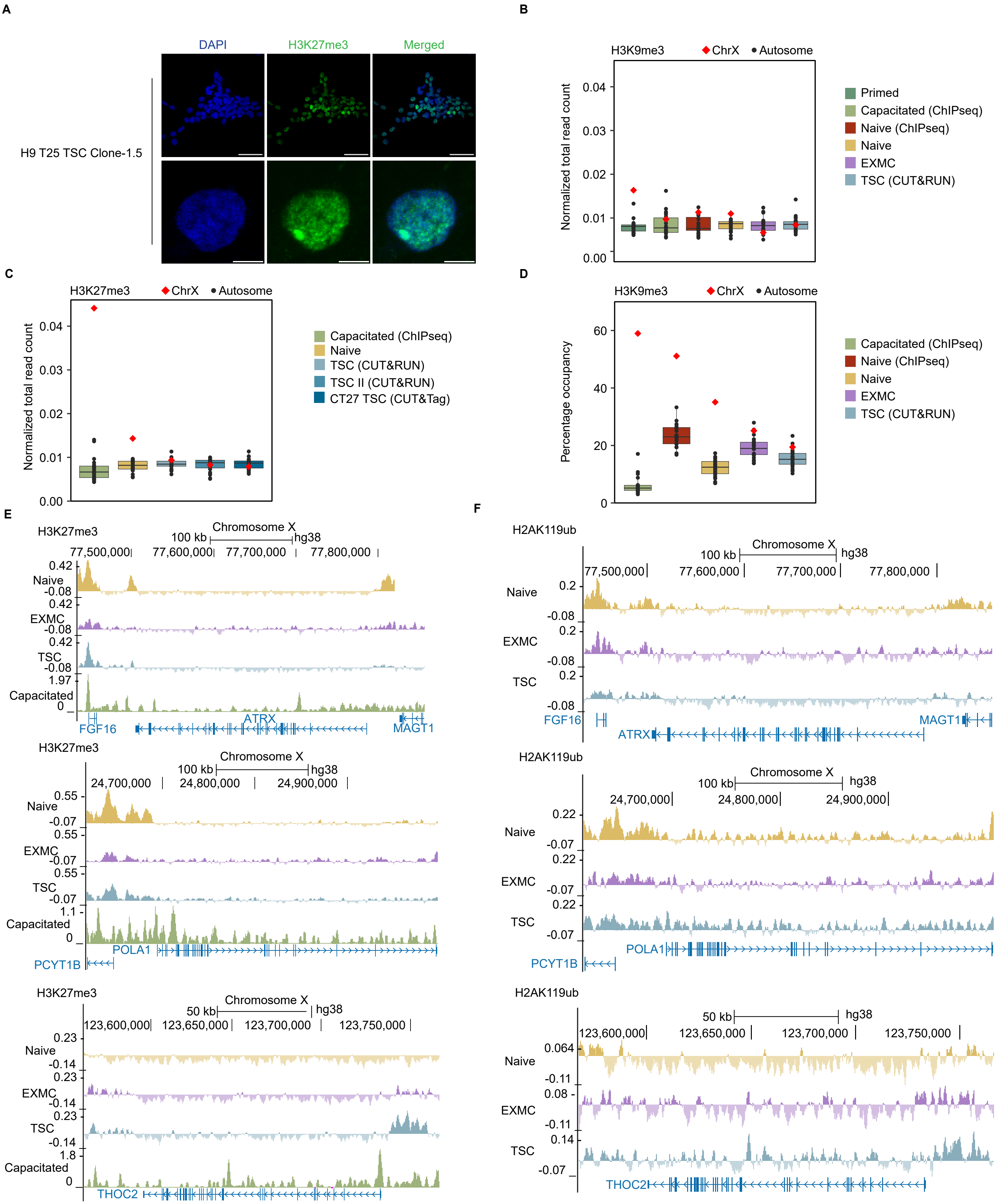

A
DAPI
H3K27me3
Merged
H9 T25 TSC Clone-1.5
B
H3K9me3
ChrX
Autosome
Normalized total read count
Primed
Capacitated (ChIPseq)
Naive (ChIPseq)
Naive
EXMC
TSC (CUT&RUN)
C
H3K27me3
ChrX
Autosome
Normalized total read count
Capacitated (ChIPseq)
Naive
TSC (CUT&RUN)
TSC II (CUT&RUN)
CT27 TSC (CUT&Tag)
D
H3K9me3
ChrX
Autosome
Percentage occupancy
Capacitated (ChIPseq)
Naive (ChIPseq)
Naive
EXMC
TSC (CUT&RUN)
E
H3K27me3
Chromosome X
100 kb
hg38
Naive
EXMC
TSC
Capacitated
FGF16
ATRX
MAGT1
POLA1
PCYT1B
50 kb
THOC2
F
H2AK119ub

Figure S3: Allelic analysis shows repressive marks not enriched on Xi, related to Figure 2.

A. Representative IF images of H3K27me3 (green) and DAPI (blue) in H9 TSC clone-1.5 (passage 25) (top panel). Scale bar, 50 μm. Cropped images of individual nuclei from the top panel, highlighting nuclear morphology (bottom panel). Scale bar: 5 μm.

B. Total number of CUT&RUN H3K9me3 reads from each chromosome, normalized by the length of each chromosome in primed hESCs, capacitated hESCs (ChIPseq data), naive (ChIPseq data), naive hESCs, EXMCs and TSCs.

C. Total number of H3K27me3 reads from each chromosome, normalized by the length of each chromosome in Capacitated cells hESCs (ChIPseq), naive hESCs, TSCs, H9 TSCs II (CUT&RUN) and CT27 TSCs (CUT&Tag).

D. Percentage of H3K9me3 peaks occupancy per chromosome in capacitated cells hESCs (ChIPseq data), naive hESCs (ChIPseq data), H9 naive hESCs, EXMCs and TSCs.

E. H3K27me3 CUT&RUN tracks (log2 enrichment over IgG) around the *ATRX*, *POLA1* and *THOC2* genes in naive hESCs, EXMCs and TSCs.

F. H2AK119ub CUT&RUN tracks (log2 enrichment over IgG) around the *ATRX*, *POLA1* and *THOC2* genes in naive hESCs, EXMCs and TSCs.

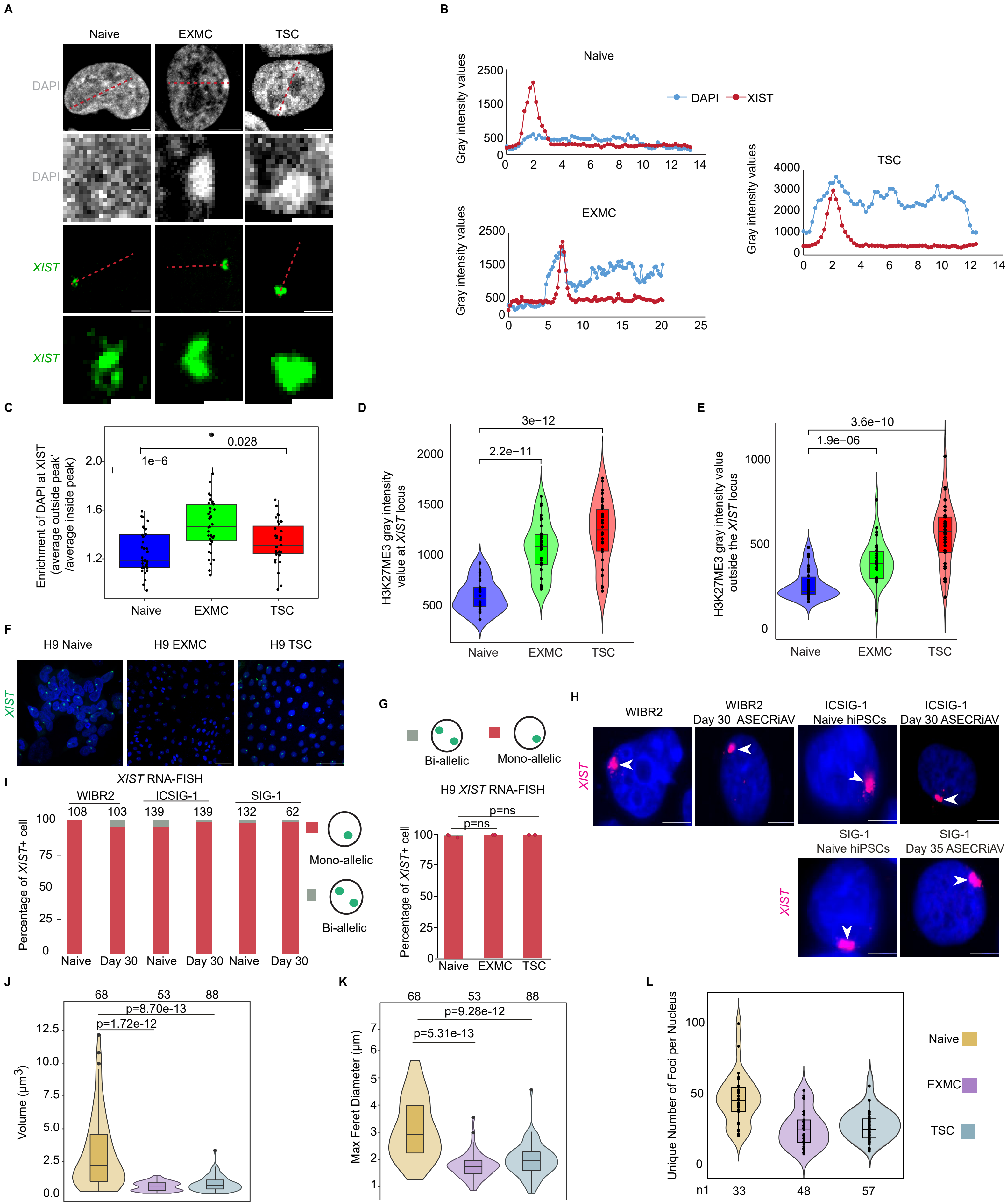

A
Naive
EXMC
TSC
DAPI
DAPI
XIST
XIST
B
Naive
EXMC
TSC
DAPI
XIST
Gray intensity values
C
Enrichment of DAPI at XIST (average outside peak' /average inside peak)
1e-6
0.028
Naive
EXMC
TSC
D
H3K27ME3 gray intensity value at XIST locus
3e-12
2.2e-11
Naive
EXMC
TSC
E
H3K27ME3 gray intensity value outside the XIST locus
3.6e-10
1.9e-06
Naive
EXMC
TSC
F
H9 Naive
H9 EXMC
H9 TSC
XIST
G
Bi-allelic
Mono-allelic
H9 XIST RNA-FISH
p=ns
p=ns
Percentage of XIST+ cell
Naive
EXMC
TSC
H
WIBR2
WIBR2 Day 30 ASECRiAV
ICSIG-1 Naive hiPSCs
ICSIG-1 Day 30 ASECRiAV
SIG-1 Naive hiPSCs
SIG-1 Day 35 ASECRiAV
XIST
XIST
I
XIST RNA-FISH
WIBR2
ICSIG-1
SIG-1
108
103
139
139
132
62
Percentage of XIST+ cell
Naive
Day 30
Naive
Day 30
Naive
Day 30
Mono-allelic
Bi-allelic
J
68
53
88
p=8.70e-13
p=1.72e-12
Volume (μm3)
K
68
53
88
p=9.28e-12
p=5.31e-13
Max Feret Diameter (μm)
L
Unique Number of Foci per Nucleus
n1
33
48
57
Naive
EXMC
TSC

Figure S4: Inactive X compaction underlies repressive mark enrichment, related to Figure 2.

A. Representative FISH of DAPI (blue) and *XIST* (green) in H9 naive, EXMC and TSC. Scale bar, 5 µm. Zoomed-in views of the XIST region shown below. Scale bar: 2 µm.

B. Representative line plots of DAPI (blue) and *XIST* (red) gray intensity across a line through the *XIST* cloud, for one nucleus each in naive, EXMC, and TSC.

C. Quantification of the average DAPI gray intensity at *XIST* foci versus outside in naive, EXMC, and TSC from A; at least 30 nuclei per cell type; two-sided t-test.

D. Quantification of H3K27me3 gray intensity value at the *XIST* locus. At least 35 nuclei per cell type; Wilcoxon test, naive vs EXMC and naive vs TSC.

E. As in D, for H3K27me3 gray intensity outside the XIST locus.

F. Representative RNA FISH of *XIST* (green) in H9 naive hESCs, EXMCs and TSCs. Scale bar, 50 µm.

G. Quantification of the cells with biallelic versus monoallelic *XIST* clouds from F. Nuclei countS below each bar. Mean from 3 experiments (n = 3), in triplicate. Logistic regression test. P-values are not significant.

H. Representative RNA FISH of *XIST* (magenta) in three different genetic backgrounds of naive hPSCs and day 30 trophoblast medium cells. DAPI in blue.

I. Quantification of biallelic versus monoallelic *XIST* clouds in three genetic backgrounds of naive and trophoblast cells. Nuclei counts above each bar.

J. Maximum *XIST* clouds volume (in μm^3) in H9 naive, EXMCs and TSCs (colored as in L). Nuclei counts above each condition. Wilcoxon-rank-sum test.

K. Maximum *XIST* cloud feret diameter (in µm) in H9 naive, EXMC and TSCs (as colored in L). Nuclei counts above each condition. Wilcoxon-rank-sum test.

L. Number of *XIST* foci per cloud per nucleus in H9 naive (tan), EXMCs (purple) and TSCs (blue-gray), from F. Nuclei counts below each plot.

Unless noted, box and violin plots show median (center line), 25th/75th percentiles (box bounds), and whiskers to 1.5x interquartile range, with points beyond as outliers; applies to (C, D, E, J, K, L). All tests are two-sided.

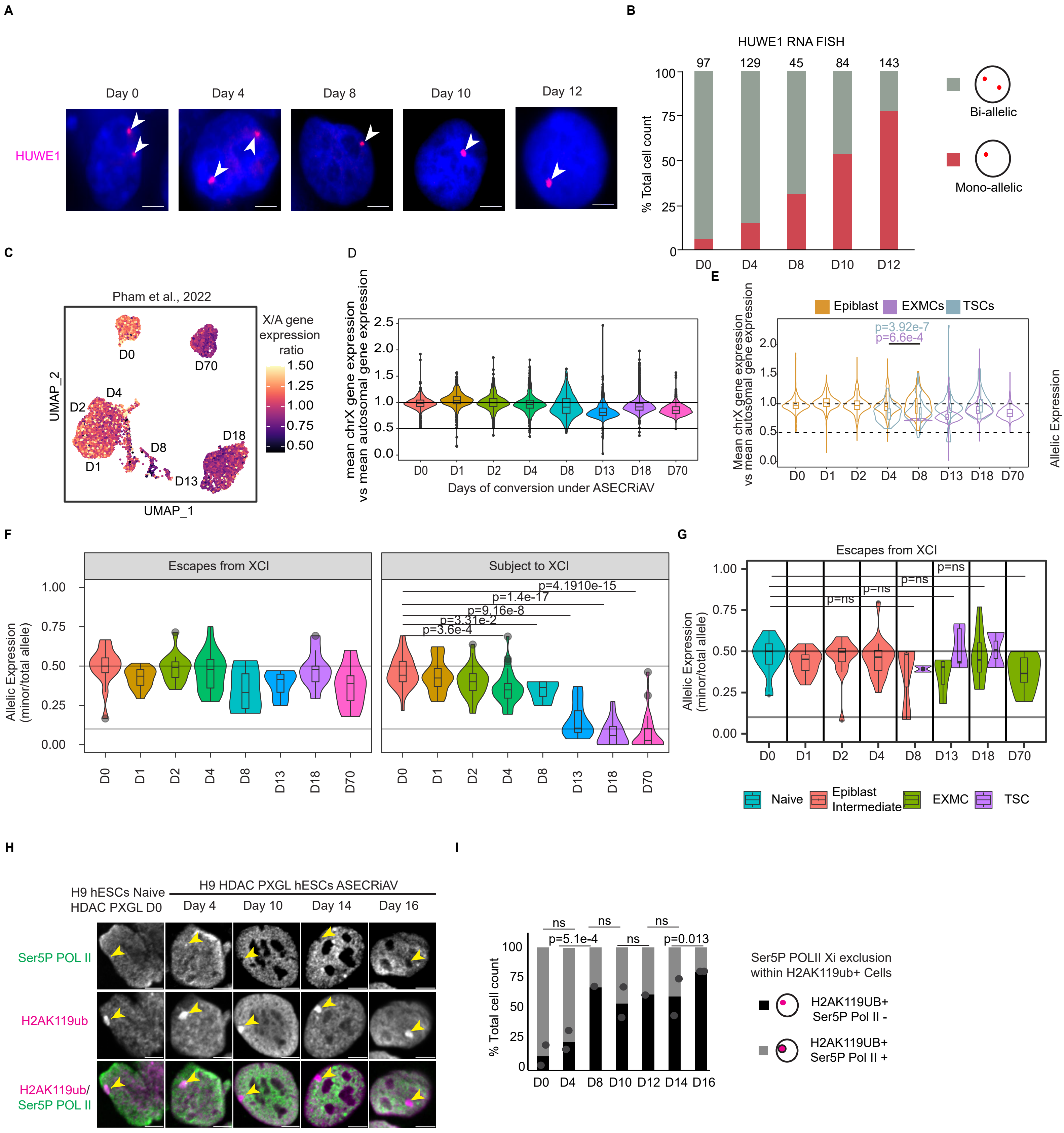
A
Day 0
Day 4
Day 8
Day 10
Day 12
HUWE1
B
HUWE1 RNA FISH
97
129
45
84
143
% Total cell count
D0
D4
D8
D10
D12
Bi-allelic
Mono-allelic
C
Pham et al., 2022
X/A gene expression ratio
UMAP_2
UMAP_1
D
mean chrX gene expression vs mean autosomal gene expression
Days of conversion under ASECRiAV
E
Epiblast
EXMCs
TSCs
p=3.92e-7
p=6.6e-4
Mean chrX gene expression vs mean autosomal gene expression
Allelic Expression
F
Escapes from XCI
Subject to XCI
Allelic Expression (minor/total allele)
p=4.1910e-15
p=1.4e-17
p=9.16e-8
p=3.31e-2
p=3.6e-4
G
Escapes from XCI
p=ns
Allelic Expression (minor/total allele)
Naive
Epiblast Intermediate
EXMC
TSC
H
H9 hESCs Naive HDAC PXGL D0
H9 HDAC PXGL hESCs ASECRiAV
Day 4
Day 10
Day 14
Day 16
Ser5P POL II
H2AK119ub
H2AK119ub/ Ser5P POL II
I
ns
p=5.1e-4
p=0.013
% Total cell count
D0
D4
D8
D10
D12
D14
D16
Ser5P POLII Xi exclusion within H2AK119ub+ Cells
H2AK119UB+ Ser5P Pol II -
H2AK119UB+ Ser5P Pol II +

Figure S5: XCI is temporally linked with extraembryonic identity, related to Figure 3.

A. RNA FISH of *HUWE1* expression dynamics during H9 5iLA-PXGL naive to EXMCs and TSCs differentiation. Samples were collected at five different time points as indicated.

B. Quantification of *HUWE1* puncta of panel A. Counted nuclei indicated above each bar.

C. Projection of the X to autosome gene expression ratio into the UMAP of time course scRNA-seq reanalysis of[2].

D. X:A gene expression ratio of time course scRNA-seq reanalysis of[2]. The data is shown with all cell types combined.

E. X:A gene expression ratio of time course scRNA-seq reanalysis of[2] with epiblast, TSCs, and EXMCs split apart. Two-sided t-test p-values are shown.

F. Allelic expression of time-course scRNA-seq reanalysis of [2], calculated using the scLinaX pipeline[3]. Data is presented with all cell types combined. Two-sided t-test p-values are shown.

G. Allelic expression of time-course scRNA-seq reanalysis of dataset[2], calculated using the scLinaX pipeline[3]. Data is presented with data split by cell types. Only genes expected to be inactivated are shown. Two-sided t-test p-values are shown.

H. Immunofluorescence of Ser5P POL II (green) and H2AK119ub (magenta) images showing dynamics during naive to TSCs-EXMCs conversion. Samples were collected at five different time points. Scale bar, 5 µm.

I. Quantification of Ser5P POL II enriched nuclei during the differentiation of naive to TSC and EXMCs in the previous panel.

Source data are provided as a Source Data file.

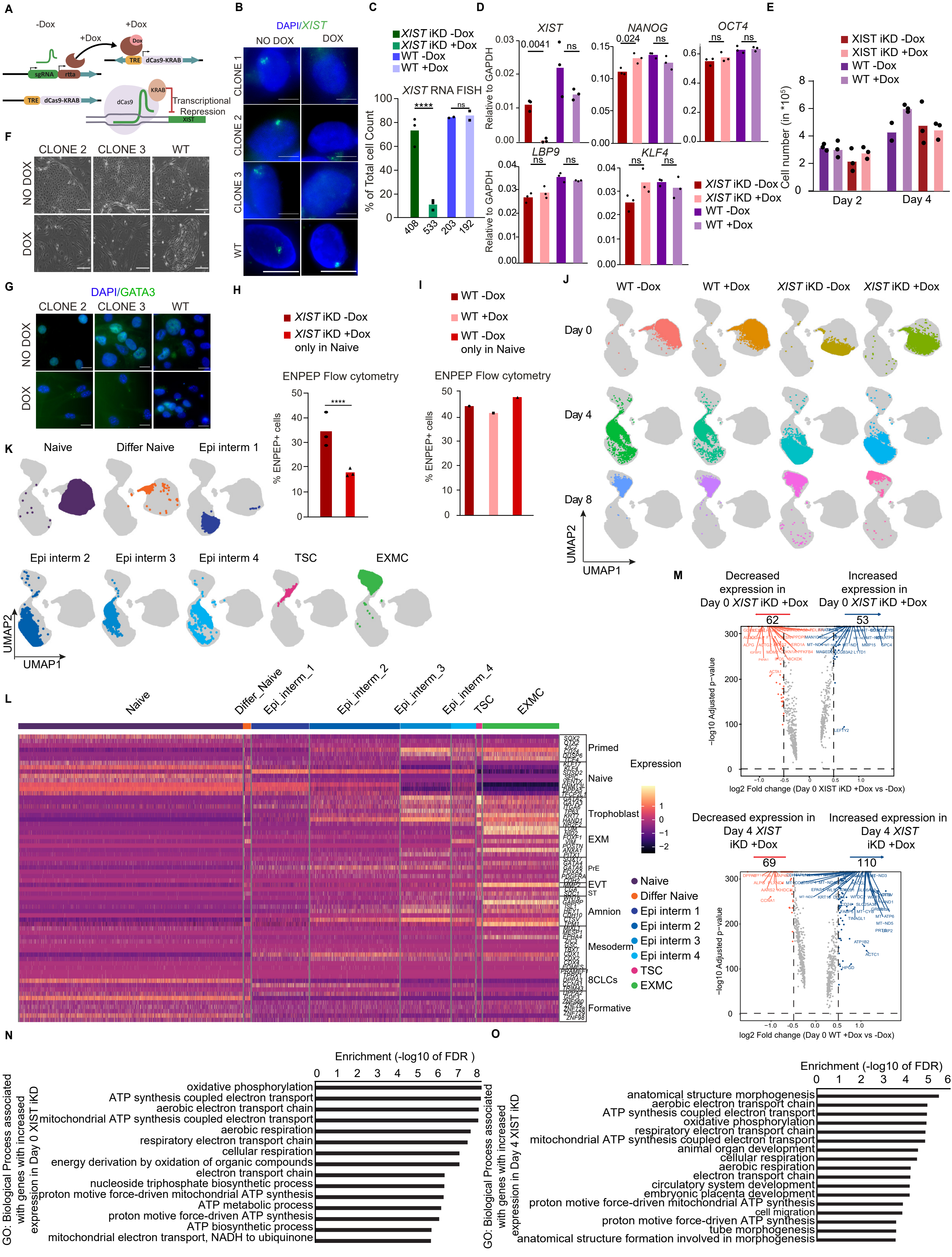
A
-Dox
+Dox
+Dox
sgRNA
rtta
TRE
dCas9-KRAB
KRAB
dCas9
Transcriptional Repression
XIST
B
DAPI/XIST
NO DOX
DOX
CLONE 1
CLONE 2
CLONE 3
WT
C
XIST iKD -Dox
XIST iKD +Dox
WT -Dox
WT +Dox
XIST RNA FISH
% of Total cell Count
408
533
203
192
D
XIST
NANOG
OCT4
LBP9
KLF4
Relative to GAPDH
0.0041
0.024
ns
E
Cell number (in *10^5)
Day 2
Day 4
F
CLONE 2
CLONE 3
WT
NO DOX
DOX
G
DAPI/GATA3
H
XIST iKD -Dox
XIST iKD +Dox only in Naive
ENPEP Flow cytometry
% ENPEP+ cells
I
WT -Dox
WT +Dox
WT -Dox only in Naive
J
WT -Dox
WT +Dox
XIST iKD -Dox
XIST iKD +Dox
Day 0
Day 4
Day 8
UMAP2
UMAP1
K
Naive
Differ Naive
Epi interm 1
Epi interm 2
Epi interm 3
Epi interm 4
TSC
EXMC
L
Naive
Differ_Naive
Epi_interm_1
Epi_interm_2
Epi_interm_3
Epi_interm_4
TSC
EXMC
Primed
Naive
Trophoblast
EXM
PrE
EVT
ST
Amnion
Mesoderm
8CLCs
Formative
Expression
M
Decreased expression in Day 0 XIST iKD +Dox
Increased expression in Day 0 XIST iKD +Dox
62
53
−log10 Adjusted p−value
log2 Fold change (Day 0 XIST iKD +Dox vs -Dox)
Decreased expression in Day 4 XIST iKD +Dox
Increased expression in Day 4 XIST iKD +Dox
69
110
log2 Fold change (Day 0 WT +Dox vs -Dox)
N
Enrichment (-log10 of FDR)
GO: Biological Process associated with genes with increased expression in Day 0 XIST iKD
oxidative phosphorylation
ATP synthesis coupled electron transport
aerobic electron transport chain
mitochondrial ATP synthesis coupled electron transport
aerobic respiration
respiratory electron transport chain
cellular respiration
energy derivation by oxidation of organic compounds
electron transport chain
nucleoside triphosphate biosynthetic process
proton motive force-driven mitochondrial ATP synthesis
ATP metabolic process
proton motive force-driven ATP synthesis
ATP biosynthetic process
mitochondrial electron transport, NADH to ubiquinone
O
Enrichment (-log10 of FDR)
GO: Biological Process associated with genes with increased expression in Day 4 XIST iKD
anatomical structure morphogenesis
aerobic electron transport chain
ATP synthesis coupled electron transport
oxidative phosphorylation
respiratory electron transport chain
mitochondrial ATP synthesis coupled electron transport
animal organ development
cellular respiration
aerobic respiration
electron transport chain
circulatory system development
embryonic placenta development
proton motive force-driven mitochondrial ATP synthesis
cell migration
proton motive force-driven ATP synthesis
tube morphogenesis
anatomical structure formation involved in morphogenesis

Figure S6: Loss of *XIST* impacts human extraembryonic cell survival, related to Figure 4.

A. Schematic of the inducible CRISPR-Cas9 iKD system used to repress *XIST* in female H9 hESCs.

B. Representative RNA FISH of *XIST* (green) in WT and *XIST* iKD clone-1, 2, 3 naive hESCs in PXGL medium with or without Dox. Scale bar, 10 μm. (n=3 iKD clones, n=2 WT clones)

C. Quantification of cells with *XIST* clouds from B, each dot= one clone. Chi² test (-Dox vs +Dox): P<0.0001 (iKD), P=0,1865 (WT); some comparisions exceeded test resolution (p<1e-16)

D. ET-qPCR of *XIST*, *NANOG, OCT4, KLF4,* and *LBP9*; each dot= one clone (n= 3 iKD, n=3 WT), unpaired t-test, p-values shown.

E. Cell proliferation of WT and *XIST* iKD naïve cells, untreated versus 10 days Dox, counted at 2 or 4 days after passage. Each dot = mean of 2 measure per clone. (n = 2-3 clones).

F. Bright-field images of H9 *XIST* iKD clone-2, 3, and WT hESCs morphology at Day 15 of trophoblast differentiation, with or without Dox. Scale bar, 200 μm. (n= 3 iKD and n=2 WT clones).

G. Immunofluorescence of GATA3 (green) in cells from F. DAPI in blue. Scale bar, 20 μm. (n=3 clones, 2 shown, one in Fig. 4C).

H, I. Quantification of ENPEP positive cells (flow cytometry) at Day 15: *XIST-depleted* versus control cells (H). Each dot= one clone (n=3), Chi² test, p<0,0001; and WT cells with Dox, without Dox, or Dox treated onely in naive state (I), each dot= one clone (n=1).

J. UMAP from 4F, colored by condition.

K. UMAP 4G, colored by cell type.

L. Heatmap of marker genes for primed pluripotency, core pluripotency, naive pluripotency, trophoblast and extraembryonic mesoderm (EXM), primitive endoderm (PrE), extravillous trophoblast (EVT), syncytiotrophoblast (ST), amnion, mesoderm, 8-cell-like-cells (8CLCs) and formative pluripotency.

M. Differential gene expression (scRNA-seq) between *XIST* iKD cells with Dox versus without Dox, Day 0 (top) and Day 4 (bottom). Dashed lines: -log10 adjusted p-Value <0.05 and log2 fold change < -0.5 or > 0.5.

N, O. GO enrichment of DEGs, Day 0 (N) and Day 4 (O) *XIST* iKD cells, with versus without Dox.

Source data are provided as a Source Data file.

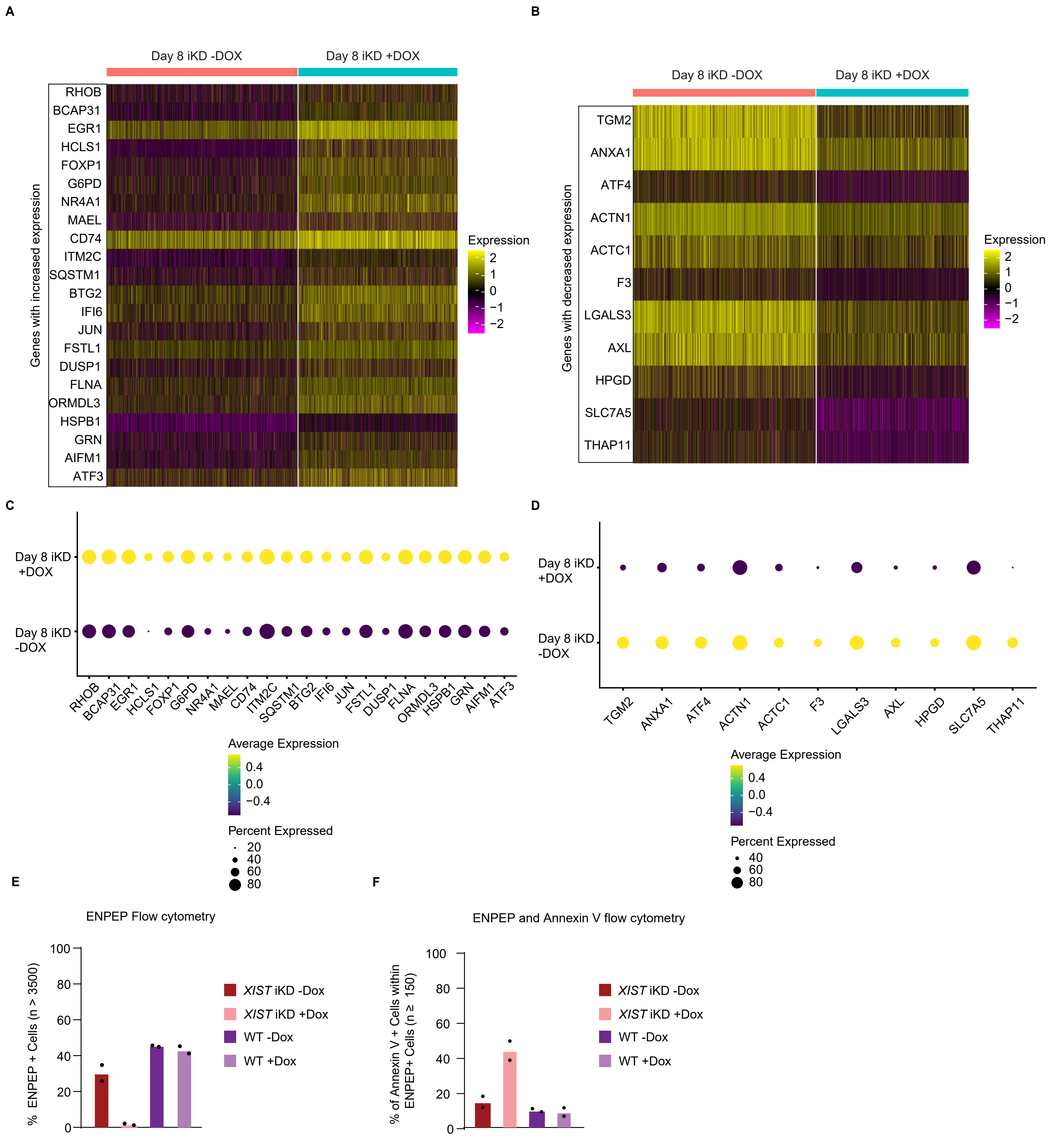

A
Day 8 iKD -DOX
Day 8 iKD +DOX
Genes with increased expression
RHOB
BCAP31
EGR1
HCLS1
FOXP1
G6PD
NR4A1
MAEL
CD74
ITM2C
SQSTM1
BTG2
IFI6
JUN
FSTL1
DUSP1
FLNA
ORMDL3
HSPB1
GRN
AIFM1
ATF3
Expression
2
1
0
−1
−2
B
Day 8 iKD -DOX
Day 8 iKD +DOX
Genes with decreased expression
TGM2
ANXA1
ATF4
ACTN1
ACTC1
F3
LGALS3
AXL
HPGD
SLC7A5
THAP11
Expression
2
1
0
−1
−2
C
Day 8 iKD +DOX
Day 8 iKD -DOX
RHOB
BCAP31
EGR1
HCLS1
FOXP1
G6PD
NR4A1
MAEL
CD74
ITM2C
SQSTM1
BTG2
IFI6
JUN
FSTL1
DUSP1
FLNA
ORMDL3
HSPB1
GRN
AIFM1
ATF3
Average Expression
0.4
0.0
−0.4
Percent Expressed
20
40
60
80
D
Day 8 iKD +DOX
Day 8 iKD -DOX
TGM2
ANXA1
ATF4
ACTN1
ACTC1
F3
LGALS3
AXL
HPGD
SLC7A5
THAP11
Average Expression
0.4
0.0
−0.4
Percent Expressed
40
60
80
E
ENPEP Flow cytometry
% ENPEP + Cells (n > 3500)
100
80
60
40
20
0
XIST iKD -Dox
XIST iKD +Dox
WT -Dox
WT +Dox
F
ENPEP and Annexin V flow cytometry
% of Annexin V + Cells within ENPEP+ Cells (n ≥ 150)
100
80
60
40
20
0
XIST iKD -Dox
XIST iKD +Dox
WT -Dox
WT +Dox

Figure S7: Loss of *XIST* induces apoptosis in trophoblast cells, related to Figure 4.

A. Heatmap of apoptotic DEGs associated with GO terms #1465, #1513, and #542 in day 8 *XIST* iKD cells, with or without *XIST*.

B. Heatmap showing decreased expression of apoptotic DEGs with the same GO terms.

C. Dot plot representation of all DEGs shown in A.

D. Dot plot representation of DEGs from B.

E. Relative proportion of ENPEP+ cells detected by FACS in WT and *XIST* iKD clones (+Dox, +Dox) at day 15 of ASECRiAV differentiation. 2 replicate clones were used per condition tested, collecting both attached and supernatant cells on the day of the experiment.

F. Percentage of Annexin V+ cells within the gated ENPEP+ cell population. 2 replicate clones were used per condition tested, collecting both attached and supernatant cells on the day of the experiment.

A

B

C

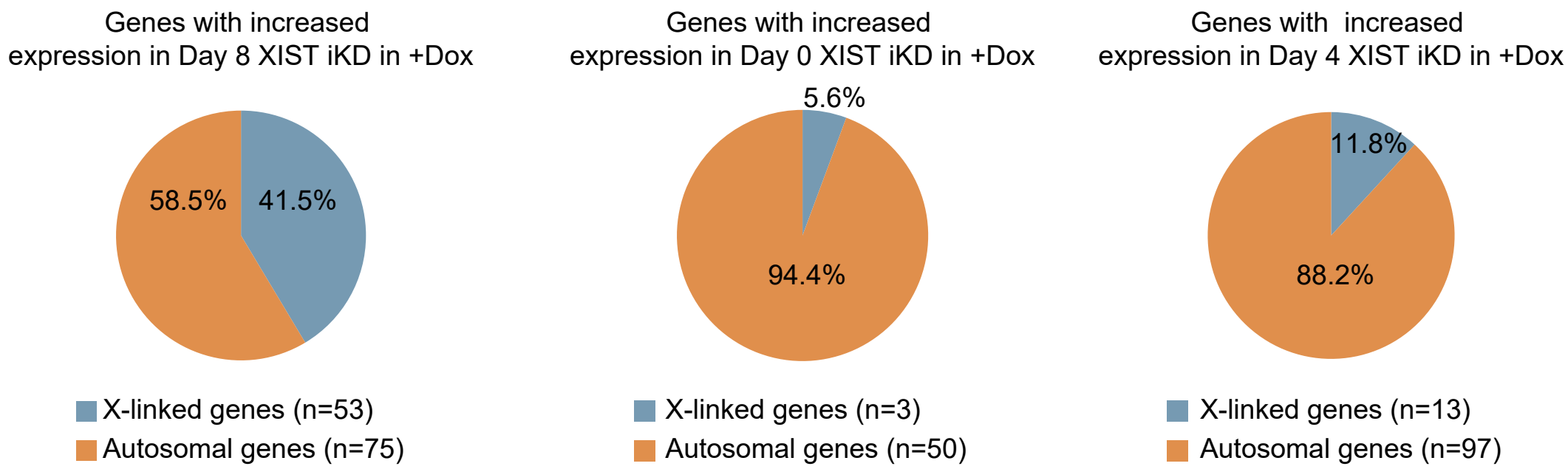

Genes with increased expression in Day 8 XIST iKD in +Dox
58.5%
41.5%
X-linked genes (n=53)
Autosomal genes (n=75)
Genes with increased expression in Day 0 XIST iKD in +Dox
5.6%
94.4%
X-linked genes (n=3)
Autosomal genes (n=50)
Genes with increased expression in Day 4 XIST iKD in +Dox
11.8%
88.2%
X-linked genes (n=13)
Autosomal genes (n=97)

D

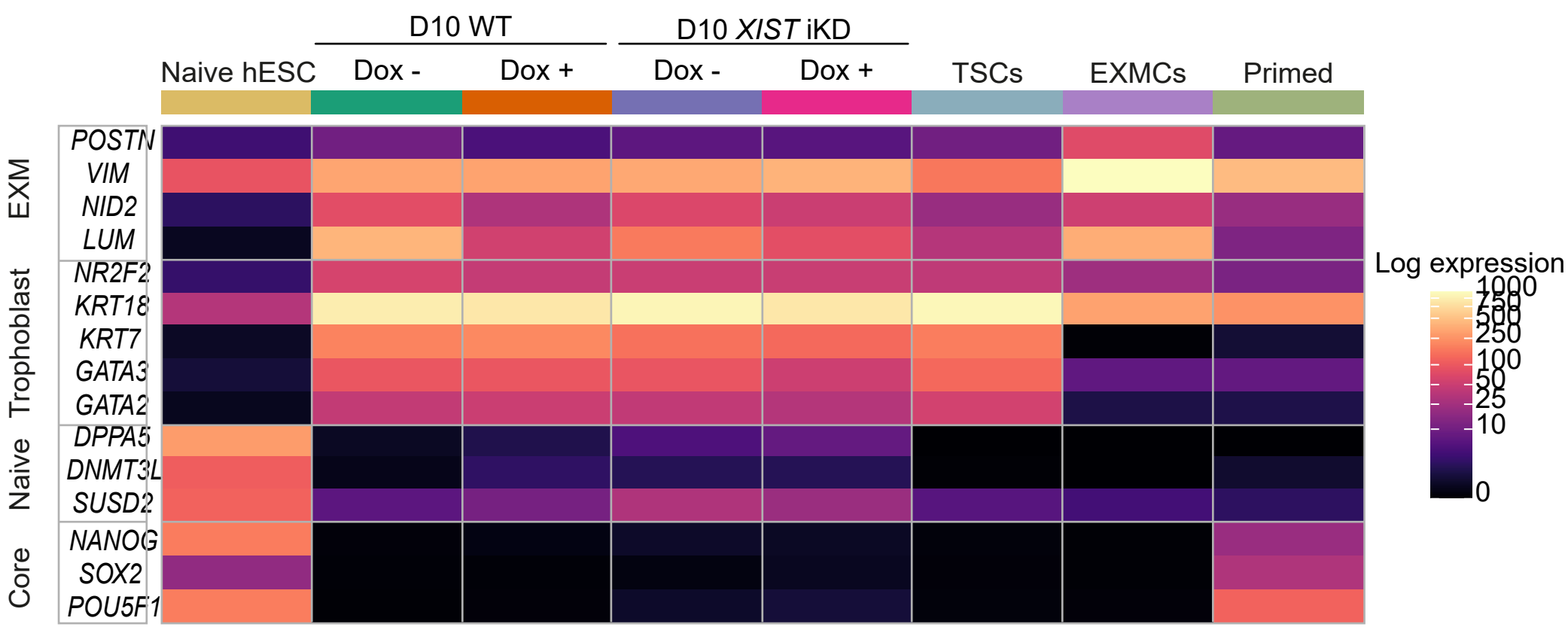

D10 WT
D10 XIST iKD
Naive hESC
Dox -
Dox +
Dox -
Dox +
TSCs
EXMCs
Primed
EXM
Trophoblast
Naive
Core
POSTN
VIM
NID2
LUM
NR2F2
KRT18
KRT7
GATA3
GATA2
DPPA5
DNMT3L
SUSD2
NANOG
SOX2
POU5F1
Log expression
1000
750
500
250
100
50
25
10
0

Figure S8: *XIST* loss prevents cells from undergoing XCI, related to Figure 5.

A. Proportion of DEGs in Day 8 *XIST* depleted cells compared to control (-Dox) cells corresponding to autosomal and X-linked genes. n is the number of genes.

B. Proportion of DEGs in Day 0 *XIST* depleted cells compared to control (-Dox) cells corresponding to autosomal and X-linked genes. n is the number of genes.

C. Proportion of DEGs in Day 4 *XIST* depleted cells compared to control (-Dox) cells belonging to autosomal and X-linked genes. n is the number of genes.

D. Heatmap showing the log2 expression of key marker genes for EXM, trophoblast, naive, and core pluripotency in naive, day 10 WT -Dox, WT +Dox, *XIST* iKD -Dox and *XIST* iKD +Dox, TSCs, EXMCs and primed hESCs.

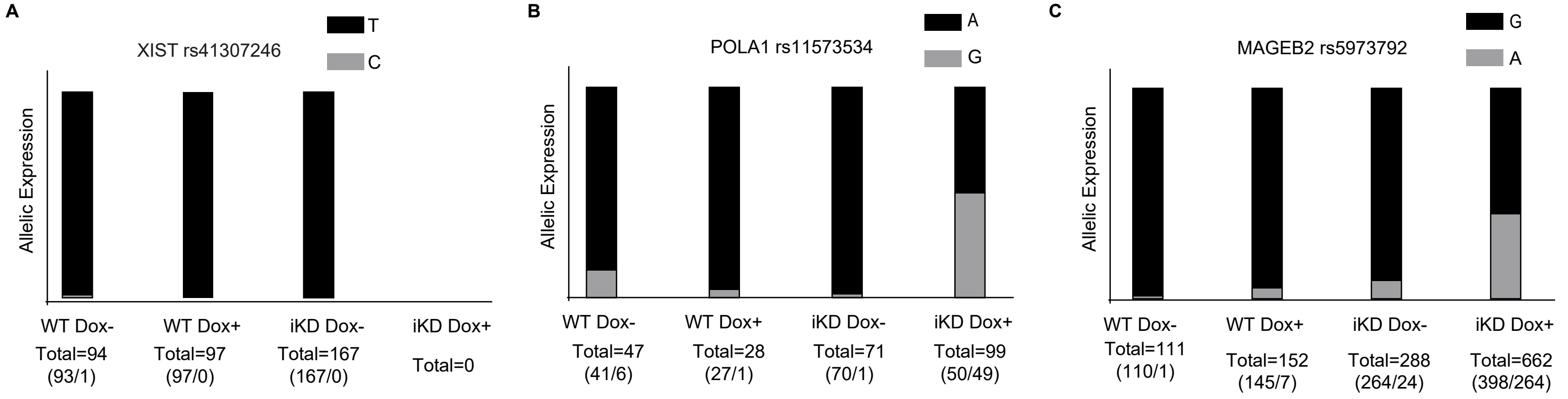
A
XIST rs41307246
T
C
Allelic Expression
WT Dox-
Total=94
(93/1)
WT Dox+
Total=97
(97/0)
iKD Dox-
Total=167
(167/0)
iKD Dox+
Total=0
B
POLA1 rs11573534
A
G
Allelic Expression
WT Dox-
Total=47
(41/6)
WT Dox+
Total=28
(27/1)
iKD Dox-
Total=71
(70/1)
iKD Dox+
Total=99
(50/49)
C
MAGEB2 rs5973792
G
A
Allelic Expression
WT Dox-
Total=111
(110/1)
WT Dox+
Total=152
(145/7)
iKD Dox-
Total=288
(264/24)
iKD Dox+
Total=662
(398/264)

Figure S9: Allelic analysis confirms impairment of XCI, related to Figure 5.
Allelic expression of *XIST* (rs41307246), *POLA1* (rs11573534) and *MAGEB2* (rs5973792) was measured in Day 10 WT and *XIST* iKD with or without Dox, on a *XIST* iKD or a WT clone (n=1). Total number of informative reads over the SNPs and proportion are indicated for each condition.

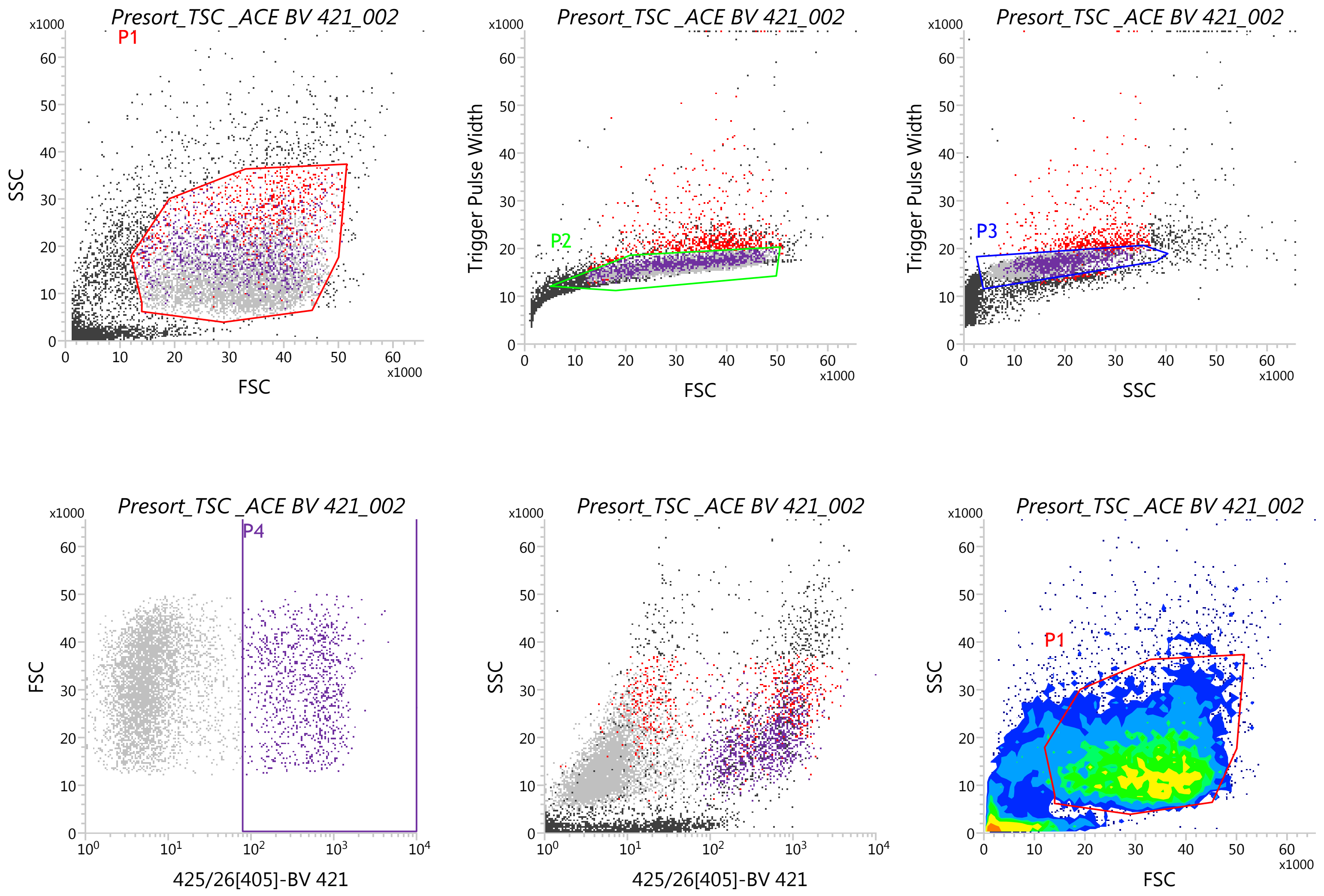

Populations: *Presort_TSC _ACE BV 421_002*

Populations	Even...	% Total	% Parent
All Events	10,000	100.00%	####
P1	6,462	64.62%	64.62%
P2	5,758	57.58%	89.11%
P3	5,817	58.17%	90.02%
P2 AND P3	5,694	56.94%	88.12%
P4	989	9.89%	17.37%

EXTENDED DATA FIGURES

Extended data figure 1: Flow cytometry gating strategy for TSC sorting, related to Figure S1A.

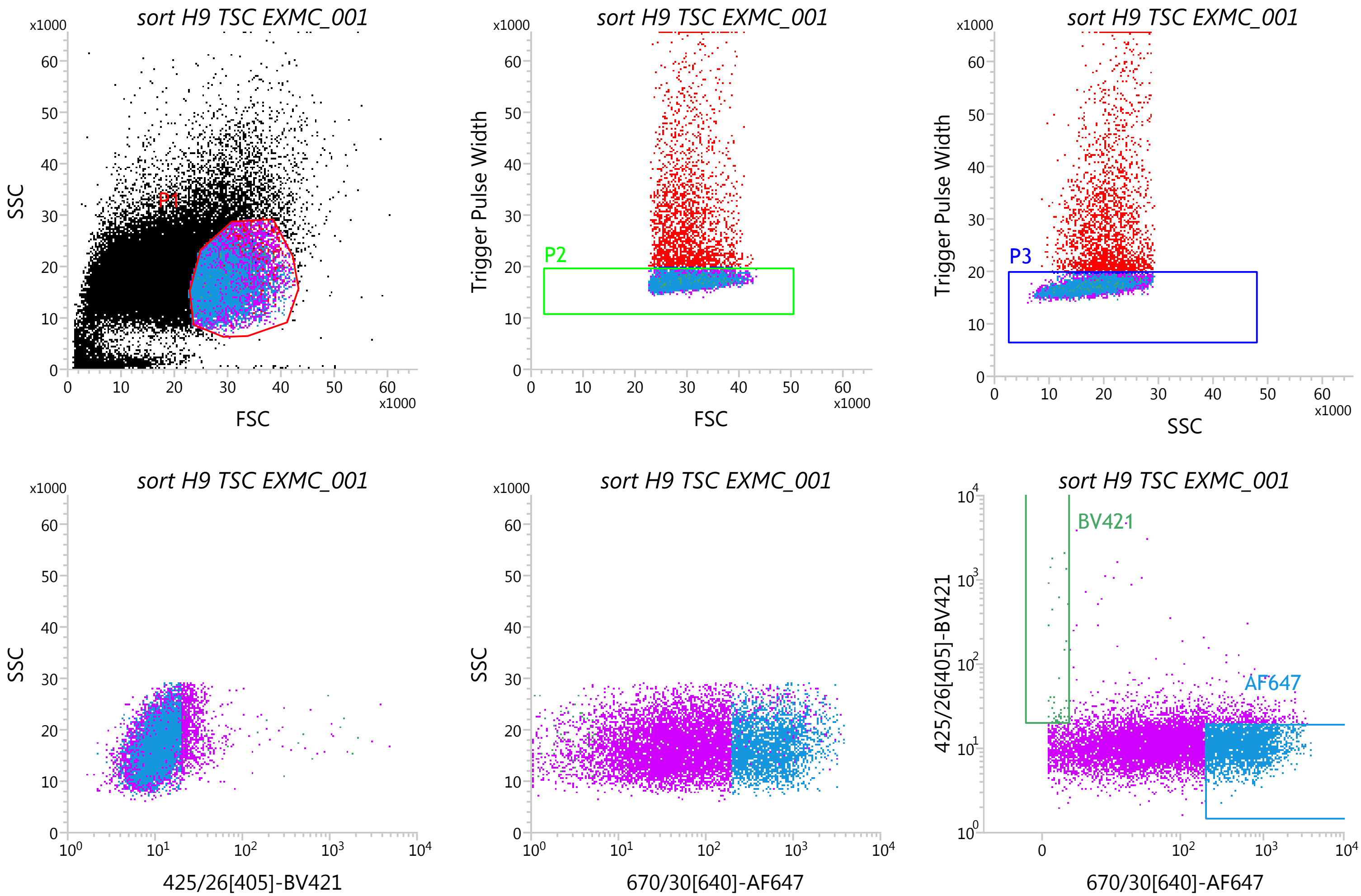

Populations: *sort H9 TSC EXMC_001*

Populations	Even...	% Total	% Parent
All Events	50,000	100.00%	####
P1	12,911	25.82%	25.82%
P2	10,504	21.01%	81.36%
P3	10,578	21.16%	81.93%
P2 AND P3	10,504	21.01%	81.36%
AF647	2,879	5.76%	27.41%
BV421	40	0.08%	0.38%

Extended data figure 2: Flow cytometry gating strategy for EXMC sorting, related to Figure S1A.

Related to Figure 4E and Supplemental Figure 6H, 6I
clone-1 = A11 condition dox treated

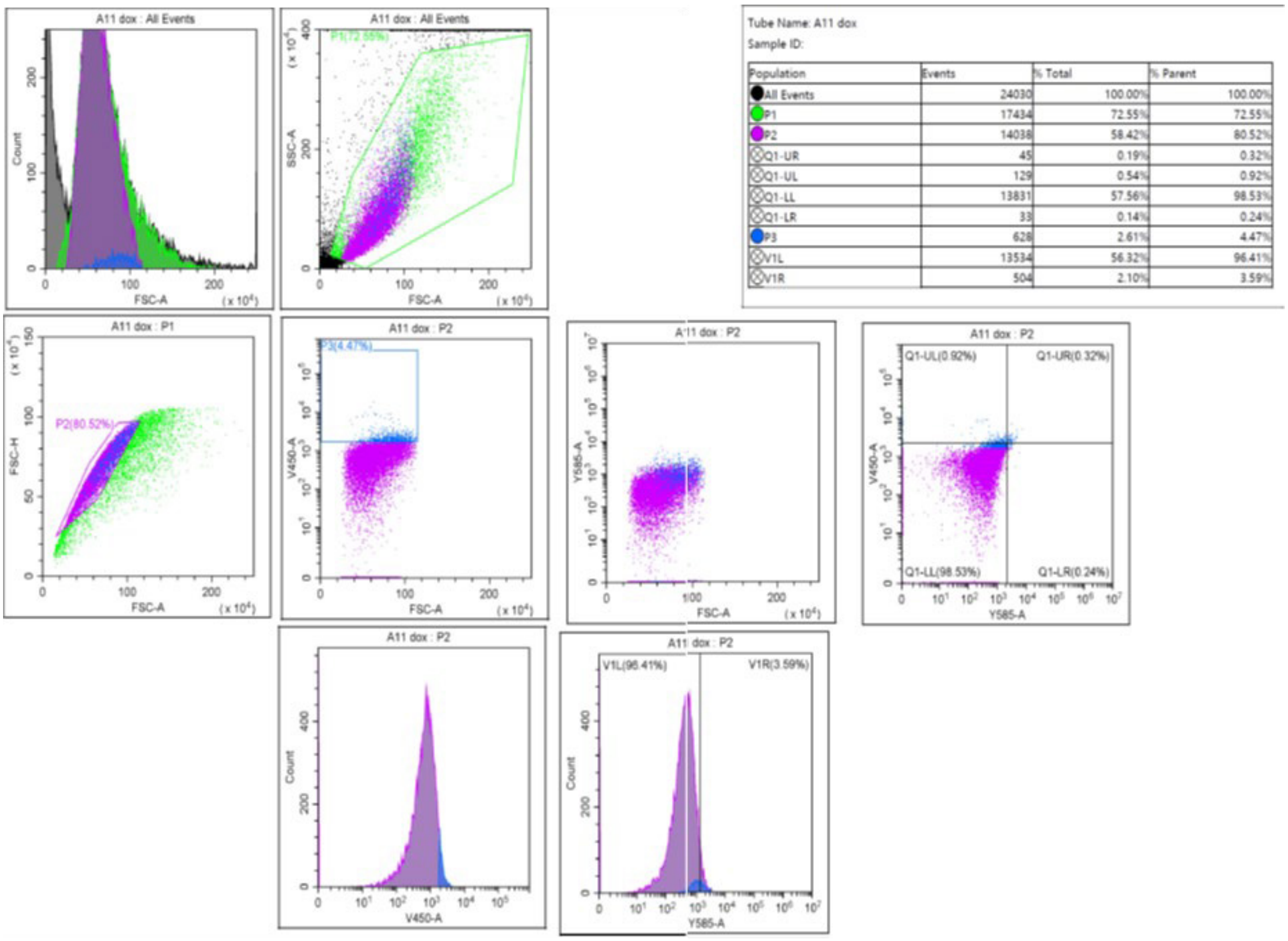

Tube Name: A11 dox
Sample ID:

Population	Events	% Total	% Parent
All Events	24030	100.00%	100.00%
P1	17434	72.55%	72.55%
P2	14038	58.42%	80.52%
Q1-UR	45	0.19%	0.32%
Q1-UL	129	0.54%	0.92%
Q1-LL	13831	57.56%	98.53%
Q1-LR	33	0.14%	0.24%
P3	628	2.61%	4.47%
V1L	13534	56.32%	96.41%
V1R	504	2.10%	3.59%

A11 = Clone-1 condition vehicle treated

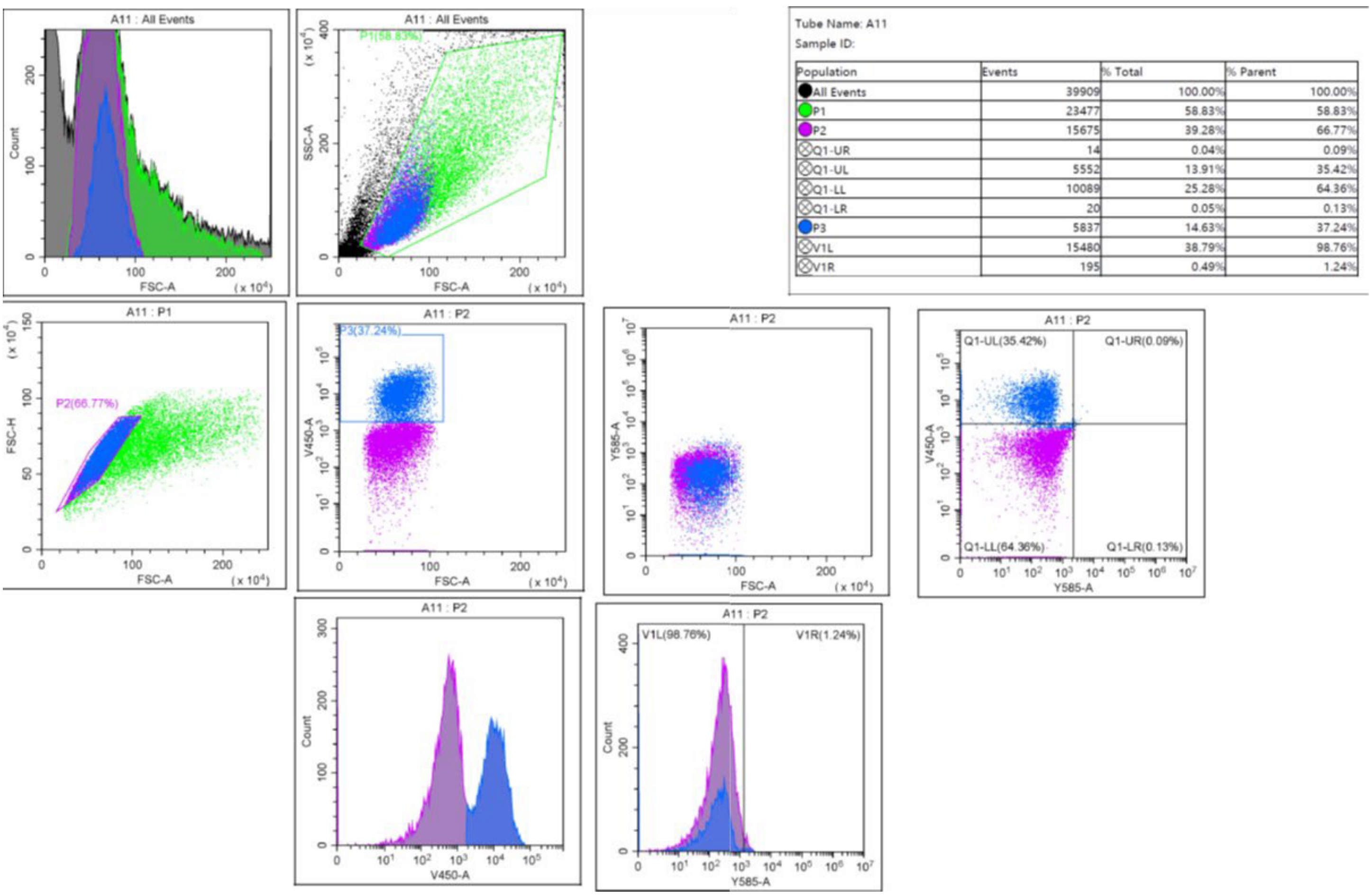

Tube Name: A11
Sample ID:

Population	Events	% Total	% Parent
All Events	39909	100.00%	100.00%
P1	23477	58.83%	58.83%
P2	15675	39.28%	66.77%
Q1-UR	14	0.04%	0.09%
Q1-UL	5552	13.91%	35.42%
Q1-LL	10089	25.28%	64.36%
Q1-LR	20	0.05%	0.13%
P3	5837	14.63%	37.24%
V1L	15480	38.79%	98.76%
V1R	195	0.49%	1.24%

Extended data figure 3: Flow cytometry gating strategy for quantification of ENPEP+ cells with or without DOX in *XIST* iKD cells at day 15 of differentiation, related to Figures 4E, S6H, and S6I.

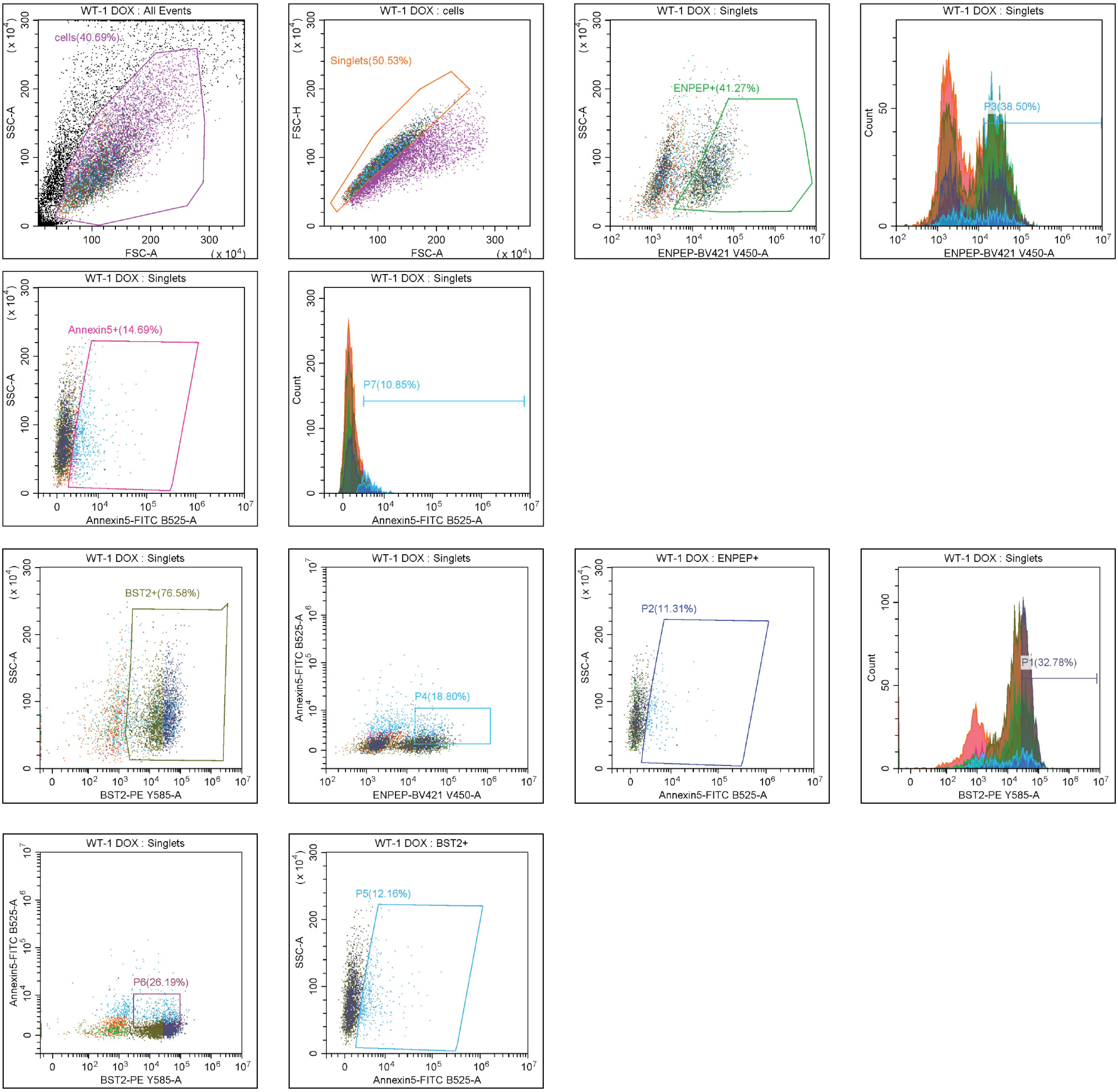

Tube Name: WT-1 DOX

Sample ID:

Population	Events	% Total	% Parent
All Events	20000	100.00%	100.00%
cells	8137	40.69%	40.69%
Singlets	4112	20.56%	50.53%
P3	1583	7.92%	38.50%
Annexin5+	604	3.02%	14.69%
P4	773	3.87%	18.80%
P6	1077	5.39%	26.19%
ENPEP+	1697	8.49%	41.27%
P7	446	2.23%	10.85%
BST2+	3149	15.75%	76.58%
P1	1348	6.74%	32.78%

Extended data figure 4: Flow cytometry gating strategy for quantification of ENPEP+ and Annexin V+ cells with or without DOX in *XIST* iKD cells at day 15 of differentiation, related to Figures S7E and S7F.

Key Resources Tables

qPCR primers list

Gene	Forward	Reverse
XIST	ACATGCCTGGCACTCTAGCA	AAACATGGAAATGGGTAAGACACA
NANOG	GCAATGGTGTGACGCAGAAG	GCATCCCTGGTGGTAGGAAGA
OCT4	CCCCTGGTGCCGTGAAG	CTCGAGTTCTTTCTGCAGAGCTT
KLF4	GGGAGAAGACACTGCGTCA	GGAAGCACTGGGGGAAGT
LBP9	GCTCTTCAACGCCATCAAA	CAGGGGCACTCGATTCTG
GATA3	GGGAGAGAGAGAGAGAAGAAGA	AACTGGGTACGGCAGAATAAA
GAPDH	CTTAGATTTGGTCGTATTGG	CTTCCCGTTCTCAGCCTTGACG
Sly_Xlr	GATGATTTGAGTGGAAATGTGAGGTA	CTTATGTTTATAGGCATGCACCATGTA

CRISPRi sgRNA

Gene	Forward	Reverse
XIST	CACCGATACGCCATAAAGGGTGTTG	AAACCAACACCCTTTATGGCGTATC

IF antibodies list

Reagent	Source	Identifier
Rat monoclonal anti-GATA3	Thermo Fisher	14-9966-82
Mouse monoclonal anti-Vimentin	Abcam	ab8978
Rabbit polyclonal anti-KLF17	Sigma-Aldrich	HPA024629
Rabbit monoclonal anti-H2AK119ub	Cell Signalling	8240s

Mouse monoclonal anti-H3K27me3	Active motif	61017
Ser5P Polymerase II	Abcam	ab5408
Annexin V	Invitrogen	11-800572
CD249 (ENPEP)	BD Biosciences	744872
Annexin V Apoptosis Detection Kit FITC	Invitrogen	88-8005-72

<u>CUT&RUN and CUT&TAG antibodies list</u>

Reagent	Source	Identifier
Rabbit polyclonal anti-H3K9me3	Active motif	Cat#39062
Rabbit polyclonal anti-H3K27me3	Active motif	Cat#39055
Rabbit monoclonal anti-H2AK119ub	Cell Signaling	Cat#8240S
Rabbit polyclonal anti-IgG	Diagenode	Cat#C15410206
Rabbit mono-clonal anti-H3K27me3	Cell Signaling	cat# 9733

References

1. Balaton, B. P., Cotton, A. M. & Brown, C. J. Derivation of consensus inactivation status for X-linked genes from genome-wide studies. *Biol. Sex Differ.* **6**, 35 (2015).
2. Pham, T. X. A. *et al.* Modeling human extraembryonic mesoderm cells using naive pluripotent stem cells. *Cell Stem Cell* **29**, 1346–1365.e10 (2022).

3. Tomofuji, Y. *et al.* Quantification of escape from X chromosome inactivation with single-cell omics data reveals heterogeneity across cell types and tissues. *Cell Genom* **4**, 100625 (2024).
