# Supplementary material for: XIST drives X-chromosome inactivation and safeguards female extraembryonic cells in humans
Source: Nat Commun. 2026 Oct 22;17:10738. doi: 10.1038/s41467-026-78394-z (PMC13691081; doi:10.1038/s41467-026-78394-z)
Supplement: Supplementary file 3 [file 41467_2026_78394_MOESM2_ESM.pdf]

nature portfolio

Corresponding author(s): Professor Dr. Jean-François Ouimette
Professor Dr. Claire Rougeulle
Professor Dr. Vincent Pasque

Last updated by author(s): 01/09/2026

# Reporting Summary

Nature Portfolio wishes to improve the reproducibility of the work that we publish. This form provides structure for consistency and transparency in reporting. For further information on Nature Portfolio policies, see our Editorial Policies and the Editorial Policy Checklist.

## Statistics

For all statistical analyses, confirm that the following items are present in the figure legend, table legend, main text, or Methods section.

| n/a | Confirmed | |
|---|---|---|
| ☐ | ☒ | The exact sample size (*n*) for each experimental group/condition, given as a discrete number and unit of measurement |
| ☐ | ☒ | A statement on whether measurements were taken from distinct samples or whether the same sample was measured repeatedly |
| ☐ | ☒ | The statistical test(s) used AND whether they are one- or two-sided<br>*Only common tests should be described solely by name; describe more complex techniques in the Methods section.* |
| ☒ | ☐ | A description of all covariates tested |
| ☐ | ☒ | A description of any assumptions or corrections, such as tests of normality and adjustment for multiple comparisons |
| ☐ | ☒ | A full description of the statistical parameters including central tendency (e.g. means) or other basic estimates (e.g. regression coefficient) AND variation (e.g. standard deviation) or associated estimates of uncertainty (e.g. confidence intervals) |
| ☐ | ☒ | For null hypothesis testing, the test statistic (e.g. *F*, *t*, *r*) with confidence intervals, effect sizes, degrees of freedom and *P* value noted<br>*Give P values as exact values whenever suitable.* |
| ☒ | ☐ | For Bayesian analysis, information on the choice of priors and Markov chain Monte Carlo settings |
| ☐ | ☒ | For hierarchical and complex designs, identification of the appropriate level for tests and full reporting of outcomes |
| ☐ | ☒ | Estimates of effect sizes (e.g. Cohen's *d*, Pearson's *r*), indicating how they were calculated |

*Our web collection on statistics for biologists contains articles on many of the points above.*

## Software and code

Policy information about availability of computer code

| | |
|---|---|
| Data collection | 10X scRNA-Seq, Bulk RNA-Seq, DNA-Med-Seq, CUT&RUN, CUT&TAG |
| Data analysis | FlowJo Vl0.l software<br>Nikon NIS-Elements AR with DeNoise Al and 3D Richardson-Lucy algorithm (vS.30.01)<br>Axio Vision software (v4.9.l.0)<br>ImageJ (vl.37a)<br>Microsoft Excel (Microsoft Office Professional Plus 2016) R (v4.0.3)<br>ggplot2 (v3.3.3)<br>FastQC<br>cutAdapt<br>bedtools (2.30.0)<br>bcftools mpileup<br>macs2 (2.2.7.1)<br>R (4.3.2)<br>R (4.1.1)<br>XenofilteR package from R (4.1.1)<br>STAR<br>mosdepth (0.2.3)<br>kallisto (0.46.2) |

SNPsplit
bowtie2 (2.4.4)
CellRanger version 7.0.1
featureCounts (v2.0.1)
DESeq2 (vl.26.0)
bedtools genomecov
MarkDuplicates from picard (2.23.5)
l0X Genomics Cell Ranger (v4.0.0)
biomaRt (v2.64.3)
Seurat (v4.0.1)
TTrimGalore (v.0.6.5)
DESEQ2 implementation within SeqMonk (vl.47.2)
ChlPseeker (vl.30.3)
samtools idxstats (1.13)
bedtools (v2.29.2)
deeptools (v3.43)
deeptools (3.5.0)
WashU epigenome browser (v5)
Seqmonk (vl.47.2)
MaxQuant (vl.6.6.0)
Perseus (vl.5.0.0)
Cytoscape (v3.7.2)
Progenesis QIP 4.2. (Nonlinear Dynamics, Waters)
Mascot Daemon (v2.6.0)
MSqRob (v0.7.6)
ZEN black (software version, Carl Zeiss Microscopy GmbH)
IMARIS software
Imaris Converter 10.2.0 (Oxford Instruments)
Codes pertaining to analyses in this study are available from GitHub webpages: https://github.com/pasquelab/Human-XIST-XCI_AP

For manuscripts utilizing custom algorithms or software that are central to the research but not yet described in published literature, software must be made available to editors and reviewers. We strongly encourage code deposition in a community repository (e.g. GitHub). See the Nature Portfolio guidelines for submitting code & software for further information.

# Data

Policy information about availability of data

All manuscripts must include a data availability statement. This statement should provide the following information, where applicable:

- Accession codes, unique identifiers, or web links for publicly available datasets
- A description of any restrictions on data availability
- For clinical datasets or third party data, please ensure that the statement adheres to our policy

Raw and processed sequencing data (scRNA-seq, bulk RNA-seq, CUT&RUN and MeD-seq) have been submitted to the NCBI GEO under accession number GSE261711. Reviewer token: ensdioeidxulbmj
This paper analyzes eXISTing, publicly available data. The accession numbers for the datasets are listed in the key resources table. All analysis code is available at: https://github.com/pasquelab/Human-XIST-XCI_AP.

Any additional information required to reanalyze the data reported in this paper is available from the lead contact upon request.

# Research involving human participants, their data, or biological material

Policy information about studies with human participants or human data. See also policy information about sex, gender (identity/presentation), and sexual orientation and race, ethnicity and racism.

| | |
|---|---|
| Reporting on sex and gender | *Use the terms sex (biological attribute) and gender (shaped by social and cultural circumstances) carefully in order to avoid confusing both terms. Indicate if findings apply to only one sex or gender; describe whether sex and gender were considered in study design; whether sex and/or gender was determined based on self-reporting or assigned and methods used.* *Provide in the source data disaggregated sex and gender data, where this information has been collected, and if consent has been obtained for sharing of individual-level data; provide overall numbers in this Reporting Summary. Please state if this information has not been collected.* *Report sex- and gender-based analyses where performed, justify reasons for lack of sex- and gender-based analysis.* |
| Reporting on race, ethnicity, or other socially relevant groupings | *Please specify the socially constructed or socially relevant categorization variable(s) used in your manuscript and explain why they were used. Please note that such variables should not be used as proxies for other socially constructed/relevant variables (for example, race or ethnicity should not be used as a proxy for socioeconomic status).* *Provide clear definitions of the relevant terms used, how they were provided (by the participants/respondents, the researchers, or third parties), and the method(s) used to classify people into the different categories (e.g. self-report, census or administrative data, social media data, etc.)* *Please provide details about how you controlled for confounding variables in your analyses.* |

| | |
|---|---|
| Population characteristics | *Describe the covariate-relevant population characteristics of the human research participants (e.g. age, genotypic information, past and current diagnosis and treatment categories). If you filled out the behavioural & social sciences study design questions and have nothing to add here, write "See above."* |
| Recruitment | *Describe how participants were recruited. Outline any potential self-selection bias or other biases that may be present and how these are likely to impact results.* |
| Ethics oversight | *Identify the organization(s) that approved the study protocol.* |

Note that full information on the approval of the study protocol must also be provided in the manuscript.

# Field-specific reporting

Please select the one below that is the best fit for your research. If you are not sure, read the appropriate sections before making your selection.

[x] Life sciences [ ] Behavioural & social sciences [ ] Ecological, evolutionary & environmental sciences

For a reference copy of the document with all sections, see nature.com/documents/nr-reporting-summary-flat.pdf

# Life sciences study design

All studies must disclose on these points even when the disclosure is negative.

| | |
|---|---|
| Sample size | All the experiments were repeated at least two or three times, and with cells with different genetic backgrounds, with exceptions. Sequencing experiments were done once, with or without replicates. Aside from the scRNA-seq experiment, all other XIST iKD experiments, including IF, FISH, differentiation, flow cytometry, and brightfield imaging, were performed using three independent XIST iKD clones, with the exception of Annexin V flow cytometry data, where 2 clones were used. For the XIST iKD, three independent clones were used for all experiments related to Figures 4B-4E and 5F-5G. Experiments were repeated three times for 1C-1H and 3A-3F, once for 1B, 2A-2H, 4F-4L, and 5F-5G, and two times for all other figures. |
| Data exclusions | No data were excluded with the exception of cell filtering described in the scRNA-seq analysis section in the manuscript as well as here.<br>Cell with good sequencing reads were retained by adjusting the number of counts per cell (nCount_RNA) and the number of mapped genes per cell (nFeature_RNA) to only keep cells that were mostly mapped to the human GRCh38 (hg38) genome (nCount _RNA > 2200, nFeature_RNA >1500). Cells with more than 25% of mitochondrial counts were filtered out. After quality control, we obtained data for 64479 cells with 28091 cells on day 0, 26650 cells on day 4 and 9738 cells on day 8. |
| Replication | All of the main experiments listed on this manuscript have been successfully replicated at least twice as independent experiments. When this is not the case, this has been indicated in figure legends. Please see above sections. |
| Randomization | All samples were run in a randomized fashion. |
| Blinding | Experiments were not blinded. |

# Reporting for specific materials, systems and methods

We require information from authors about some types of materials, experimental systems and methods used in many studies. Here, indicate whether each material, system or method listed is relevant to your study. If you are not sure if a list item applies to your research, read the appropriate section before selecting a response.

## Materials & experimental systems

| n/a | Involved in the study | |
|---|---|---|
| [ ] | [x] | Antibodies |
| [ ] | [x] | Eukaryotic cell lines |
| [x] | [ ] | Palaeontology and archaeology |
| [ ] | [x] | Animals and other organisms |
| [x] | [ ] | Clinical data |
| [x] | [ ] | Dual use research of concern |
| [x] | [ ] | Plants |

## Methods

| n/a | Involved in the study | |
|---|---|---|
| [ ] | [x] | ChIP-seq |
| [ ] | [x] | Flow cytometry |
| [x] | [ ] | MRI-based neuroimaging |

## Antibodies

| | |
|---|---|
| Antibodies used | Rat monoclonal anti-GATA3 Thermo Fisher Cat#14-9966-82<br>Mouse monoclonal anti-Vimentin Abcam Cat#ab8978<br>Rabbit polyclonal anti-KLF17 Sigma-Aldrich Cat#HPA024629<br>Rabbit monoclonal anti-H2AK119ub Cell Signalling Cat#8240s<br>Mouse monoclonal anti-H3K27me3 Active motif Cat#61017 |

Ser5P Polymerase II Abcam Cat#ab5408
Annexin V Invitrogen Cat#11-800572
CD249 (ENPEP) BD Biosciences Cat#744872
Annexin V Apoptosis Detection Kit FITC Invitrogen Cat#88-8005-72
Rabbit polyclonal anti-H3K9me3 Active motif Cat#39062
Rabbit polyclonal anti-H3K27me3 Active motif Cat#39055
Rabbit monoclonal anti-H2AK119ub Cell Signaling Cat#8240S
Rabbit polyclonal anti-IgG Diagenode Cat#C15410206

| | |
|---|---|
| Validation | Rat monoclonal anti-GATA3 Thermo Fisher Cat#14-9966-82, 1 in 100<br>Mouse monoclonal anti-Vimentin Abcam Cat#ab8978, 1 in 100<br>Rabbit polyclonal anti-KLF17 Sigma-Aldrich Cat#HPA024629, 1 in 40<br>Rabbit monoclonal anti-H2AK119ub Cell Signalling Cat#8240s, 1 in 800<br>Mouse monoclonal anti-H3K27me3 Active motif Cat#61017, , 1 in 500<br>Ser5P Polymerase II Abcam Cat#ab5408, 1 in 500<br>Annexin V Invitrogen Cat#11-800572, , 1 in 100 for 1 million cells<br>CD249 (ENPEP) BD Biosciences Cat#744872, 1 in 100 for 1 million cells<br>Annexin V Apoptosis Detection Kit FITC Invitrogen Cat#88-8005-72<br>Rabbit polyclonal anti-H3K9me3 Active motif Cat#39062, 1 in 100 for 1 million cells<br>Rabbit polyclonal anti-H3K27me3 Active motif Cat#39055, 1 in 100 for 1 million cells<br>Rabbit monoclonal anti-H2AK119ub Cell Signaling Cat#8240S, 1 in 100 for 1 million cells<br>Rabbit polyclonal anti-IgG Diagenode Cat#C15410206, 1 in 100 for 1 million cells |

## Eukaryotic cell lines

Policy information about cell lines and Sex and Gender in Research

| | |
|---|---|
| Cell line source(s) | H9 hESCs (WiCell).<br>ICSIG-1 IPSC0028 hiPSCs (Sigma).<br>SIG-1, Wild-type IPSC0028 hiPSCs (Sigma).<br>Male and female mouse embryonic fibroblasts (MEFs) isolated from wild-type mouse B6 embryos.<br>WIBR2-MGT hESCs (Primed WIBR2-MGT was a gift from Dr. Rudolf Jaenisch and Dr. Thorold W. Theunissen) |
| Authentication | All cell lines in this study were authenticated via gene expression analysis. H9, SIG-1 and ICSIG-1 were authenticated by STR genotyping at KU Leuven, Belgium. |
| Mycoplasma contamination | Periodic mycoplasma contamination testing was carried out and confirmed the absence of mycoplasma contamination. |
| Commonly misidentified lines<br>(See ICLAC register) | No commonly identified cell lines were used in this study. |

## Animals and other research organisms

Policy information about studies involving animals; ARRIVE guidelines recommended for reporting animal research, and Sex and Gender in Research

| | |
|---|---|
| Laboratory animals | Wild-type male mouse embryos C57B6/J E14.5. Animals were kept with a maximum of 5 animals per cage and separated by sex. Illumination was controlled on a 14h light, 10h dark light cycle from 7h to 21h . Temperature was checked daily and should be 22±2°C Humidity in mouse rooms was checked daily and should be between 45-70% but can vary with weather conditions, especially in winter. |
| Wild animals | No wild animals were used in this study. |
| Reporting on sex | *Indicate if findings apply to only one sex; describe whether sex was considered in study design, methods used for assigning sex. Provide data disaggregated for sex where this information has been collected in the source data as appropriate; provide overall numbers in this Reporting Summary. Please state if this information has not been collected. Report sex-based analyses where performed, justify reasons for lack of sex-based analysis.* |
| Field-collected samples | No field-collected samples were used in this study |
| Ethics oversight | Work with human embryonic and induced pluripotent stem cells to model early human development was approved by the UZ/KU Leuven ethics committee (S52426, S64962, S66185, S66184 and S66375) and also by the Flemish Government (SBB 219 2020/0435). Work with mouse embryo for mouse embryonic fibroblast was approved by the UZ/KU Leuven ethics committee (P170/2019). Experiments were carried out using the female H9 hESCs obtained from the WiCell Research Institute. Research on human embryonic stem cells has been approved by Agence de la Biomédecine and informed consent was obtained from all subjects. |

Note that full information on the approval of the study protocol must also be provided in the manuscript.

# Plants

Seed stocks

NA

Novel plant genotypes

NA

Authentication

NA

# ChIP-seq

## Data deposition

☒ Confirm that both raw and final processed data have been deposited in a public database such as GEO.

☒ Confirm that you have deposited or provided access to graph files (e.g. BED files) for the called peaks.

Data access links
*May remain private before publication.*

Raw and processed sequencing data (scRNA-seq, bulk RNA-seq, CUT&RUN and MeD-seq) have been submitted to the NCBI GEO under accession number GSE261711. Reviewer token: ensdioeidxulbmj

Files in database submission

GSM8215698 TSC_H3K27me3_Cut&Run_Rep1
GSM8215699 TSC_H3K9me3_Cut&Run_Rep1
GSM8215700 TSC_H2AK199Ub_Cut&Run_Rep1
GSM8215701 TSC_IGG_Cut&Run_Rep1
GSM8215702 TSC_H3K27me3_Cut&Run_Rep2
GSM8215703 TSC_H3K9me3_Cut&Run_Rep2
GSM8215704 TSC_H2AK199Ub_Cut&Run_Rep2
GSM8215705 TSC_IGG_Cut&Run_Rep2
GSM8215706 EXMC_H3K27me3_Cut&Run_Rep1
GSM8215707 EXMC_H3K9me3_Cut&Run_Rep1
GSM8215708 EXMC_H2AK199Ub_Cut&Run_Rep1
GSM8215709 EXMC_IGG_Cut&Run_Rep1
GSM8215710 EXMC_H3K27me3_Cut&Run_Rep2
GSM8215711 EXMC_H3K9me3_Cut&Run_Rep2
GSM8215712 EXMC_H2AK199Ub_Cut&Run_Rep2
GSM8215713 EXMC_IGG_Cut&Run_Rep2
GSM8229337 H9 TSC H3K27me3 CUT&RUN
GSM8229338 CT27 TSC H3K27me3 CUT&Tag
GSM9081211 Clonal_TSC_H3K27me3_Rep1
GSM9081212 Clonal_TSC_H3K27me3_Rep2
GSM9081213 Clonal_TSC_IGG_Rep1
GSE261711_20250526_H2AK119Ub_EXMC_Xa.bw
GSE261711_20250526_H2AK119Ub_EXMC_Xi.bw
GSE261711_20250526_H2AK119Ub_PXGL_Xa.bw
GSE261711_20250526_H2AK119Ub_PXGL_Xi.bw
GSE261711_20250526_H2AK119Ub_TSC_Xa.bw
GSE261711_20250526_H2AK119Ub_TSC_Xi.bw
GSE261711_20250526_H3K27me3_EXMC_Xa.bw
GSE261711_20250526_H3K27me3_EXMC_Xi.bw
GSE261711_20250526_H3K27me3_PXGL_Xa.bw
GSE261711_20250526_H3K27me3_PXGL_Xi.bw
GSE261711_20250526_H3K27me3_TSC-A_Xa.bw
GSE261711_20250526_H3K27me3_TSC-A_Xi.bw
GSE261711_20250526_H3K27me3_TSC_Xa.bw
GSE261711_20250526_H3K27me3_TSC_Xi.bw
GSE261711_EXMC_H2AK119Ub.log2ratioigg.bw
GSE261711_EXMC_H3K27me3.log2ratioigg.bw
GSE261711_EXMC_H3K9me3.log2ratioigg.bw
GSE261711_README_file_for_all_sup_files.xlsx
GSE261711_TSC-A_H3K27me3_downsampled.log2ratioigg.bw

GSE261711_TSC_H2AK119Ub.log2ratioigg.bw
GSE261711_TSC_H3K27me3.log2ratioigg.bw
GSE261711_TSC_H3K9me3.log2ratioigg.bw
GSE261711_h9.wgs.snp.biallelic.PASS.Het.chrX.Phased.Xa.Xi.vcf.gz
GSE261711_info_snp_heterozygous_h9_wgs_chrX_unphased.txt.gz
GSE261711_rna_seq_countMatrix.wGeneNames.txt.gz
GSE261711_RAW.tar (http)(custom) TAR (of BW, MTX, TSV, TXT)
GSM9081214 Clonal_TSC_IGG_Rep2

Genome browser session (e.g. UCSC)

*Provide a link to an anonymized genome browser session for "Initial submission" and "Revised version" documents only, to enable peer review. Write "no longer applicable" for "Final submission" documents.*

## Methodology

Replicates

Two independent biological replicates for all samples.

Sequencing depth

The samples were sequenced on a NovaSeq 6000 instrument (ICGex - NGS platform, Paris, France) to generate 2X100 paired-end reads.
minimum length of 50 bp

Antibodies

Rabbit polyclonal anti-H3K9me3 Active motif Cat#39062
Rabbit polyclonal anti-H3K27me3 Active motif Cat#39055
Rabbit monoclonal anti-H2AK119ub Cell Signaling Cat#8240S
Rabbit polyclonal anti-IgG Diagenode Cat#C15410206

Peak calling parameters

Reads were trimmed using trim_galore (0.6.5) (https://github.com/FelixKrueger/TrimGalore/) with a minimum length of 50 bp and were then mapped to the human genome (hg38) using bowtie2 (2.4.4) 91 with the following parameters: --local --very-sensitive --no-unal --no-mixed --no-discordant --phred33 -L 10 -X 700. Duplicated reads were removed using MarkDuplicates from picard (2.23.5) (http://broadinstitute.github.io/picard/), and aligned reads were filtered for a minimum mapping quality of 10 using samtools (1.13) 92. In all naive hESC and capacitated datasets, reads originating from mouse embryonic fibroblasts (MEFs) were discarded using XenofilteR package from R (4.1.1) (Kluin et al., 2018). To create the correlation heatmap, mean read depth per 10kb windows of the X chromosome were obtained using mosdepth (0.2.3) 93 and then processed on R (4.3.2) to be normalized by library size and used to compute Spearman's rank correlation coefficient and for the 500 most variable regions for each histone mark.

Data quality

*Describe the methods used to ensure data quality in full detail, including how many peaks are at FDR 5% and above 5-fold enrichment.*

Software

trim_galore (0.6.5)
bowtie2 (2.4.4)
MarkDuplicates from picard (2.23.5)
samtools (1.13)
R (4.1.1)
mosdepth (0.2.3)
R (4.3.2)
macs2 (2.2.7.1)
bedtools (2.30.0)
kallisto (0.46.2)

# Flow Cytometry

## Plots

Confirm that:

- [x] The axis labels state the marker and fluorochrome used (e.g. CD4-FITC).
- [x] The axis scales are clearly visible. Include numbers along axes only for bottom left plot of group (a 'group' is an analysis of identical markers).
- [x] All plots are contour plots with outliers or pseudocolor plots.
- [x] A numerical value for number of cells or percentage (with statistics) is provided.

## Methodology

Sample preparation

hESCs were dissociated using Accutase (Sigma-Aldrich, A6964-100ML) into single cells by incubating for 5 minutes at 37°C. Before proceeding with the antibody staining, cells were washed twice with FACS buffer containing 1% BSA in PBS (Gibco, 10010-015). Fluorophore-conjugated antibodies were diluted at a ratio of 1:50, which is 1 µL of antibody in 50 µL of FACS buffer for around 50000 to 100000 cells, and incubated at 4°C in the dark for at least for 30 min. Cells were washed again with FACS buffer, passed through a 40 µM cell strainer (Corning, 352352340),.

Instrument

Samples were analyzed/sorted using a BD influx, Canto or Beckman cytoflex SRT.

Software

Acquisition were done using BDDiva software and analysis was done using the Flowjo software.

Cell population abundance

After the TSCs and EXMCs were sorted using the BD Influx, the samples were re-evaluated for purity by assessing their

respective cell-surface markers, and the purity was found to be nearly 100%.

| Gating strategy | For all the rounds experiment, unstained and single stained samples were included to draw the precise gating strategy and to use it for compensation wherever necessary. |
| --- | --- |

☒ Tick this box to confirm that a figure exemplifying the gating strategy is provided in the Supplementary Information.
