# Supplementary material for: XIST drives X-chromosome inactivation and safeguards female extraembryonic cells in humans
Source: Nat Commun. 2026 Oct 22;17:10738. doi: 10.1038/s41467-026-78394-z (PMC13691081; doi:10.1038/s41467-026-78394-z)
Supplement: Supplementary file 4 [file 41467_2026_78394_MOESM3_ESM.pdf]

Description of Additional Supplementary Files

File Name: Supplementary Data 1
Description: Differentially expressed genes found in the scRNA-seq time course, related to Figures 4, 5, S6, S7 and S8.

File Name: Supplementary Data 2
Description: Gene ontology terms found related to differentially expressed genes in the scRNA-seq time course analysis, related to Figures 4, 5, S6, S7 and S8.

File Name: Supplementary Data 3
Description: Differentially expressed genes separated by chromosome in the scRNA-seq time course analysis, related to Figures 4, 5, S6, S7, and S8.

File Name: Supplementary Data 4
Description: Post-scLinaX statistical comparisons using only genes informative in both cell types being compared.

File Name: Supplementary Movie 1
Description: Reconstructed Zstack 3D-SIM images of a segmented naive nucleus stained for XIST (Red), related to figure 1.

File Name: Supplementary Movie 2
Description: Reconstructed Zstack 3D-SIM images of a segmented EXMC nucleus stained for XIST (Red), related to figure 1.

File Name: Supplementary Movie 3
Description: Reconstructed Zstack 3D-SIM images of a segmented TSC nucleus stained for XIST (Red), related to figure 1
